# Application of a hierarchical enzyme classification method reveals the role of gut microbiome in human metabolism

**DOI:** 10.1186/1471-2164-16-S7-S16

**Published:** 2015-06-11

**Authors:** Akram Mohammed, Chittibabu Guda

**Affiliations:** 1 Department of Genetics, Cell Biology and Anatomy, University of Nebraska Medical Center, Omaha, NE, 68198, USA; 2 Bioinformatics and Systems Biology Core, University of Nebraska Medical Center, Omaha, NE, 68198, USA; 3 Department of Biochemistry and Molecular Biology, University of Nebraska Medical Center, Omaha, NE, 68198, USA; 4 Fred and Pamela Buffet Cancer Center, University of Nebraska Medical Center, Omaha, NE, 68198, USA

**Keywords:** enzyme classification, functional domain, structural domain, machine learning, gut microbiome, human gut metabolism, obesity, inflammatory bowel disease, metabolic pathways, metagenomics

## Abstract

**Background:**

Enzymes are known as the molecular machines that drive the metabolism of an organism; hence identification of the full enzyme complement of an organism is essential to build the metabolic blueprint of that species as well as to understand the interplay of multiple species in an ecosystem. Experimental characterization of the enzymatic reactions of all enzymes in a genome is a tedious and expensive task. The problem is more pronounced in the metagenomic samples where even the species are not adequately cultured or characterized. Enzymes encoded by the gut microbiota play an essential role in the host metabolism; thus, warranting the need to accurately identify and annotate the full enzyme complements of species in the genomic and metagenomic projects. To fulfill this need, we develop and apply a method called ECemble, an ensemble approach to identify enzymes and enzyme classes and study the human gut metabolic pathways.

**Results:**

ECemble method uses an ensemble of machine-learning methods to accurately model and predict enzymes from protein sequences and also identifies the enzyme classes and subclasses at the finest resolution. A tenfold cross-validation result shows accuracy between 97 and 99% at different levels in the hierarchy of enzyme classification, which is superior to comparable methods. We applied ECemble to predict the entire complements of enzymes from ten sequenced proteomes including the human proteome. We also applied this method to predict enzymes encoded by the human gut microbiome from gut metagenomic samples, and to study the role played by the microbe-derived enzymes in the human metabolism. After mapping the known and predicted enzymes to canonical human pathways, we identified 48 pathways that have at least one bacteria-encoded enzyme, which demonstrates the complementary role of gut microbiome in human gut metabolism. These pathways are primarily involved in metabolizing dietary nutrients such as carbohydrates, amino acids, lipids, cofactors and vitamins.

**Conclusions:**

The ECemble method is able to hierarchically assign high quality enzyme annotations to genomic and metagenomic data. This study demonstrated the real application of ECemble to understand the indispensable role played by microbe-encoded enzymes in the healthy functioning of human metabolic systems.

## Background

Enzymes represent a significant fraction of an individual proteome [1] and catalyze a variety of specific reactions in the cellular systems [2,3]. Hence, identification of the functions of an entire complement of enzymes in an organism helps generate the metabolic blueprint of that species. This will not only improve our understanding of defined cellular processes of individual species but also help study the metabolic interdependence of multiple species in an ecosystem such as the human gut microbiome.

The Enzyme Commission (EC) [4] has classified all enzymes based on the enzymatic reactions they catalyze. Each enzyme has an EC number, which is a hierarchical number that distinguishes enzymes by the type of reactions they catalyze. The EC groups all enzymes into six broad classes that include (1) oxidoreductases - catalyze oxidoreduction reactions; (2) transferases - catalyze the transfer of a chemical group from a donor to an acceptor; (3) hydrolases - catalyze the hydrolysis of various bonds; (4) lyases - enzymes that cleave bonds by means other than by hydrolysis; (5) isomerases - catalyze geometrical or structural changes within one molecule; and (6) ligases - catalyze the joining of two molecules coupled with hydrolysis of a pyrophosphate bond in ATP or a similar triphosphate. The EC classification system assigns a unique four-field number to each enzymatic activity (such as EC 1.2.1.3 for aldehyde dehydrogenase (NAD+)) where, the first three numbers (a.k.a. levels) of an EC number represent progressively finer description of the enzymatic reaction, while the last level mostly represents substrate specificity of a reaction [5,6].

Experimental characterization of the enzymatic reactions of all enzymes in a genome or a metagenome is a tedious and expensive task. With the exception of a few well-characterized genomes such as *Escherichia coli (E. coli) *and yeast, the fraction of experimentally annotated enzymes in many sequenced genomes is very small. The problem is more pronounced in the metagenomic samples where even the species are not adequately cultured or characterized. To address this problem, computational approaches, which can build accurate models from known data to predict the unknown data, have been employed. Such models have been widely used to predict protein functions and annotate newly sequenced genomes [7-10].

Several computational methods exist for predicting enzyme classes (refer to our review [11]). Many of them use electronically inferred annotations by transferring the annotations (EC number, enzyme name and the reactants) of a known enzyme to an unknown enzyme, if the features of unknown match with the known. Most of the methods differ by the type of features they use to match proteins, which broadly include amino acid composition [12], sequence or structure homology [13-15], domain composition [16,17] and sequence motifs [18]. A variety of machine learning (ML) and data mining algorithms, including nearest-neighbor methods [19], support vector machines (SVMs) [20,21], Bayesian [22,23] and ensemble approaches [24,25] have been employed to build models for enzyme classification. The performance of these methods varies based on the classification algorithm, input datasets and features used for model building. Nevertheless, most of the existing methods fail to predict EC levels 3 and 4 due to the increasing difficulty in predicting the finer levels in the hierarchy, thus offer only limited value to enzyme annotations. Many enzyme prediction methods exhibit a lack of balance between specificity and sensitivity; EzyPred [17] and EFICAz2.5 [24] offer limited sensitivity for certain enzyme classes and/or EC levels and run extremely slow with high false positive rates. Hence, the existing methods are inadequate for annotation of high-throughput sequences generated from genomic and metagenomic projects, warranting the need for developing a new computational method.

The current study requires the identification of all enzymes encoded by the gut microbiome in human because the enzymes encoded by the gut microbiota play an extensive role in the human metabolism. Human gut microbiome is the largest and most complex of all microbial communities that harbor human body, with a gene set that is about 150 times larger than that of the human gene set [26]. Human gut microbiome alone is estimated to contain about 1000-1500 different species [26,27], but a majority of them are yet to be characterized. These bacterial communities extensively contribute to human gut metabolism by complementing enzymes that are not encoded by the human genome, but are essential for digestion of complex polysaccharides, absorption, metabolism of amino acids and vitamins, shaping of the immunological environment and a wide range of other metabolic functions [28-30]. Changes in the composition of human microbiota have been linked to health conditions such as inflammatory bowel disease (IBD), antibiotic-resistant infections, obesity, colon cancer, symptomatic atherosclerosis and diabetes [31-33]. Hence, the identification and functional characterization of gut microbial enzymes is a very important step towards understanding the microbe-dependent component of the human metabolism.

In this study, we developed a new hierarchical enzyme classification method based on machine learning that accurately predicts if a protein sequence is an enzyme or a non-enzyme, and if an enzyme, what is the specific enzymatic reaction (class and subclasses) it carries out. We apply this method to identify the full enzyme complements of 10 sequenced genomes of model organisms, and also those of the microbial species in the metagenomic samples obtained from human gut microbiomes. The methodology developed in this project and its application to identifying the full complements of microbial enzymes has made it feasible to study the role of microbe-complemented enzymes in the human gut metabolism. To our knowledge, this study represents a novel and robust approach to studying the pathway level host-pathogen interactions in the human gut metabolism.

## Results and discussion

**Figure 1:**
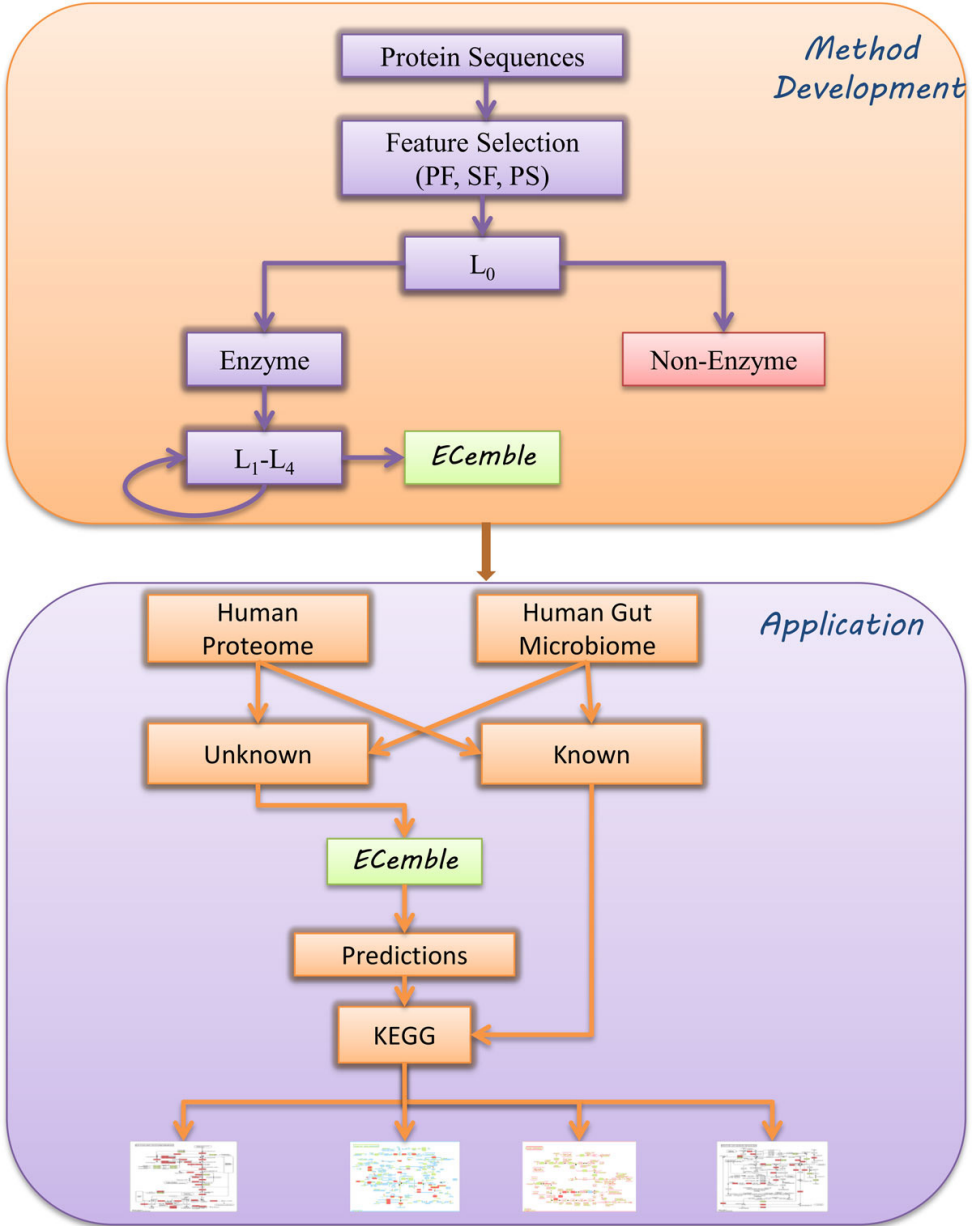
**Schematic representation of the ECemble method and its application**.

We describe the results and discussion in two separate sections that include the method development followed by its application to study the pathways in human gut metabolism. Figure 1 shows a schematic of the method and its application. An ensemble of five different machine-learning (ML) classifiers was used to build prediction models based on protein domains that include sequence or structure-derived features. Hence, this method is named as ECemble (**E**nzyme **C**lassification using ens**emble **approach). Bayesian network [22] model represents a set of random variables and their conditional dependencies, whereas Naïve Bayes [34] works under the strong assumption that there is independence among the features. K-nearest neighbor (KNN) [35] is 'instance based' learning algorithm. Despite its simplicity, it can offer very good performance. We tried polynomial and linear kernels in SVM [36] and polynomial kernel was found to be working better than the linear kernel. Random Forest classifiers (RFC) [37] work by generating large number of decision trees in a specific random way such that each one is de-correlated with the others. Each classifier model generates a probability distribution for all classes for each instance in the train and test set and the class with the highest probability is assigned as the predicted class.

This is a hierarchical prediction method that predicts enzymes and enzyme classes at 5 levels (henceforth referred to as L_0_, L_1_, L_2_, L_3 _and L_4_), where the first model at L_0 _predicts if a protein is an enzyme or a non-enzyme, and the subsequent models from L_1 _to L_4 _predict specific class and subclass of an enzyme in the EC number hierarchy. The predictions are selected by a consensus approach, i.e., only when at least two of the three top-performing ML classifiers show consistent predictions. To demonstrate its usefulness, we applied this method to predict the full complement of enzymes from ten sequenced proteomes. In addition, we also tested about 2.5 million protein sequences that were obtained from metagenomic sequencing and assembly of the human gut microbiome [26]. Using the full complements of enzymes from human and human gut microbiomes, we further investigated the role of microbe-derived enzymes in the human gut metabolism.

## Development of ECemble method

### Feature selection and optimization

We collected the known enzyme and non-enzyme protein sequences as positive and negative datasets, respectively. Sequences were clustered at 70% identity to generate 64,948 enzyme and 128,292 non-enzyme sequences, where each sequence has mapping to at least one of the following databases. These sequences were mapped against three domain databases that include Pfam [38], Superfamily [39] and Prosite [40] to extract the enzyme-specific and non-enzyme-specific features (as discussed in the Methods section). A protein domain is a conserved part of a protein's sequence or structure that can evolve, function, and exist independently of the rest of the protein chain. We used three domain databases that include Pfam, Superfamily and Prosite to represent the functional, structural and motif or active site regions of protein domains, respectively. These domains are used as features, where, in combination they offer a comprehensive feature set for ML methods to discriminate between enzymes and non-enzymes. We extracted 5,045 and 9,196 overlapping features for enzyme and non-enzyme datasets, respectively, from three domain databases (Table 1).

**Table 1:** Distribution of enzyme and non-enzyme features.

Feature Set	Pfam	Prosite (PS)	Superfamily (SF)	∑ (Pfam, PS, SF)
Database	12273	1308	1962	15543
Enzyme Only	859	133	111	1103
Non-Enzyme Only	7658	525	1013	9196
*Common features	2514	647	781	3942
Total unique features	11031	1305	1905	14241

*Common between enzyme and non-enzyme sequences

To facilitate the building of an effective ML-based classifier, it is essential to represent the domain features of each sequence in a binary format (feature vector), using '1' for the presence and '0' for the absence of a domain. The size of the feature vector matrix is N × ∑ (P, Q, R), where N is the total number of enzyme and non-enzyme sequences, and P, Q and R represent the total number of mapped domains in Pfam, Superfamily and Prosite, respectively (Table 2). In theory, the dimensionality of this vector gets very large; however, since each protein sequence contains only a few domains, we used the sparse-formatting option by storing the occurrence of features with their locations in the feature space to significantly speed up the model building process.

**Table 2:** Feature vectors and dimensionality for the dataset.

Dataset	Number of feature vectors	Data dimensionality
Enzyme Sequences	64948	5045x64948
Mixed^$^	193240	14241x193240

^$^Both enzyme and non-enzyme sequences

### Hierarchical design of prediction models

We applied five different machine-learning (ML) algorithms to best exploit features from the training dataset and optimize at each level. These include Naïve Bayes, k-Nearest Neighbor (KNN) classifier, Support Vector Machine (SVM), Decision Stump (DS) [41] and Random Forest (tree-based) classifiers (RFC). ML algorithms are employed to learn discriminative features of classes from the training data, build models, and test how related the unknown instances (testing data) are to these models. We used the WEKA (Waikato Environment for Knowledge Analysis) [42] framework to build prediction models in an iterative fashion at 5 different levels (L_0 _to L_4_). As part of the enzyme identification step, the first model at L_0 _predicts if a protein sequence is an enzyme or not. Only the sequences predicted as enzymes at L_0 _are forwarded to build models for predicting enzyme classes and subclasses at L_1_-L_4_, sequentially. At L_0, _there are only two classes (enzyme vs. non-enzyme) and similarly, at L_1_, there are only 6 enzyme classes; hence, one model is sufficient to predict classes at these two levels. However, the six enzyme classes at L_1 _are further divided into 51 subclasses at L_2_. As a result, 6 prediction models are constructed to predict 51 subclasses at L_2_. Similarly, due to the increasing number of subclasses at each subsequent level, 51 and 169 models are constructed to predict 169 and 1,921 subclasses at L_3 _and L_4_, respectively (Table 3). This hierarchical design of prediction models ensures that subclasses of a superclass are not predicted outside of that superclass and hence minimize false positives. For instance, members of EC 1.x.x.x superclass are never predicted as members of EC 2.x.x.x, and so on and so forth.

**Table 3:** Overall prediction accuracy of ECemble method.

EC Level	Number of Sequences	Correctly Predicted*	% Overall Accuracy	Classes	Model(s)	Average # of features per model
Level-0 (Enzymes)	193240 (64948)	188866 (62674)	97.74 (96.50)	2	1	14241
Level-1	62674	62167	99.19	6	1	5045
Level-2	62167	61721	99.28	51	6	811
Level-3	61721	60931	98.72	169	51	95
Level-4	60931	60199	98.80	1921	169	30

*Correctly predicted: Instances predicted by at least 2 of top 3 classifiers. Level-0 represents the model to predict if a protein sequence is an enzyme or not. Level 1-Level 4 represents corresponding levels of the EC number hierarchy.

### Evaluation of prediction performance

All feature vectors were randomly divided into 80 and 20 percent subsets for training and testing, respectively. Since the datasets are unbalanced across classes (and subclasses), class distributions are approximately preserved at all EC levels using stratified partitioning for training and testing sets. We used a two-step validation procedure that include determining a10-fold cross-validation accuracy on the training set, and the testing accuracy using the testing dataset that is not a part of the training data. We also report standard performance measures over each class at each level, including true positive rate (TPR), false positive rate (FPR), and receiver operating characteristic (ROC) curves and the area under the curve (AUC). Please refer to the Methods section for more details.

Table 4 shows the 10-fold accuracy and the testing accuracy for each of the five ML algorithms (Additional file 1: Figure S1). Overall, these two accuracies are consistent across the five classifiers indicating that models are not over trained. With the exception of decision stump (DS) classifier, the other four classifiers achieved at least 92.5% and 95.9% testing accuracies at L_0 _and L_1_, respectively. The three top performing classifiers are KNN, SVM and RFC, where testing accuracies reached at least 94.4% and 97.3% at L_0 _and L_1_, respectively. At the same time the false positive rates are very low; at L0 and L1, the FPR for the top three methods ranges from 0.054 to 0.061 and 0.011 to 0.026, respectively. 

**Table 4:** Ten-fold cross validation and testing accuracy for enzyme identification and enzyme classification.

	Enzyme Identification (EC L_0_)		Enzyme Classification (EC L_1_)	
**Classifiers**	**Ten-fold** Accuracy*	**Testing** **Accuracy**	**Ten-fold** Accuracy*	**Testing** **Accuracy**

DS	66.39	66.39	39.12	39.31
NBC	92.60	92.46	96.11	95.88
KNN	94.38	94.38	97.80	97.56
SVM	95.69	94.86	98.34	98.39
RFC	98.42	94.60	97.50	97.28

*Ten-fold cross validation accuracy. At EC L0 and EC L1 using ML classifiers, Decision Stump (DS), Naïve Bayes Classifier (NBC), K-Nearest Neighbor (KNN), Support Vector Machine (SVM), and Random Forest Classifier (RFC). At EC L0, train and test sets contain 154,592 and 38,648 sequences respectively, whereas EC L1 contain train and test sets of 50,139 and 12,535, respectively.

**Figure 2:**
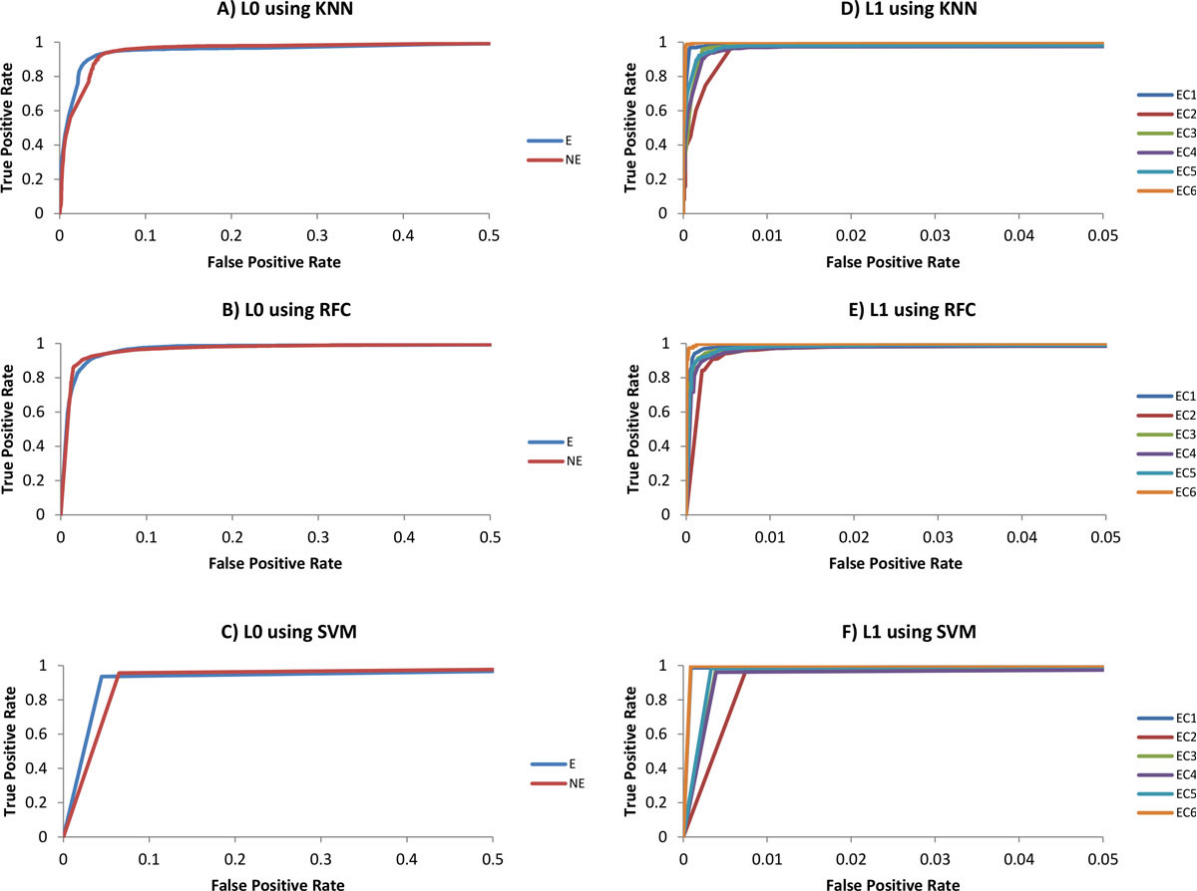
**ROC curve at Level-0 and Level-1 using top 3 performing classifiers**. (A) Testing at Level-0 using KNN, (B) Testing at level-0 using RFC, (C) Testing at Level-0 using SVM, (D) Testing at Level-1 using KNN, (E) Testing at level-1 using RFC, (F) Testing at Level-1 using SVM. Due to the high accuracies, False Positive Rate (X-axis) is shown till 0.5 for Level-0 and till 0.05 for Level-1.

Figure 2 illustrates ROC curves that show the relationship between TPR (sensitivity) and FPR (1-specificity) for a single class. An ideal ROC curve heads straight up on the Y-axis and then to the right parallel to the X-axis of the graph; thus maximizing the area under the curve (AUC). Such curves indicate that the classifier is predicting maximum true positives with minimum false positives, with AUC values closer to one. Figure 2 shows fairly consistent ROC curves for the top performing three ML methods at L_0 _and L_1_, respectively. At L_1_, enzymes from EC6 class are consistently the best predicted, while those from EC2 class showed relatively the least performance. This is probably because EC2 is one of the largest classes with most divergent subclass distribution, while EC6 has the least number of subclasses. The minimum AUC values at L_0 _and L_1 _are 0.945 and 0.989, respectively, indicating the superior performance of these three classifiers; hence, we chose only these three classifiers (KNN, SVM, RFC) for further use in this study.

### Consensus-based ensemble approach

A consensus approach adds confidence to the prediction accuracy and drastically reduces the false positives; hence, we implemented it by considering only those predictions, where the same enzyme class is predicted for a sequence by at least two out of the top three classifiers. Table 3 shows the overall prediction accuracy for testing data at each level (L_0_-L_4_) after using the consensus approach. We achieved an overall accuracy of 97.7% at L_0 _for identifying enzymes and non-enzymes, and at least 98.7% accuracy using models at L_1 _to L_4 _for predicting enzyme classes and subclasses. These results are very promising despite the fact that the size of the training data per model kept diminishing as the number of models increase at the lower levels (L_2_-L_4_). It can also be noted that at L_0_, 96.7% of the correctly predicted instances are consistent across the top three classifiers, while at L_1_-L_4_, over 99% of the correctly predicted instances are consistent (Additional file 2: Table S1).

### Effect of sequence identity in the training dataset

Sequence redundancy in the training datasets often results in overtraining and inaccurate estimation of prediction accuracy. To test the effect of sequence identity on the prediction accuracy, we created four datasets at 70%, 60%, 50% and 40% sequence identities using the CD-HIT clustering algorithm [43] and accordingly labeled as cdh70, cdh60, cdh50, and cdh40. At lower sequence identity thresholds like 40%, more sequences got removed resulting in a fewer number of sequences in each enzyme class compared to datasets with higher sequence identities. Similarly, the number of enzyme classes containing minimum number of sequences (ten) for model building started to go down from thresholds 70 to 40 percent identity (Additional file 3: Figure S2A). We generated the enzyme prediction models for EC levels L_0_-L_4 _for all the 4 datasets (shown in Additional file 4: Table S2). We needed at least 10 sequences in each subclass to build models using 10-fold cross validation. Accuracy is the highest for the cdh70 dataset (70% sequence identity) compared to all other datasets (cdh60, cdh50, cdh40) (Additional file 3: Figure S2B); hence, we used this dataset for model building. The 70% sequence identity is considered an optimal threshold in many other ML datasets, because the enzyme function starts to diverge quickly when the sequence identity is below 70% [5].

### Comparison of ECemble with other methods

**Figure 3:**
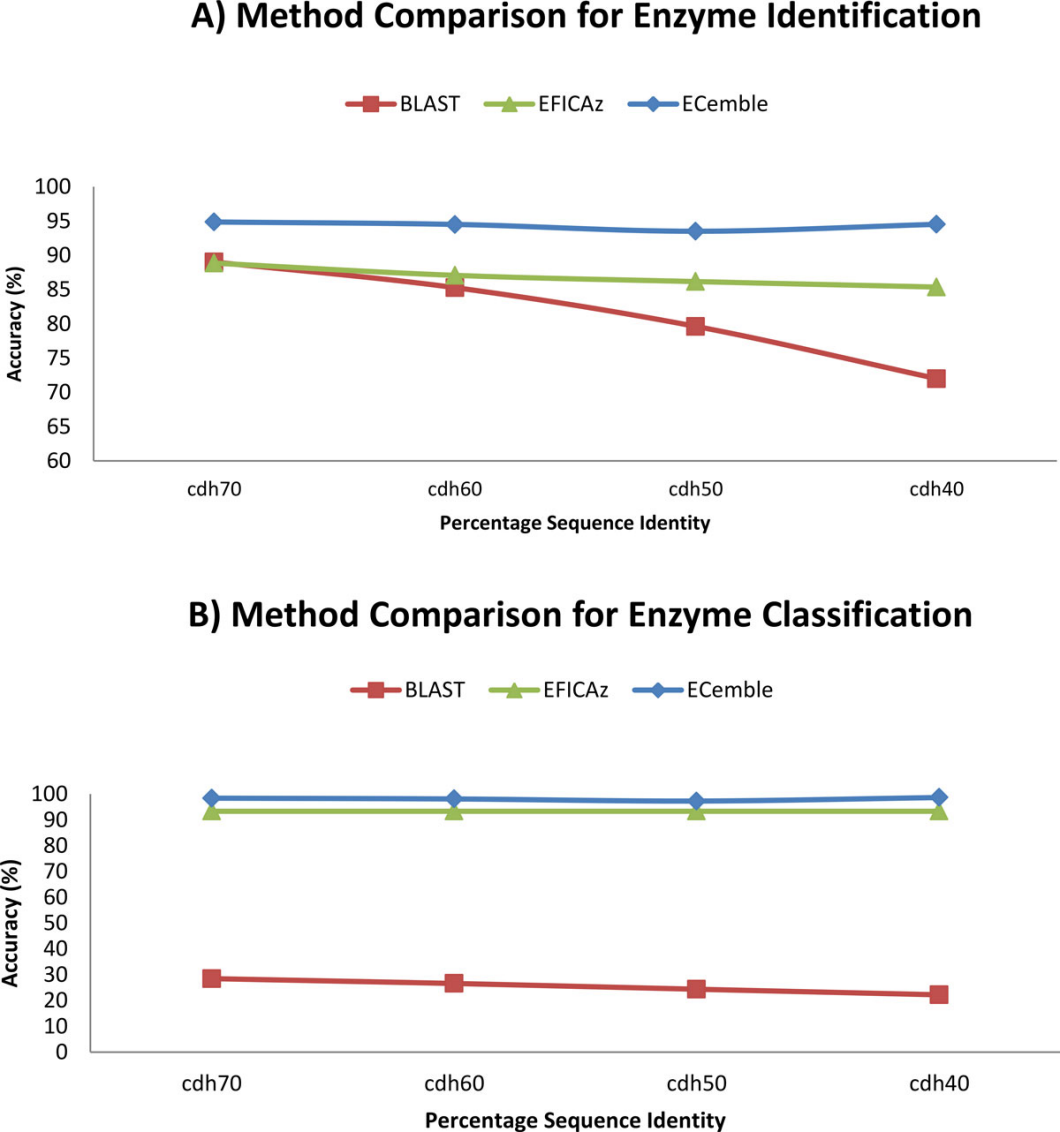
**Comparison of ECemble with BLAST and EFICAz methods**.

We compared the performance of ECemble with that of two existing methods, BLAST and EFICAz [24]. We chose these two methods because a number of methods are BLAST-based homology searching methods and EFICAz is a popular open-source tool. We used the same train and test datasets against these three methods to compare their performance. In the first step, a BLAST database was created with train dataset and the test data was queried against it for enzyme identification. Because BLAST generates a number of hits for each query with varying levels of confidence, only the top hit was considered (with a minimum E-value threshold of 10^-5 ^for blastp) as the prediction for each sequence in the test set. To predict the enzyme classes and subclasses, we performed a second query against a BLAST database created using only the enzyme sequences and used the same procedure as in the first step. Similarly, EFICAz method was trained and tested for both enzyme identification and classification. We also used four different clustered datasets (using CD-HIT) that were described earlier. For enzyme identification, ECemble reports the highest accuracy (94.9%) compared to BLAST (89%) and EFICAz (88.9%) using cdh70 dataset (Figure 3A). It can also be seen that the accuracy goes down as the percent identity in the datasets goes down from 70 to 40; however, this effect is more pronounced in the BLAST method compared to ECemble and EFICAz suggesting that reduced identity has minimal effect on these two methods. Similarly for enzyme classification at L_1_, the overall accuracy of ECemble (98.4%) is better than BLAST (28.43%) and EFICAz (93.36%) methods using cdh70 dataset (Figure 3B). We compared four different sequence identity thresholds (70%, 60%, 50% and 40%) of this dataset and our method performed better than others irrespective of the dataset used. These results convincingly demonstrate that the ECemble method consistently performed better than BLAST and EFICAz methods at all identity thresholds (70%, 60%, 50%, 40%), and that it is also suitable for accurate annotation of protein sequences with low sequence identity. Hence, in the next section, we used ECemble to identify and annotate complete enzyme complements of the sequenced genomes and metagenomes and applied this method to study the role of gut microbiome-derived enzymes in human metabolism.

## Application of ECemble method

### Annotation of full complements of enzymes in sequenced genomes

**Figure 4:**
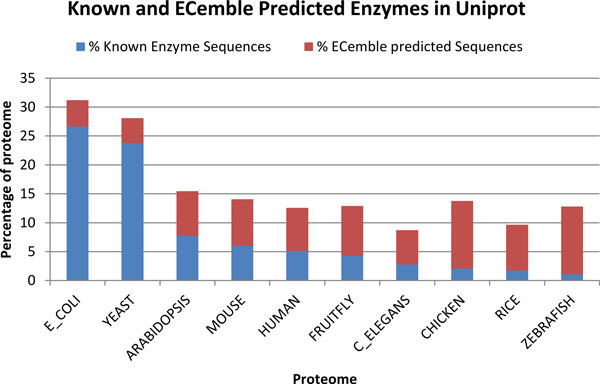
**Fractions of known and ECemble predicted enzymes in the proteomes of 10 model organisms from UniProt**. The data are sorted from highest to lowest known enzyme fractions. Both reviewed and unreviewed sequences from UniProt were used.

The ability of ECemble to predict solely based on a protein sequence enables it to annotate full complements of enzymes from sequenced genomes as well as from mixed genomes such as metagenomic samples. We applied ECemble to annotate a large number of unknown or unclassified enzyme sequences from 10 proteomes (that include reviewed and unreviewed proteins from UniProt database [44]) of both eukaryotic and prokaryotic model organisms (Additional file 5). As seen in Figure 4, *E. coli *and yeast proteomes have a high fraction of known enzymes (27% and 24%, respectively) compared to less than 5% in most of the other species. In contrast, the fractions of ECemble predicted enzymes are smaller in *E. coli *and yeast (around 4.5%), but reach up to 12% in chicken and zebrafish proteomes. In human and mouse, the fractions of unknown or unclassified enzymes predicted by ECemble account to 7.4% and 8.1% of their proteomes, respectively. These data underscore an important and generic application of our method that is to annotate a large number of unknown enzymes and enzyme classes in the sequenced genomes.

We further investigated about 200 newly predicted enzymes from the reviewed human proteome sequences (from the SwissProt database [45]), that were either not known as enzymes or had partially annotated enzyme subclasses. These 200 enzymes map to 119 unique enzyme reactions (with unique EC numbers), of which, only 30 are not previously known to be in human. Table 5 lists these 30 enzymes with the current annotation in UniProt and the predicted annotation by ECemble. Note that despite the well-characterized nature of the reviewed proteins, ECemble method is able to complement or correct the existing enzyme annotations (marked with * in Table 5). For instance, human genes such as *FOXRED1 *(FAD-dependent oxidoreductase domain-containing protein 1) and *SCCPDH *(saccharopine dehydrogenase-like oxidoreductase) have been broadly known as oxidoreductases and accordingly labeled as 1.-.-.-, in the UniProt database. ECemble method predicted the specific reactions of these enzymes as malate dehydrogenase (quinone) [EC 1.1.5.4] and Saccharopine dehydrogenase (NADP(+), L-glutamate-forming) [EC 1.5.1.10], respectively. In certain cases (marked with ** in Table 5), ECemble predictions differ only at the subclass levels (L_2_-L_4_), while in a small number of cases (marked with *** in Table 5), the predictions also differ from the current annotations at the class level (L_1_). Hence, experimental validation of these predictions is worth pursuing in the future. Because ECemble prediction models are built only from reviewed enzyme sequences, the accuracy and coverage of prediction by our model will continue to improve as the newly characterized enzymes from experimental studies become available. These results prove that ECemble method that solely uses protein sequences for prediction, is highly promising for the identification and classification of full complements of enzymes in the sequenced genomes.

**Table 5:** ECemble predicted enzymes from reviewed human proteome.

**Gene Name**	**Accession**	**^$^UniProt Annotation**	**New ECemble Prediction**	**EC Description of** **Enzyme Function**
*DHRS7*	Q9Y394	1.1.-.-	1.1.1.n4*	(-)-trans-carveol dehydrogenase
*GFOD1*	Q9NXC2	1.-.-.-	1.1.1.n6*	D-chiro-inositol 3-dehydrogenase
*FOXRED1*	Q96CU9	1.-.-.-	1.1.5.4*	Malate dehydrogenase (quinone)
*ALOX12B*	O75342	1.13.11.-	1.13.11.12*	Linoleate 13S-lipoxygenase
*ALDH8A1*	Q9H2A2	1.2.1.-	1.2.1.16*	Succinate-semialdehyde dehydrogenase (NAD(P)(+))
*SCCPDH*	Q8NBX0	1.-.-.-	1.5.1.10*	Saccharopine dehydrogenase (NADP(+), L-glutamate-forming)
*TFB1M*	Q9UNQ2	2.1.1.-	2.1.1.182*	16S rRNA (adenine(1518)-N(6)/adenine(1519)-N(6))-dimethyltransferase
*METTL15*	A6NJ78	2.1.1.-	2.1.1.199*	16S rRNA (cytosine(1402)-N(4))-methyltransferase
*METTL15P1*	P0C7V9	2.1.1.-	2.1.1.199*	16S rRNA (cytosine(1402)-N(4))-methyltransferase
*PYROXD2*	Q8N2H3	1.-.-.-	2.1.1.74*	(FADH(2)-oxidizing)
*GOT1L1*	Q8NHS2	2.6.1.-	2.6.1.9*	Histidinol-phosphate transaminase
*FGGY*	Q96C11	2.7.1.-	2.7.1.16*	Ribulokinase
*ISPD*	A4D126	2.7.7.-	2.7.7.60*	2-C-methyl-D-erythritol 4-phosphate cytidylyltransferase
*GDPD3*	Q7L5L3	3.1.-.-	3.1.4.46*	Glycerophosphodiester phosphodiesterase
*TLL1*	O43897	3.4.24.-	3.4.24.21*	Astacin
*TASP1*	Q9H6P5	3.4.25.-	3.4.25.2*	HslU--HslV peptidase
*ADAL*	Q6DHV7	3.5.4.-	3.5.4.2*	Adenine deaminase
*IREB2*	P48200	None	4.2.1.33*	3-isopropylmalate dehydratase
*TYRP1*	P17643	1.14.18.-	1.14.11.13**	Gibberellin 2-beta-dioxygenase
*HSD11B2*	P80365	1.1.1.-	1.3.1.9**	Enoyl-[acyl-carrier-protein] reductase (NADH)
*NDOR1*	Q9UHB4	1.6.-.-	1.8.1.2**	Sulfite reductase (NADPH)
*ENDOV*	Q8N8Q3	3.1.26.-	3.1.21.7**	Deoxyribonuclease V
*ENPP5*	Q9UJA9	3.1.-.-	3.6.1.27**	Undecaprenyl-diphosphate phosphatase
*MPPED2*	Q15777	3.1.-.-	3.6.1.41**	Bis(5'-nucleosyl)-tetraphosphatase (symmetrical)
*THNSL2*	Q86YJ6	4.2.3.-	4.2.1.20**	Tryptophan synthase
*ALOXE3*	Q9BYJ1	5.4.4.-	1.13.11.12***	Linoleate 13S-lipoxygenase
*ABHD2*	P08910	3.1.1.-	2.3.1.31***	Homoserine O-acetyltransferase
*ABHD1*	Q96SE0	3.1.1.-	2.3.1.31***	Homoserine O-acetyltransferase
*ABHD3*	Q8WU67	3.1.1.-	2.3.1.84***	Alcohol O-acetyltransferase
*SDR42E1*	Q8WUS8	1.1.1.-	5.1.3.20***	ADP-glyceromanno-heptose 6-epimerase

^$^Current annotation is based on UniProt database

n Preliminary EC numbers include an 'n' as part of the fourth digit in ENZYME database

* Good predictions that complement the current annotations

** Predictions that differ from the current annotations only at the subclass level

*** Predictions that differ from the current annotations at the class level

### Prediction of enzymes from the gut microbiome

To understand the role played by the most densely colonized human microbiome (gut microbiome) in human metabolism, we would require a catalogue of all the enzymes that can potentially exist in the human gut environment. There are several hundreds to thousands of microbial species that inhabit human gut and many of them are unknown, under characterized or not culturable in laboratory conditions, which would have made our goal impossible to accomplish. However, Next-Generation Sequencing (NGS) data from metagenomic samples could be used for *de novo *assembly of microbial genomes, and consequently for the prediction of the translated proteomes from the assembled scaffolds. We applied the ECemble method on 2.5 million proteins from metagenomic sequences (discussed in the Methods section) and assigned 213,313 (8.53%) sequences to 513 distinct enzymatic reactions (Additional file 6). Of these, 276 reactions are also encoded in human, leaving 237 reactions that are exclusively encoded by human gut microbial genomes. Of these, 222 enzymes are known gut bacterial enzymes in SwissProt database, while the remaining 15 enzymes are newly predicted by the ECemble method.

### Role of gut microbial enzymes on human metabolism

Application of ECemble to identify the enzymes and enzyme classes in human and gut microbial species has enabled us to ask the following questions. Which gut microbial enzymes are involved in human metabolism? Which human pathways are partly or fully driven by microbe-derived enzymes? Are there any previously unknown microbial enzymes that are involved in human gut metabolism? Even though, gut microbiome can contains bacteria, fungi and other small eukaryotes, about 98% of the gut microbial enzymes used in this study originated from bacteria [26], hence we limited this study only to bacteria-derived enzymes. To answer these questions, we created 4 different sets of enzymes. (i) Known human enzyme reactions (with unique EC numbers) (1,271), (ii) Unknown human enzyme reactions that are predicted by ECemble (19), (iii) Known bacterial enzyme reactions in the gut microflora predicted by ECemble (222) and (iv) Unknown gut bacterial enzyme reactions that are predicted by ECemble (15). For human and bacteria, known enzymes were obtained from SwissProt and KEGG (Kyoto Encyclopedia of Genes and Genomes) [46] databases (Additional file 7 shows known human enzyme reactions).

We mapped both human- and gut bacteria-encoded enzymes to the KEGG reference pathways to identify 48 human metabolic pathways, where each pathway contains both human- and bacteria-encoded enzymes plus at least one of them is predicted by ECemble. We refer to them henceforth as gut microbe complemented (GMC) pathways. The first set (39 pathways) contains human enzymes that are complemented by known gut bacterial enzymes. This set serves to validate the known role of gut bacteria in human metabolism (Additional file 8: Figure S3). The second set (9 pathways) is same as the first one; in addition, contains predicted enzymes (previously unknown) from gut bacterial species. These nine GMC pathways reveal the role of newly discovered gut bacterial enzymes in human gut metabolism, which is made possible with the ECemble method (Additional file 9: Figure S4). All the 48 pathways are mapped with enzymes (EC numbers) using a color-coded format. Light red colored enzymes represent known human enzymes, pink represents unknown human enzymes that are predicted by ECemble, light green represents known bacterial enzyme reactions in the gut microflora predicted by ECemble and light blue represents unknown gut bacterial enzyme reactions that are predicted by ECemble.

**Figure 5:**
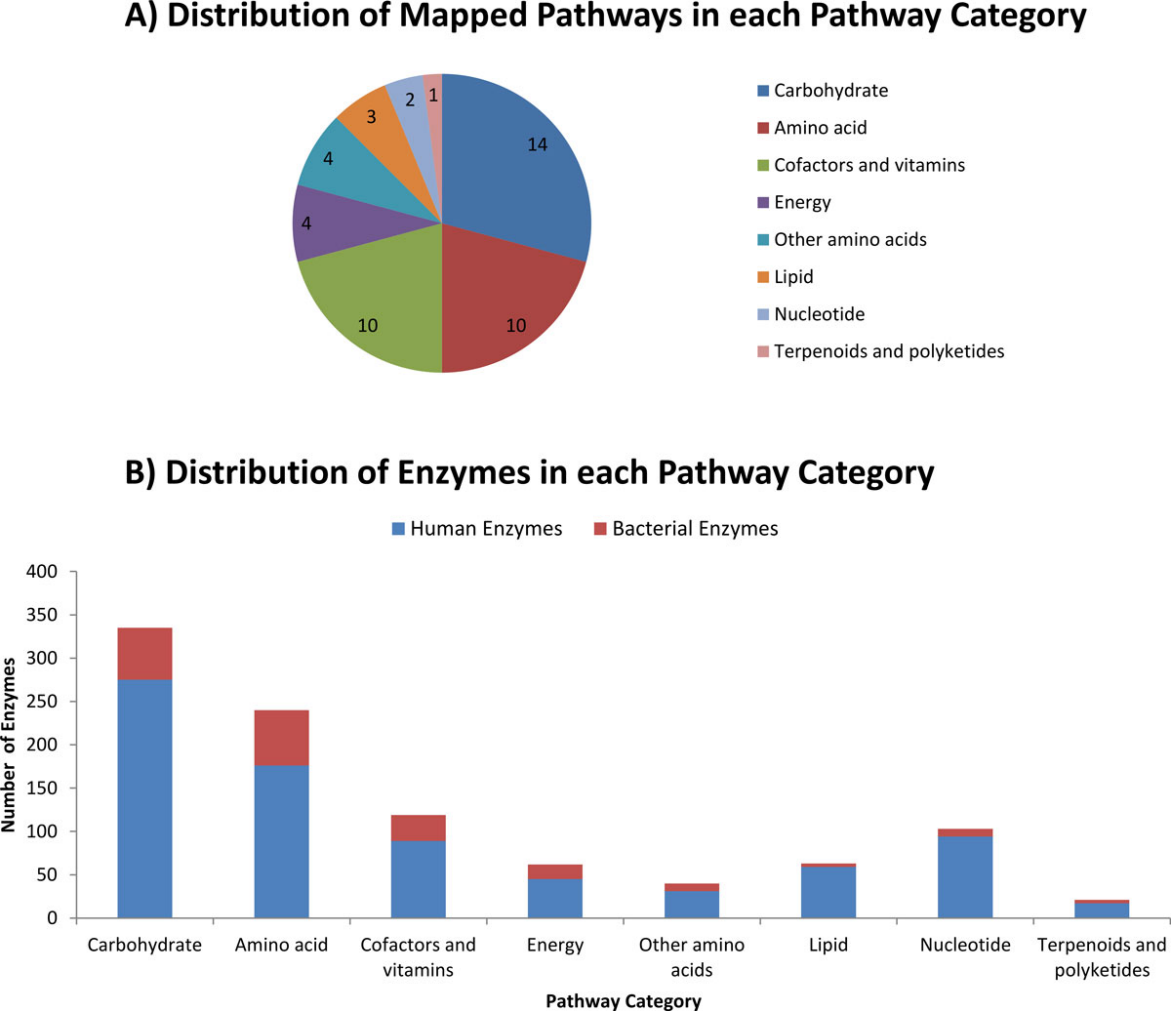
**Distribution of pathways and enzymes in each of the major pathway categories**. (A) Distribution of mapped pathways in each of the 8 major pathway categories (B) Distribution of human and bacterial enzymes in each of the 8 major pathway categories.

Functional distribution of GMC pathways is shown in Figure 5A and a full list of 48 pathways is given in Additional file 10. GMC pathways contribute to the metabolism of a variety of nutrients that primarily include carbohydrates, amino acids, vitamins and cofactors. Thus, the metabolic role of GMC pathways closely matches with the known nutritional requirements of humans. Our results show that gut microbial enzymes also play a role in lipid and energy metabolism and also in the metabolism of terpenoids, polyketides and derived amino acids such as taurine, D-glutamine, etc. Our method predicted 27 Carbohydrate active enzymes (CAZymes) (Additional file 11) in the human gut microbiome that include carbohydrate esterases, glycoside hydrolases, glycosyl transferases and polysaccharide lyases, which are primarily involved in carbohydrate metabolic pathways. As shown in Figure 5B, human- and microbe-derived enzymes complement different categories of human metabolic pathways. Note that 64, 60 and 30 exclusively microbe-derived enzymes complement the amino acid, carbohydrate and vitamin/cofactor metabolic pathways, respectively, which underscores their pivotal role as well as the dependency of human pathways on microbe-derived enzymes. Microbes not only complement, but also supplement some of the enzyme functions that are common to both human and microbes. For instance, we found that 518 enzymatic reactions are shared between human and gut-bacterial species; and we hypothesize that some of these common enzymes that are secreted by bacteria could supplement to perform human metabolic functions, and vice-versa. These common enzymes are highlighted in blue color in the Additional file 12. These results empirically support the argument that symbiotic gut microbiome has coevolved with human (or the coelomate animals) and they play a huge role in the metabolic interactions between host-gut microbiota [32].

Humans lack the enzymes needed to degrade oxalate [47]. It has been shown that the bacterial degradation of oxalate is carried by *Oxalobacter formigenes *in the human intestinal tract and the absence of *Oxalobacter formigenes *is considered a risk factor for urolithiasis [48]. Similarly Choline, an essential dietary nutrient, is found to be metabolized in the liver by the gut microbial enzymes. Thus conversion of dietary choline is used as a metabolic hallmark for liver and cardiovascular diseases [49]. Similarly, nitrate reductase that converts nitrate into nitrite and nitric oxide, is synthesized only by gut microbiome; elevated levels of nitric oxide have been associated with both IBD and obesity-induced insulin resistance [31]. Gut microbiome also plays a crucial role in the metabolism of xenobiotics; at least thirty commercially available drugs are shown to be metabolized as substrates by bacterial enzymes [50,51]. On the other hand, gut microbiome is also a source of inflammatory molecules such as lipopolysaccharide and peptidoglycan that may contribute to metabolic diseases [52].

### Gut microbe-complemented pathways for dietary carbohydrate metabolism

**Figure 6:**
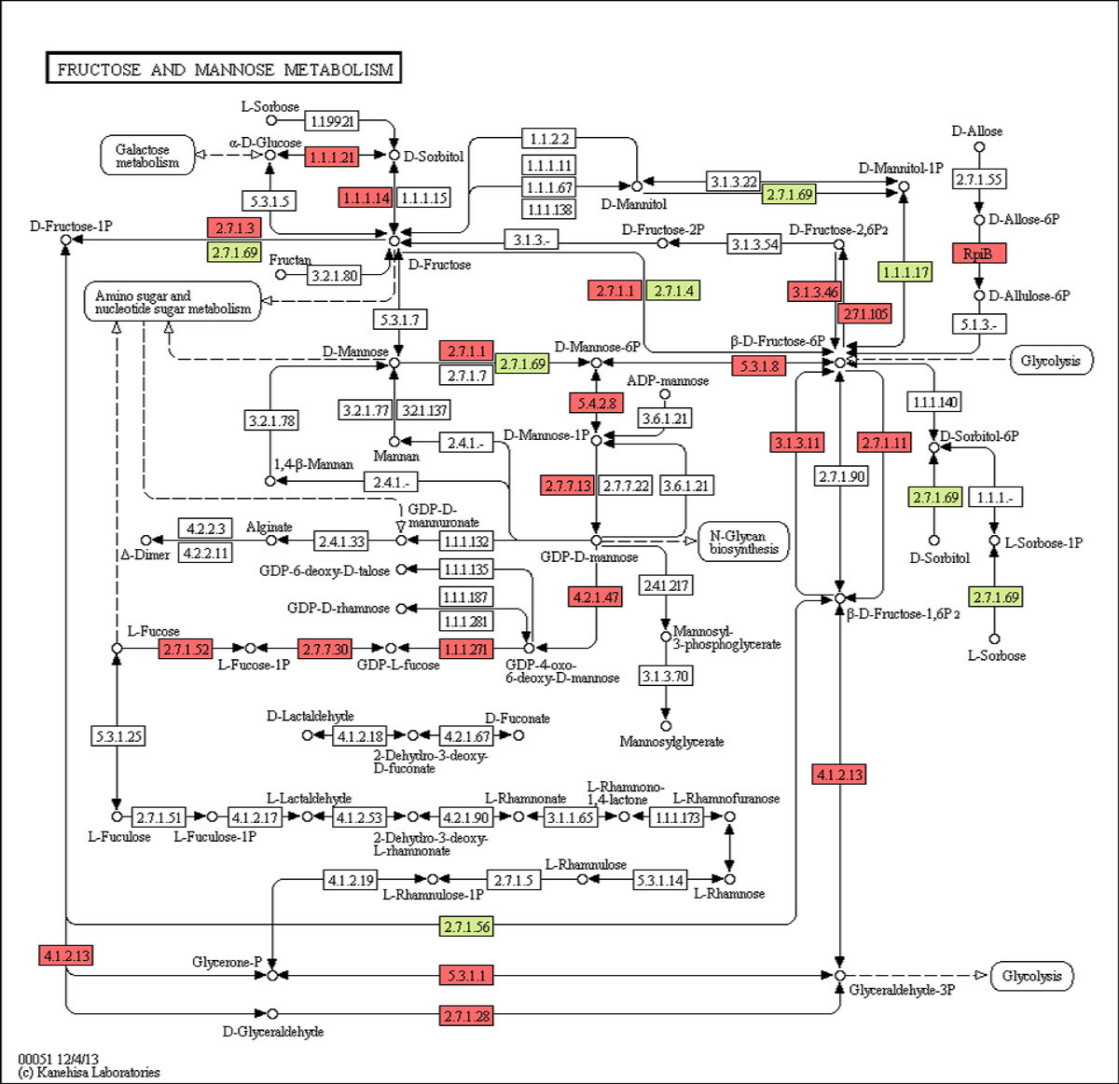
**Starch and sucrose metabolism pathway**. The following color coding scheme is used for the pathways: Known human enzyme reactions (Light Red), Unknown human enzyme reactions that are predicted by ECemble (Pink), Known bacterial enzyme reactions in the gut microflora predicted by ECemble (Light green) and Unknown gut bacterial enzyme reactions that are predicted by ECemble (Light Blue).

Carbohydrates are a major component of the human diet that includes starch (amylose and amylopectin) and disaccharides such as sucrose, lactose, and maltose. Human gut bacteria produce a vast panel of CAZymes to degrade components of dietary fiber into metabolisable monosaccharides and disaccharides [33]. The human genome encodes at best 17 digestive enzymes [53]; for ex. lactase, α-amylase, maltase, isomaltase and sucrose. It has been known that human enzymes can hydrolyze disaccharides (sucrose, lactose and maltose, etc.) and starch, but not other complex polysaccharides [54]. Hence, our ability to digest dietary plant carbohydrates resides entirely in our gut, where gut microbe-derived enzymes can hydrolyze complex dietary carbohydrates by producing a variety of CAZymes [55]. Thirteen gut bacterial enzymes predicted by ECemble were mapped in starch and sucrose metabolism pathway as shown in Figure 6. Enzymes responsible for the conversion of sucrose to glucose and bacterial degradation of pectin (a common component of dietary fibers) and xylan (polysaccharides in plant cell walls) are shown in the Table 6. These enzymes are predicted by our ECemble method from the gut microbial metagenomic data.

**Table 6:** Enzymes and their function in starch and sucrose metabolism pathway.

Function	Enzyme used	EC Number
Sucrose → Sucrose-6P	Protein-N(pi)-phosphohistidine--sugar phosphotransferase	2.7.1.69
Sucrose-6P → α-D-Glucose-6P (glucose)	Beta-fructofuranosidase	3.2.1.26
Pectin → Pectate	Pectinesterase	3.1.1.11
Pectate → Galacturonate	Polygalacturonase	3.2.1.15
Xylan → Xylose	Xylan 1,4-beta-xylosidase	3.2.1.37

**Figure 7:**
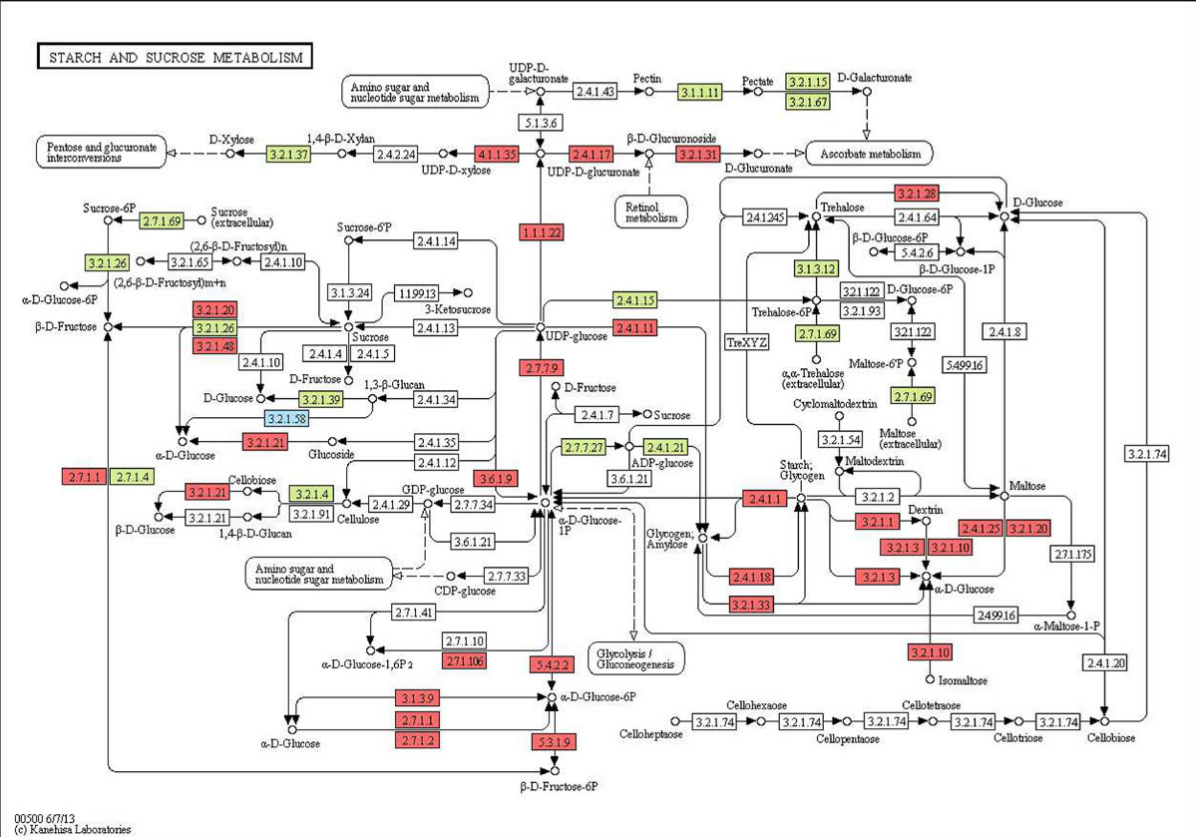
**Fructose and mannose metabolism pathway**. The following color coding scheme is used for the pathways: Known human enzyme reactions (Light Red), Unknown human enzyme reactions that are predicted by ECemble (Pink), Known bacterial enzyme reactions in the gut microflora predicted by ECemble (Light green) and Unknown gut bacterial enzyme reactions that are predicted by ECemble (Light Blue).

Another GMC pathway, fructose and mannose metabolism, explains how bacterial enzymes complement human enzymes to metabolize dietary sugars (Figure 7). Fructose occurs as a free monosaccharide and an isomer of glucose. In Figure 7, predicted bacteria-encoded enzymes and known human-encoded enzymes are shown, where D-Fructose (fructose) is catalyzed by bacterial enzymes, Protein-N (pi) -phosphohistidine-sugar phosphotransferase (2.7.1.69) and Fructokinase (2.7.1.4) into D-Fructose-1 Phosphate and β-D-Fructose-6 Phosphate, respectively. β-D-Fructose-6P is metabolized to Glyceraldehyde-3P using human-encoded enzymes Phosphofructokinase (2.7.1.11) and Fructose-biphosphate aldolase (4.1.2.13). The Glyceraldehyde-3P compound is a part of the glycolysis (normal metabolism of sugars) pathway, which is the main energy generating mechanism in the body. This pathway demonstrates that the bacteria- and human-encoded enzymes complement and work in unison in the digestion and energy metabolism pathways.

### Analysis of enzyme profiles of obese and IBD subjects

**Figure 8:**
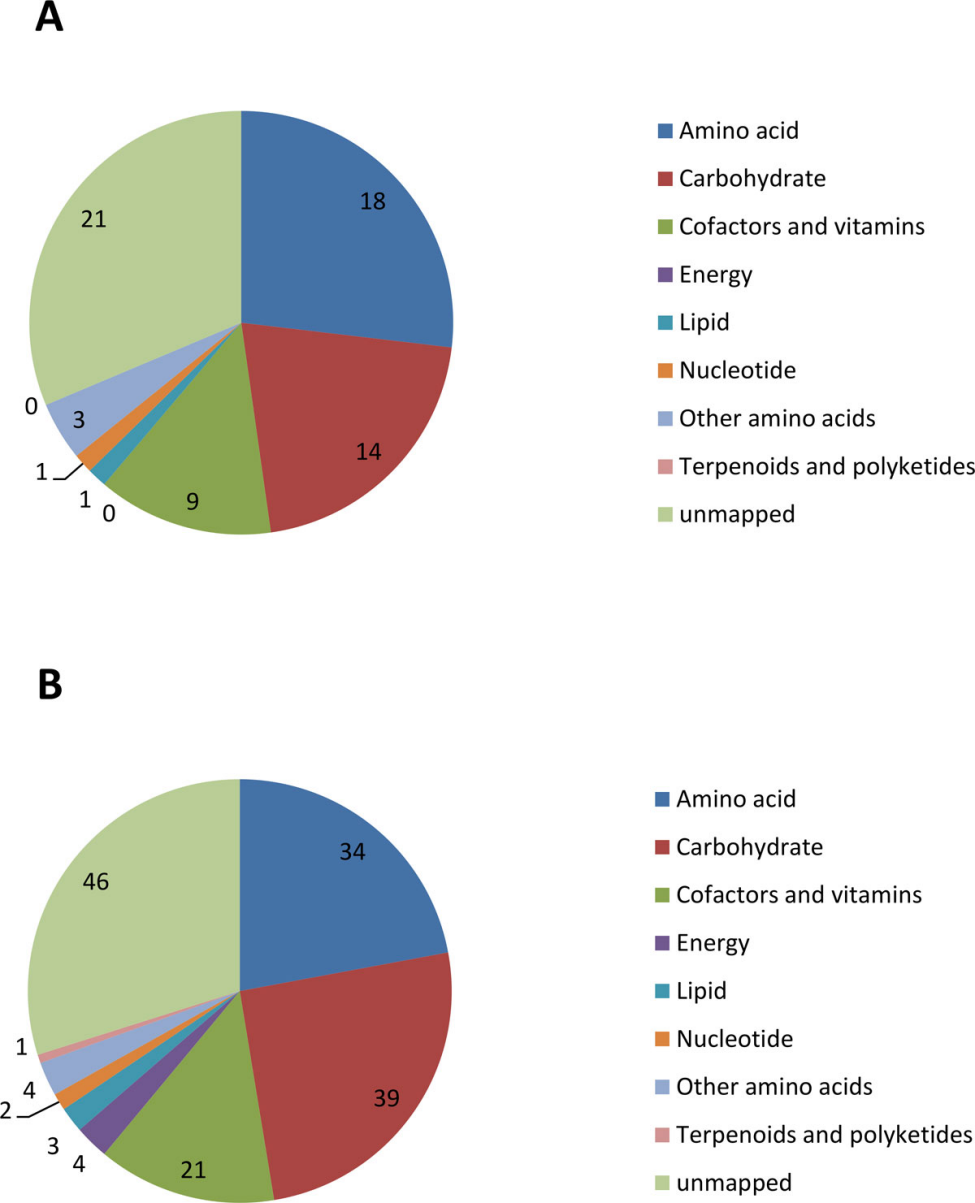
**Number of Obesity and IBD enzymes in each of the major KEGG pathway categories**. (A) Number of significant (p-value < 0.05; Fisher Exact Test) enzymes from obese vs. lean subjects in each major pathway categories (B) Number of significant (p-value < 0.05; Fisher Exact Test) enzymes from IBD vs. Non-IBD subjects in each major pathway categories.

A recent study published the metagenomic profiles of obese, lean and Inflammatory Bowel Disease (IBD) subjects [26]. This study also reported the translated gene products of gut microbiome from 124 metagenomic subject samples. We used these protein sequences to predict all the enzymes in each subject with our ECemble method, and analyzed the enzyme profiles of 'obese versus lean' (42 obese/82 lean) and 'IBD versus non-IBD' (25 IBD/99 Non-IBD) subjects. We identified 237 unique bacterial enzymes that are not encoded in human from the metagenomic samples of obesity, lean, IBD and non-IBD subjects. These include 222 known and 15 previously unknown enzymes in gut bacterial species. Details on how these enzyme reactions maps to KEGG human pathways are shown in Additional files 13 and 14. The taxonomic distribution of bacterial species from metagenomic samples is also shown in Additional file 15. The frequencies of enzymes present in the subjects were normalized based on the number of subjects in the obese/lean and IBD/non-IBD comparison groups and a Fisher's exact-test (P-value <0.05) using R [56] was conducted to determine the significantly enriched or depleted enzymes in the obese and IBD subjects (Additional files 16 and 17). Of the obesity-enriched enzymes, the most significant enzyme (P-value, 9.84E^-04^) is polygalacturonase (EC 3.2.1.15), which is encoded by *Bacteroides *and *Prevotella *species, and carries out pentose and glucuronate interconversions in starch and sucrose metabolism (Additional file 16). In contrary, urease (EC: 3.5.1.5) encoding bacteria are found in fewer number of obese subjects compared to lean subjects (obese/lean ratio = 0.88, P-value = 0.0179), suggesting that the loss or absence of this enzyme may be associated with obesity. In our analysis of IBD bacterial enzymes (Additional file 17), we found that non-IBD subjects predominantly host bacterial populations that contain an enzyme, Glucose-1-phosphate thymidylyltransferase (EC: 2.7.7.24, IBD/Non-IBD ratio= 0.06; P-value = 2.9E^-12^) compared to the IBD group. This enzyme is involved in the biosynthesis of L-rhamnose in bacteria. While there is no direct evidence to link lower levels of L-rhamnose to IBD, increased lactulose/L-rhamnose permeability ratio or decreased L-rhamnose in human intestinal permeability is found to be associated with IBD [57,58]. Hence, the consequences of the absence of bacterial populations that produce L-rhamnose in the IBD patients is worth investigating by experimental studies. The distribution of significant (p-value <0.05) enzymes in obese/lean and IBD/Non-IBD groups is presented in Figure 8. The comprehensive list of enzymes in each category (obese/lean and IBD/Non-IBD) is given in Additional files 16 and 17.

## Conclusions

A consensus-based ensemble method, ECemble, was implemented to identify enzymes from non-enzymes, and to hierarchically predict the class and subclasses of an enzyme up to L_4 _of the EC number. Comparison against BLAST and EFICAz methods showed superior performance of ECemble, both in coverage and accuracy. The superior coverage can be attributed to the generic protein domain feature space used in this method, while the improved accuracy resulted from the stringent consensus-based ensemble approach. Application of ECemble to predict full complements of enzymes from 10 sequenced genomes of model organisms has generated new annotations for unknown enzymes as well as full annotations for undercharacterized enzymes. Similarly, ECemble method enabled us to predict bacterial enzymes present in the gut microbiome and consequently use them to study the dependence of human metabolism on gut microbe-derived enzymes. Mapping of human and microbe-derived enzymes to KEGG metabolic pathways revealed that gut microbe-derived enzymes, especially those involved in the digestion of dietary nutrients, are essential components of a number of human pathways. Further application of this method to study the profiles of gut microbe-derived enzymes in lean versus obese, and IBD versus non-IBD subjects showed that certain enzymes were significantly enriched or depleted in these comparison groups, warranting further studies to understand the role of these enzymes on certain disease conditions. Two important merits of ECemble are that it can predict solely based on the protein sequence and also fully annotates enzymes by hierarchically assigning classes and subclasses up to L_4_. Hence, it can be a valuable tool for accurately annotating the entire enzyme complements of individual genomes as well as the mixed genomes from metagenomic studies. As evident from this study, ECemble can be effectively used to study the metabolic interactions between the host and microbes or those among the members of a community in a microflora. Being a generic method, it can be applied to study the systems level pathway interactions in any organism or microbial community.

## Methods

### ECemble dataset

The dataset include enzyme sequences taken from the EXPASY enzyme database [59], which is built based on SwissProt database [45] that contains reviewed and experimentally determined annotations on enzymes. We applied the following filters to obtain high-quality data for testing and training our program: duplicate sequences, sequences annotated as 'fragments' and those shorter than 50 residues in length were removed; sequences having more than one EC number were also removed. To avoid redundancy in the dataset, no more than 70% sequence identity was allowed among sequences within a class for each of the six EC classes, using the CD-HIT program [43]. Consequently, 64,950 non-redundant enzyme sequences (positive dataset) were collected that broadly cover all the known enzyme classes and subclasses. Similarly, a negative dataset (non-enzymes) of 128,475 sequences were collected from a SwissProt database by following the same filtering steps as mentioned above. A class-wise distribution of known enzyme sequences is listed in Table 7. Sequence to domain mapping was done for enzyme and non-enzyme sequences based on the Pfam [38] and Superfamily [39] databases using HMMSCAN [60], and the Prosite database using PSSCAN [61].

**Table 7:** Class-wise statistics on the number of enzyme sequences, and the subclasses at each level.

EC Level-1(6 Classes) (Example:1.x.x.x)	Total Sequences	Subclasses at EC Level-2 (example:1.1.x.x)	Subclasses at EC Level-3 (example:1.1.1.x)	Subclasses at EC Level-4 (example:1.1.1.1)
EC1: Oxidoreductases	8662	22	90	658
EC2: Transferases	23604	9	31	751
EC3: Hydrolases	15183	11	49	781
EC4: Lyases	5525	7	15	268
EC5: Isomerases	4146	6	17	127
EC6: Ligases	7830	6	11	109
Total	**64950**	**61**	**213**	**2693**

There are 6 major classes at Level-1 that expand into 61, 213 and 2,693 subclasses at Levels 2, 3 and 4, respectively.

### Feature databases

The most important features of enzymes that differentiate them from non-enzymes are their structure, function and catalytic sites. The feature set we used for machine learning in this study was based on the functional and structural domains, and sequence motifs. Structural domains define evolutionarily conserved region of proteins that can fold independently, while functional domains define evolutionarily conserved regions that can independently perform a specific biological function. Similarly, short sequence motifs define the catalytic or binding sites of enzymes. Machine learning algorithms exploit these different sets of features from enzyme and non-enzyme data in differentiating one from other.

Three popular domain databases - Pfam, Superfamily and Prosite - were used to extract the domain features of enzyme and non-enzyme sequences for building the ECemble method. Pfam (Protein Family) database contains functional domain information for protein sequences; Prosite database is a collection of biologically important sites, patterns and sequence motifs associated with protein functions; and the Superfamily database contains sets of homologous proteins that conserve structural and active site features. Together, these three databases provide a comprehensive coverage of functional, active site and structure-based features of enzyme and non-enzyme protein sequences.

### Machine learning methods

We selected five diverse and most popular machine learning classifiers; Naïve Bayes [34], k-Nearest Neighbor (KNN) [35] classifier, Support Vector Machine (SVM) [36], Decision Stump (DS) [41] and Random Forest (tree-based) classifiers (RFC) [37], to build models for enzyme identification and classification. We used the WEKA 3.7.5 [42] package, which is an open-source, Java-based framework to build classification models using different ML techniques.

### Ten proteome datasets

We downloaded the proteomes of ten model organisms from the SwissProt database [45], ranging from animal, plant, fungal and bacterial species such as *Saccharomyces cerevisiae *(yeast), *Caenorhibditis elegans *(C. elegans), *Drosophila melanogaster *(fruitfly), *Danio rerio *(zebrafish), *Gallus gallus *(chicken), *Mus musculus *(mouse), *Homo sapiens *(human), *Escherichia coli *(E. coli), *Oryza sativa *(rice) and *Arabidopsis thaliana *(Arabidopsis). We filtered out the experimentally known enzyme sequences from each of the ten proteomes and tested the unclassified sequences with ECemble.

### Human gut metagenomic samples

We used a study on the human gut microbiome from the Beijing Genomics Institute's (BGI) Metagenomic Sequencing Project [26] that describes a set of about 3.3 million microbial genes sequenced and assembled from fecal samples of 124 individuals (Additional file 18 represent the patient profiles and health status), to study the impact of human gut microbiome on human metabolism. As part of the filtering steps, sequences mapped to viruses, archaea, eukaryota, unclassified and unknown sequences were removed. About 2.5 million translated protein sequences from the assembled scaffolds of bacterial genomes were predicted by our ECemble method to identify all the bacterial enzymes in the human gut microbiome. The diversity of taxonomic groups of bacteria at each level is given in Additional file 19: Figure S5.

### KEGG database

We used Kyoto Encyclopedia of Genes and Genomes (KEGG) [46], the most comprehensive database source that integrates genomic, proteomic and systemic functional information for pathway analysis. The KEGG Pathway suite is a collection of manually drawn maps demonstrating the existing knowledge on the molecular interaction and reaction networks. KEGG pathways were used as reference pathways to map human and gut metagenomic enzymes for analysis of gut microbe-complemented human metabolic pathways.

### Performance measurements

We used standard evaluation metrics that include 10-fold cross validation and ROC curves. In 10-fold cross-validation, sequences at each level are divided into ten parts, models are built using nine parts, and predictions are generated and evaluated on the data contained in one part. This procedure is repeated ten times, where each part is tested against the models built from nine other parts. The average performance of the ten models is considered as an unbiased estimate of the training model performance. After cross-validation, we assessed the performance of the fully trained classifier models using the test set (20% of original data) that were hidden from the classifiers. We report standard performance measures over each enzyme class including the following: *true positives *(TP) as the number of sequences that are correctly identified in a class that belongs to them; *false negatives *(FN) as the number of sequences that are not identified in a class that belongs to them; *true negatives *(TN) as the number of sequences that are not found in a class that does not belong to them; *false positives *(FP) as the number of sequences that are identified in a class that does not belong to them; *sensitivity *as the proportion of true positives that are predicted as positives; *specificity *is the proportion of true negatives that are predicted as negatives. The sensitivity and specificity are given by, sensitivity = TP/ (TP + FN); specificity = TN/ (TN + FP).

We also report *accuracy *in a class as the ratio of the number of correctly predicted enzyme sequences to the total number of sequences in that class. We optimize and validate the accuracy of ECemble by selecting the optimal model(s) that has maximum true positive rate (sensitivity) and minimum false positive rate (1-specificity). Finally, we show *ROC *(Receiver Operating Characteristic) curves as a graphical means of measuring the performance for each class at each level of the prediction process, and the area under the curve (AUC) as a numeric measure of performance depicted by ROC curves.

## List of abbreviations used

AUC: Area Under Curve

DS: Decision Stump

EC: Enzyme Commission

IBD: Inflammatory Bowel Disease

KNN: K-Nearest Neighbor

KEGG: Kyoto Encyclopedia of Genes and Genomes

ML: Machine Learning

NBC: Naïve Bayes Classifier

RFC: Random Forest Classifier

ROC: Receiver Operating Characteristic

SF: Superfamily

SVM: Support Vector Machine

WEKA: Waikato Environment for Knowledge Analysis

## Competing interests

The authors declare that they have no competing interests.

## Authors' contributions

AM developed the ECemble method using machine-learning approaches and applied it to analyze gut metabolic pathways, and drafted the manuscript. CG conceived the idea of the project, provided overall guidance and coordination, suggested improvement and corrected the manuscript. All authors read and approved the final version of the manuscript.

## Supplementary Material

Additional file 1 Figure S1. 10-fold cross validation and testing accuracies. A) For enzyme identification at EC Level-0 using ML classifiers Decision Stump (DS), Naïve Bayes Classifier (NBC), K-Nearest Neighbor (KNN), Support Vector Machine (SVM), and Random Forest Classifier (RFC). B) For enzyme classification at EC Level-1 using ML classifiers Decision Stump (DS), Naïve Bayes Classifier (NBC), K-Nearest Neighbor (KNN), Support vector Machine (SVM), and Random Forest Classifier (RFC). Click here for file

Additional file 2 **Table S1**. Overall predictions of 2 classifiers vs. 3 classifiers. Click here for file

Additional file 3 Figure S2. Accuracy and distribution of enzyme sequence and class. A) Distribution of enzyme sequence and class coverage for cdh70, cdh60, cdh50 and cdh40 datasets. B) Accuracy at each EC level for cdh70, cdh60, cdh50 and cdh40 datasets. Click here for file

Additional file 4 **Table S2**. Accuracy of ECemble method using cdh70, cdh60, cdh50 and cdh40 datasets Click here for file

Additional file 5 **Statistics for Known and ECemble predicted enzyme sequences from ten proteomes**. The proteomes contains both reviewed and unreviewed sequences from UniProt (Data Sheet 'ReviewedAndUnreviewed'). The ECemble predictions for the reviewed sequences from 10 proteomes are given in Data Sheet 'PredictionsReviewedSet'. Click here for file

Additional file 6 **ECemble predicted gut microbial enzymes**. Click here for file

Additional file 7 **Known human enzyme reactions from SwissProt and KEGG**. Click here for file

Additional file 8 **Figure S3**. GMC pathways that validate the role of gut bacteria in human metabolism Click here for file

Additional file 9 **Figure S4**. GMC pathways that reveal the newly discovered role of gut bacteria in human metabolism Click here for file

Additional file 10 **Mapped human pathways in major pathway categories**. Click here for file

Additional file 11 **CAZymes found in metagenomic samples using ECemble**. Click here for file

Additional file 12 **Known enzymes common in both human and bacteria**. Click here for file

Additional file 13 Known bacterial enzyme reactions in the gut microflora predicted by ECemble Click here for file

Additional file 14 **Unknown gut bacterial enzyme reactions that are predicted by ECemble**. Click here for file

Additional file 15 **ECemble predicted gut bacterial enzymes with subject information**. Click here for file

Additional file 16 Obesity/Lean enzymes from metagenomic samples using Fisher-exact test. Obese_Normalized is calculated as the ratio of number of obese subjects having a particular enzyme to the total number of obese subjects (42). Similarly, Lean_Normalized is calculated as the ratio of number of lean subjects having a particular enzyme to the total number of lean subjects (82). Click here for file

Additional file 17 IBD/Non-IBD enzymes from metagenomic samples using Fisher-exact test. IBD_Normalized is calculated as the ratio of number of IBD subjects having a particular enzyme to the total number of IBD subjects (25). Similarly, Non-IBD_Normalized is calculated as the ratio of number of Non-IBD subjects having a particular enzyme to the total number of Non-IBD subjects (99). Click here for file

Additional file 18 **Metagenomic samples profile**. Click here for file

Additional file 19 **Figure S5**. Taxonomic distribution of bacterial species from metagenomic samples. Click here for file
