# Supplementary figures and images for: Application of a hierarchical enzyme classification method reveals the role of gut microbiome in human metabolism
Source: BMC Genomics. 2015 Jun 11;16(Suppl 7):S16. doi: 10.1186/1471-2164-16-S7-S16 (PMC4474468; doi:10.1186/1471-2164-16-S7-S16)

## A) Classifier Performance at EC Level 0

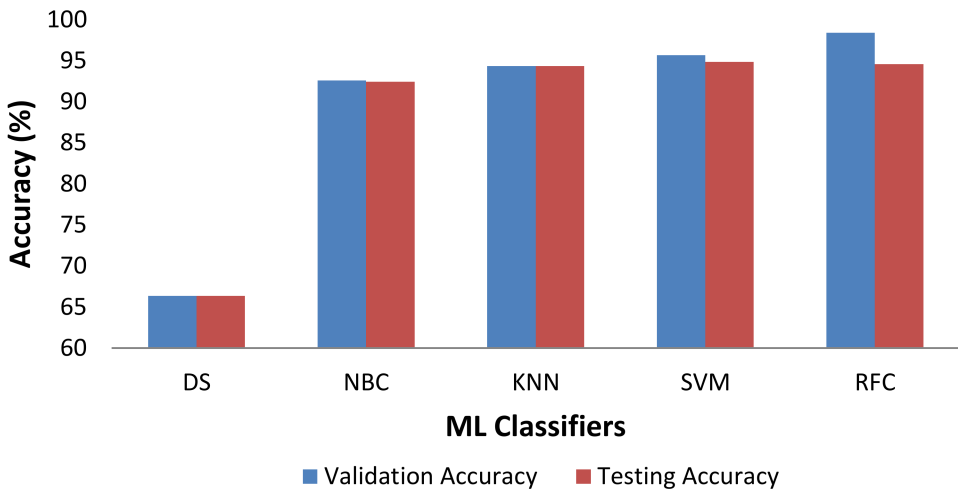

## B) Classifier Performance at EC Level 1

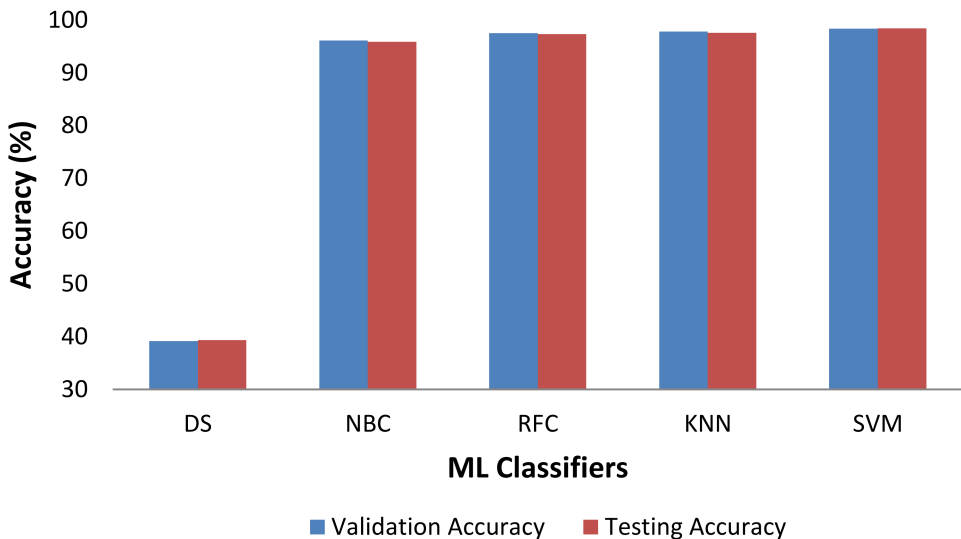

Supplement: Additional file 1 — Figure S1. 10-fold cross validation and testing accuracies. A) For enzyme identification at EC Level-0 using ML classifiers Decision Stump (DS), Naïve Bayes Classifier (NBC), K-Nearest Neighbor (KNN), Support Vector Machine (SVM), and Random Forest Classifier (RFC). B) For enzyme classification at EC Level-1 using ML classifiers Decision Stump (DS), Naïve Bayes Classifier (NBC), K-Nearest Neighbor (KNN), Support vector Machine (SVM), and Random Forest Classifier (RFC). [file 1471-2164-16-S7-S16-S1.pdf]

## A) Distribution of Enzyme Sequence and Class

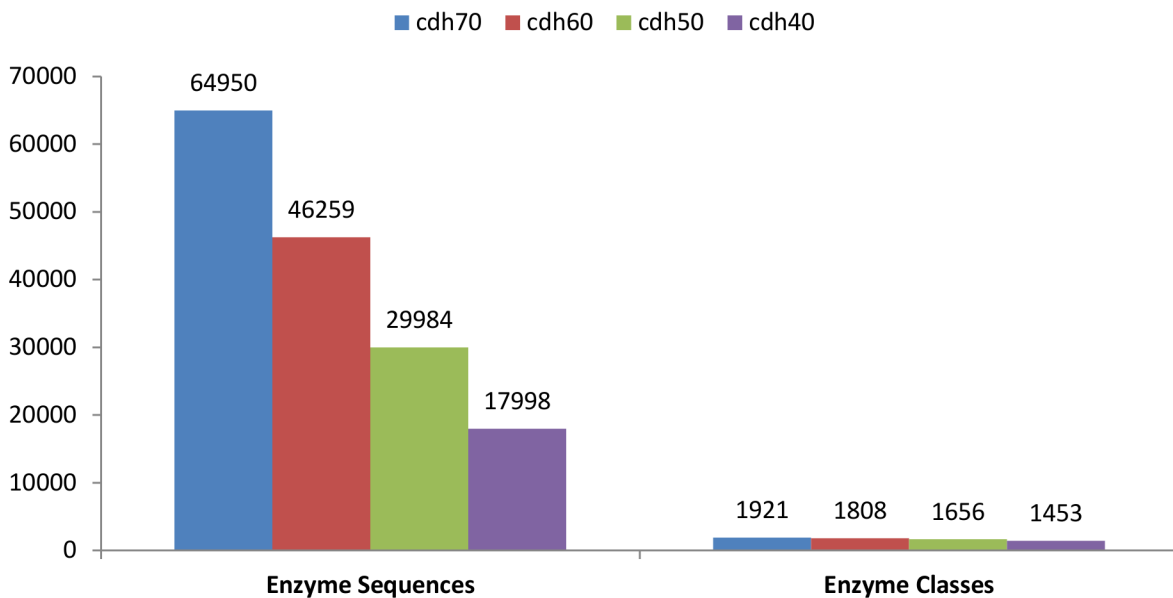

## B) Accuracy of models at each EC Levels

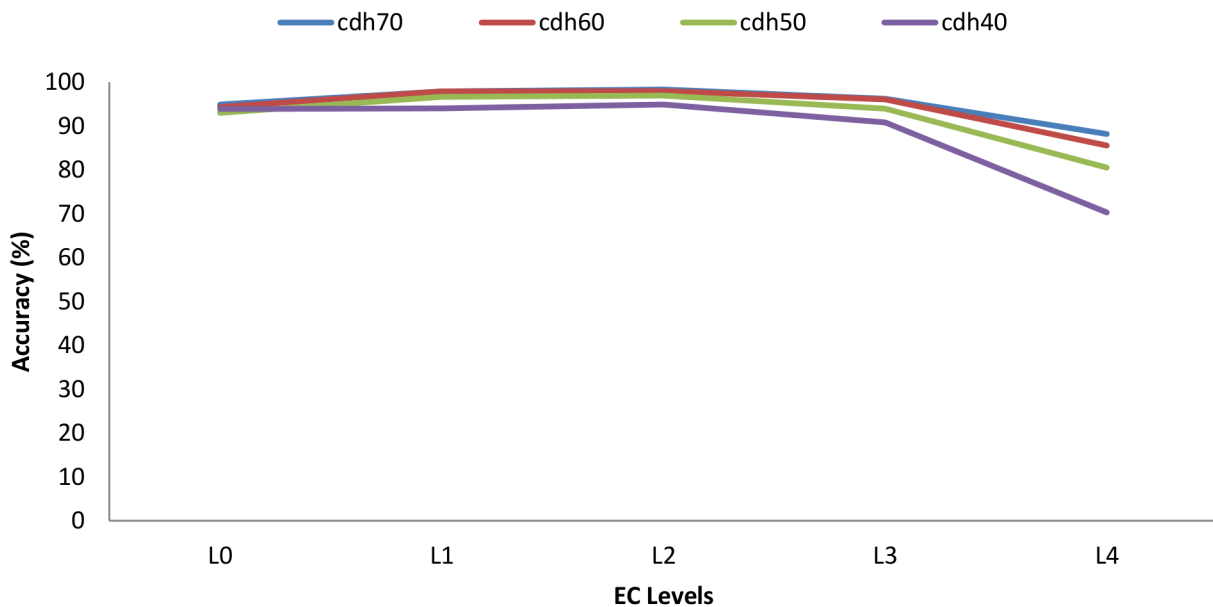

Supplement: Additional file 3 — Figure S2. Accuracy and distribution of enzyme sequence and class. A) Distribution of enzyme sequence and class coverage for cdh70, cdh60, cdh50 and cdh40 datasets. B) Accuracy at each EC level for cdh70, cdh60, cdh50 and cdh40 datasets. [file 1471-2164-16-S7-S16-S3.pdf]

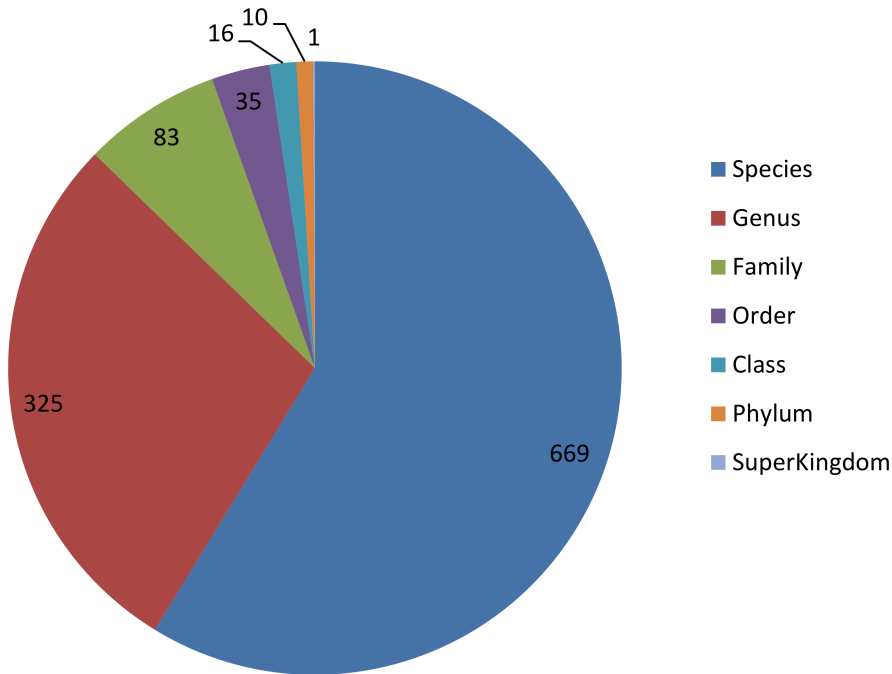

Supplement: Additional file 19 — Figure S5. Taxonomic distribution of bacterial species from metagenomic samples. [file 1471-2164-16-S7-S16-S19.pdf]
