# Supplementary material for: Application of a hierarchical enzyme classification method reveals the role of gut microbiome in human metabolism
Source: BMC Genomics. 2015 Jun 11;16(Suppl 7):S16. doi: 10.1186/1471-2164-16-S7-S16 (PMC4474468; doi:10.1186/1471-2164-16-S7-S16)
Supplement: Additional file 2 — Table S1. Overall predictions of 2 classifiers vs. 3 classifiers. [file 1471-2164-16-S7-S16-S2.pdf]

Table S1. Overall predictions of 2 classifiers vs. 3 classifiers.

EC Level	Predicted by at least 2 of top 3 classifiers	Predicted by all 3 (top) classifiers	% Predicted by all the 3 classifiers
Level-0 (Enzymes)	188866 (62674)	182542 (59890)	96.65 (95.58)
Level-1	62167	61560	99.02
Level-2	61721	61237	99.22
Level-3	60931	60523	99.33
Level-4	60199	59681	99.14
