# Supplementary material for: Application of a hierarchical enzyme classification method reveals the role of gut microbiome in human metabolism
Source: BMC Genomics. 2015 Jun 11;16(Suppl 7):S16. doi: 10.1186/1471-2164-16-S7-S16 (PMC4474468; doi:10.1186/1471-2164-16-S7-S16)
Supplement: Additional file 4 — Table S2. Accuracy of ECemble method using cdh70, cdh60, cdh50 and cdh40 datasets [file 1471-2164-16-S7-S16-S4.pdf]

Table S2. Results showing the accuracy of our method using datasets with sequence identity threshold 70, 60, 50 and 40. Level-0 represents the model to predict if a protein sequence is an enzyme or not. Levels 1-4 represent corresponding levels of the EC number hierarchy.

***Correctly predicted**: Instances predicted by at least 2 of top 3 classifiers

EC Level CDH70	Number of sequences	Correctly Predicted*	% Overall accuracy	Classes	Model(s)
Level-0	193240	188866	97.74	2	1
(Enzymes)	(64948)	(62674)	(96.50)		
Level-1	62674	62167	99.19	6	1
Level-2	62167	61721	99.28	51	6
Level-3	61721	60931	98.72	169	51
Level-4	60931	60199	98.80	1921	169

CDH 60 EC Level	Number of sequences	Correctly Predicted*	% Overall accuracy	Classes	Model(s)
Level-0	15198	148181	97.50	2	1
(Enzymes)	46259	44246	(95.65)		
Level-1	44246	43875	99.17	6	1
Level-2	43875	43539	99.24	51	6
Level-3	43539	42947	98.64	163	51
Level-4	42947	42230	98.33	1808	163

CDH 50 EC Level	Number of sequences	Correctly Predicted*	% Overall accuracy	Classes	Model(s)
Level-0	115350	112115	97.20	2	1
(Enzymes)	(29984)	(28198)	(94.05)		
Level-1	28198	27900	98.95	6	1
Level-2	27900	27647	99.10	51	6
Level-3	27647	27191	98.35	156	51
Level-4	27191	26665	98.07	1656	156

CDH 40 EC Level	Number of sequences	Correctly Predicted*	% Overall accuracy	Classes	Model(s)
Level-0	85944	84071	97.83	2	1
(Enzymes)	(17998)	(16936)	(94.10)		
Level-1	16936	16686	98.53	6	1
Level-2	16686	16477	98.75	51	6
Level-3	16477	15989	97.04	142	51
Level-4	15989	15546	97.23	1453	142
