# Supplementary material for: Application of a hierarchical enzyme classification method reveals the role of gut microbiome in human metabolism
Source: BMC Genomics. 2015 Jun 11;16(Suppl 7):S16. doi: 10.1186/1471-2164-16-S7-S16 (PMC4474468; doi:10.1186/1471-2164-16-S7-S16)
Supplement: Additional file 8 — Figure S3. GMC pathways that validate the role of gut bacteria in human metabolism [file 1471-2164-16-S7-S16-S8.pdf]

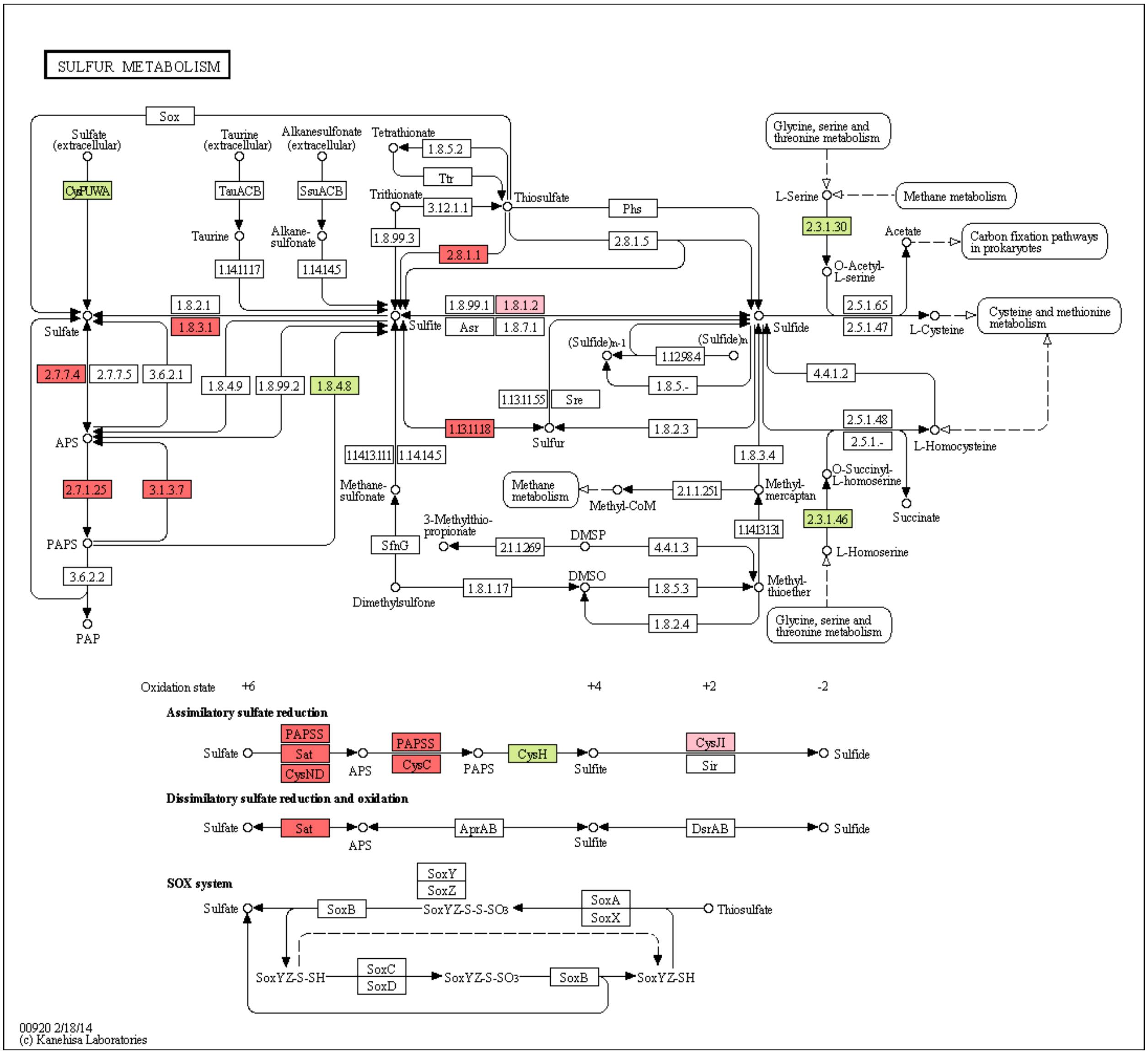
SULFUR METABOLISM
Sox
Sulfate (extracellular)
Taurine (extracellular)
Alkanesulfonate (extracellular)
Tetrathionate
CysPUWA
TauACB
SsuACB
1.8.5.2
Ttr
Trithionate
3.12.1.1
Thiosulfate
Phs
Taurine
Alkane-sulfonate
1.8.99.3
2.8.1.1
2.8.1.5
1.14.11.17
1.14.14.5
1.8.2.1
1.8.3.1
1.8.99.1
1.8.1.2
Sulfite
Asr
1.8.7.1
Sulfide
Sulfate
2.7.7.4
2.7.7.5
3.6.2.1
1.8.4.9
1.8.99.2
1.8.4.8
APS
2.7.1.25
3.1.3.7
PAPS
3.6.2.2
PAP
(Sulfide)n-1
(Sulfide)n
1.12.98.4
1.8.5.-
1.13.11.55
Sre
1.13.11.18
Sulfur
1.8.2.3
1.14.13.111
1.14.14.5
Methane-sulfonate
Methane metabolism
Methyl-CoM
2.1.1.251
1.8.3.4
Methyl-mercaptan
3-Methylthio-propionate
SfnG
2.1.1.269
DMSP
4.4.1.3
1.14.13.131
Dimethylsulfone
1.8.1.17
DMSO
1.8.5.3
1.8.2.4
Methyl-thioether
Glycine, serine and threonine metabolism
L-Serine
Methane metabolism
2.3.1.30
Acetate
Carbon fixation pathways in prokaryotes
O-Acetyl-L-serine
2.5.1.65
2.5.1.47
L-Cysteine
Cysteine and methionine metabolism
4.4.1.2
2.5.1.48
2.5.1.-
L-Homocysteine
O-Succinyl-L-homoserine
2.3.1.46
Succinate
L-Homoserine
Glycine, serine and threonine metabolism
Oxidation state
+6
+4
+2
-2
Assimilatory sulfate reduction
Sulfate
PAPSS
Sat
CysND
APS
PAPSS
CysC
PAPS
CysH
Sulfite
CysJI
Sir
Sulfide
Dissimilatory sulfate reduction and oxidation
Sulfate
Sat
APS
AprAB
Sulfite
DsrAB
Sulfide
SOX system
Sulfate
SoxB
SoxY
SoxZ
SoxYZ-S-S-SO3
SoxA
SoxX
Thiosulfate
SoxYZ-S-SH
SoxC
SoxD
SoxYZ-S-SO3
SoxB
SoxYZ-SH
00920 2/18/14
(c) Kanehisa Laboratories

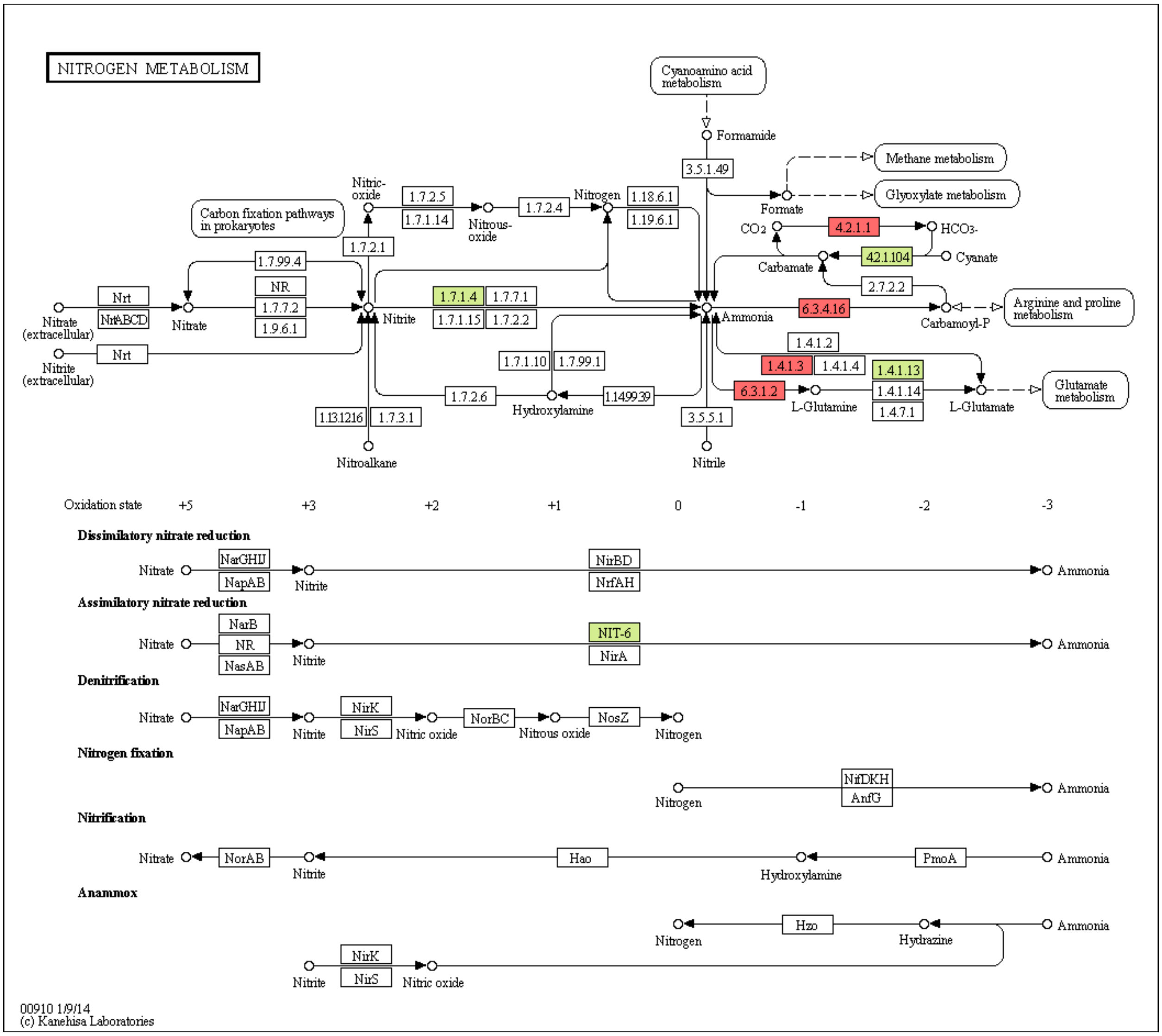
NITROGEN METABOLISM
Cyanoamino acid metabolism
Formamide
3.5.1.49
Methane metabolism
Glyoxylate metabolism
Formate
CO2
4.2.1.1
HCO3-
4.2.1.104
Cyanate
Carbamate
2.7.2.2
Carbon fixation pathways in prokaryotes
Nitric-oxide
1.7.2.5
1.7.1.14
Nitrous-oxide
1.7.2.4
Nitrogen
1.18.6.1
1.19.6.1
1.7.2.1
1.7.99.4
NR
1.7.7.2
1.9.6.1
Nrt
NrtABCD
Nitrate (extracellular)
Nitrate
Nitrite (extracellular)
Nrt
Nitrite
1.7.1.4
1.7.7.1
1.7.1.15
1.7.2.2
Ammonia
6.3.4.16
Carbamoyl-P
Arginine and proline metabolism
1.4.1.2
1.4.1.3
1.4.1.4
1.4.1.13
6.3.1.2
L-Glutamine
1.4.1.14
1.4.7.1
L-Glutamate
Glutamate metabolism
1.7.1.10
1.7.99.1
1.7.2.6
Hydroxylamine
1.14.99.39
1.13.12.16
1.7.3.1
Nitroalkane
3.5.5.1
Nitrile
Oxidation state +5 +3 +2 +1 0 -1 -2 -3
Dissimilatory nitrate reduction
Nitrate NarGHIJ NapAB Nitrite NirBD NrfAH Ammonia
Assimilatory nitrate reduction
Nitrate NarB NR NasAB Nitrite NIT-6 NirA Ammonia
Denitrification
Nitrate NarGHIJ NapAB Nitrite NirK NirS Nitric oxide NorBC Nitrous oxide NosZ Nitrogen
Nitrogen fixation
Nitrogen NifDKH AnfG Ammonia
Nitrification
Nitrate NorAB Nitrite Hao Hydroxylamine PmoA Ammonia
Anammox
Nitrogen Hzo Hydrazine Ammonia
Nitrite NirK NirS Nitric oxide
00910 1/9/14
(c) Kanehisa Laboratories

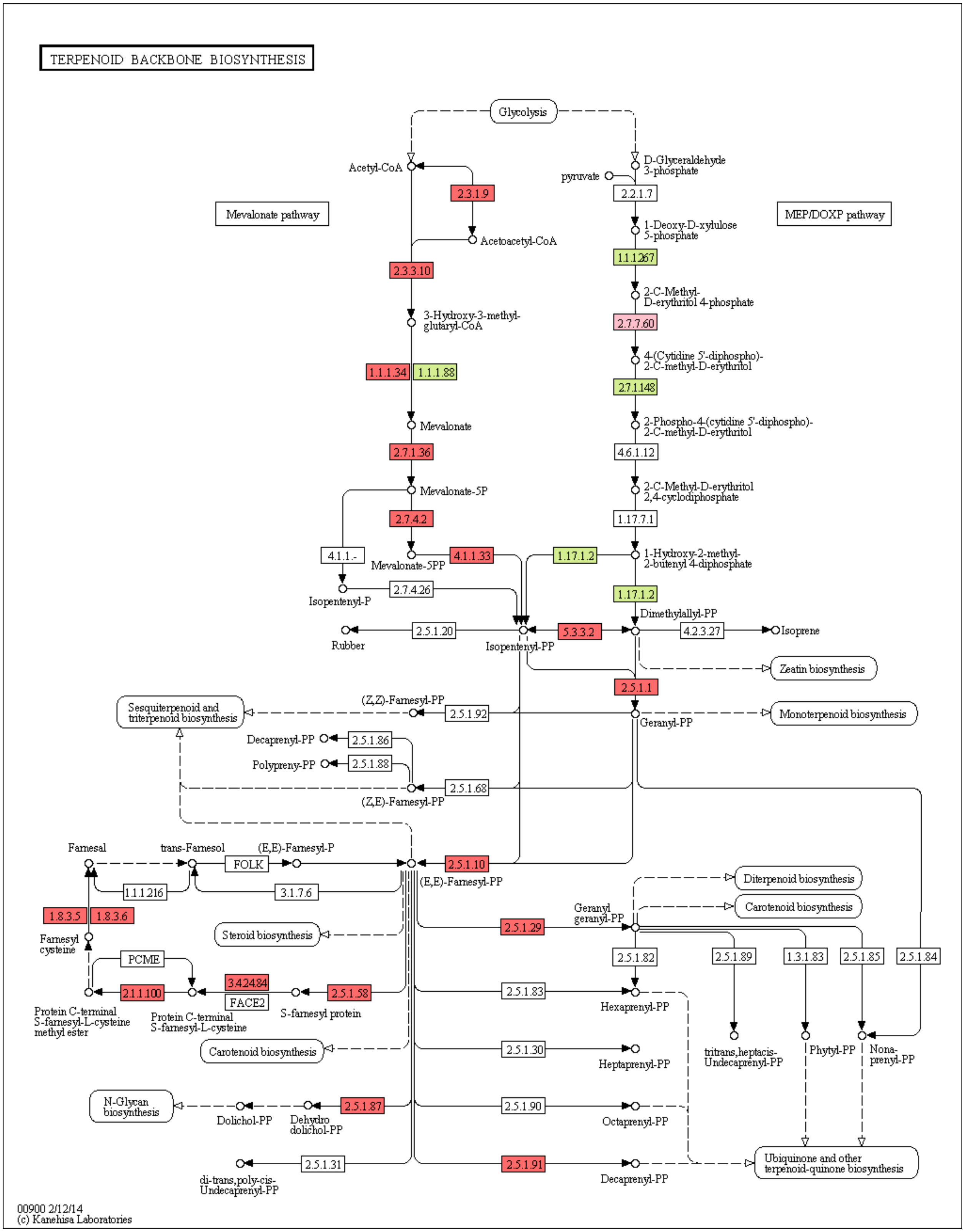
TERPENOID BACKBONE BIOSYNTHESIS
Glycolysis
Acetyl-CoA
D-Glyceraldehyde 3-phosphate
pyruvate
2.3.1.9
2.2.1.7
Mevalonate pathway
MEP/DOXP pathway
Acetoacetyl-CoA
1-Deoxy-D-xylulose 5-phosphate
2.3.3.10
1.1.1.267
2-C-Methyl-D-erythritol 4-phosphate
3-Hydroxy-3-methyl-glutaryl-CoA
2.7.7.60
4-(Cytidine 5'-diphospho)-2-C-methyl-D-erythritol
1.1.1.34
1.1.1.88
2.7.1.148
Mevalonate
2-Phospho-4-(cytidine 5'-diphospho)-2-C-methyl-D-erythritol
2.7.1.36
4.6.1.12
Mevalonate-5P
2-C-Methyl-D-erythritol 2,4-cyclodiphosphate
2.7.4.2
1.17.7.1
4.1.1.-
Mevalonate-5PP
4.1.1.33
1.17.1.2
1-Hydroxy-2-methyl-2-butenyl 4-diphosphate
Isopentenyl-P
2.7.4.26
1.17.1.2
Dimethylallyl-PP
Rubber
2.5.1.20
Isopentenyl-PP
5.3.3.2
4.2.3.27
Isoprene
Zeatin biosynthesis
2.5.1.1
Sesquiterpenoid and triterpenoid biosynthesis
(Z,Z)-Farnesyl-PP
2.5.1.92
Geranyl-PP
Monoterpenoid biosynthesis
Decaprenyl-PP
2.5.1.86
Polypreny-PP
2.5.1.88
2.5.1.68
(Z,E)-Farnesyl-PP
Farnesal
trans-Farnesol
(E,E)-Farnesyl-P
FOLK
2.5.1.10
(E,E)-Farnesyl-PP
1.1.1.216
3.1.7.6
Diterpenoid biosynthesis
Geranyl geranyl-PP
Carotenoid biosynthesis
1.8.3.5
1.8.3.6
Farnesyl cysteine
Steroid biosynthesis
2.5.1.29
PCME
2.5.1.82
2.5.1.89
1.3.1.83
2.5.1.85
2.5.1.84
3.4.24.84
FACE2
2.1.1.100
2.5.1.58
2.5.1.83
Protein C-terminal S-farnesyl-L-cysteine methyl ester
Protein C-terminal S-farnesyl-L-cysteine
S-farnesyl protein
Hexaprenyl-PP
tritrans,heptacis-Undecaprenyl-PP
Phytyl-PP
Nona-prenyl-PP
Carotenoid biosynthesis
2.5.1.30
Heptaprenyl-PP
N-Glycan biosynthesis
Dolichol-PP
Dehydro dolichol-PP
2.5.1.87
2.5.1.90
Octaprenyl-PP
Ubiquinone and other terpenoid-quinone biosynthesis
2.5.1.31
di-trans,poly-cis-Undecaprenyl-PP
2.5.1.91
Decaprenyl-PP
00900 2/12/14
(c) Kanehisa Laboratories

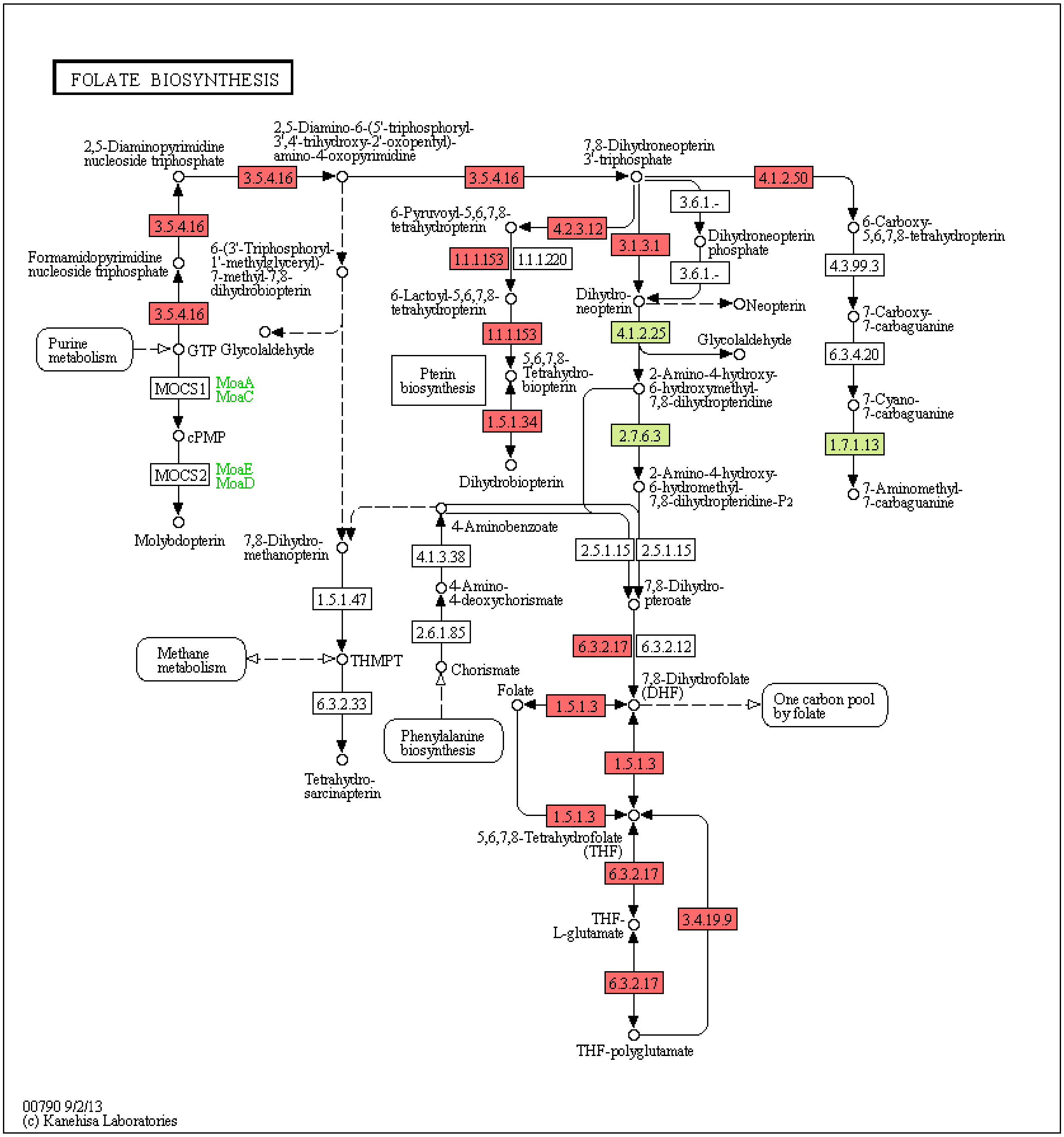
FOLATE BIOSYNTHESIS
2,5-Diaminopyrimidine nucleoside triphosphate
3.5.4.16
2,5-Diamino-6-(5'-triphosphoryl-3',4'-trihydroxy-2'-oxopentyl)-amino-4-oxopyrimidine
7,8-Dihydroneopterin 3'-triphosphate
4.1.2.50
6-Carboxy-5,6,7,8-tetrahydropterin
4.3.99.3
7-Carboxy-7-carbaguanine
6.3.4.20
7-Cyano-7-carbaguanine
1.7.1.13
7-Aminomethyl-7-carbaguanine
3.6.1.-
Dihydroneopterin phosphate
6-Pyruvoyl-5,6,7,8-tetrahydropterin
4.2.3.12
3.1.3.1
1.1.1.153
1.1.1.220
6-Lactoyl-5,6,7,8-tetrahydropterin
5,6,7,8-Tetrahydro-biopterin
1.5.1.34
Dihydrobiopterin
Pterin biosynthesis
Dihydro-neopterin
Neopterin
4.1.2.25
Glycolaldehyde
2-Amino-4-hydroxy-6-hydroxymethyl-7,8-dihydropteridine
2.7.6.3
2-Amino-4-hydroxy-6-hydromethyl-7,8-dihydropteridine-P2
Formamidopyrimidine nucleoside triphosphate
6-(3'-Triphosphoryl-1'-methylglyceryl)-7-methyl-7,8-dihydrobiopterin
Purine metabolism
GTP
MOCS1
MoaA
MoaC
cPMP
MOCS2
MoaE
MoaD
Molybdopterin
7,8-Dihydro-methanopterin
1.5.1.47
Methane metabolism
THMPT
6.3.2.33
Tetrahydro-sarcinapterin
4-Aminobenzoate
4.1.3.38
4-Amino-4-deoxychorismate
2.6.1.85
Chorismate
Phenylalanine biosynthesis
2.5.1.15
7,8-Dihydro-pteroate
6.3.2.17
6.3.2.12
7,8-Dihydrofolate (DHF)
Folate
1.5.1.3
One carbon pool by folate
5,6,7,8-Tetrahydrofolate (THF)
THF-L-glutamate
3.4.19.9
THF-polyglutamate
00790 9/2/13
(c) Kanehisa Laboratories

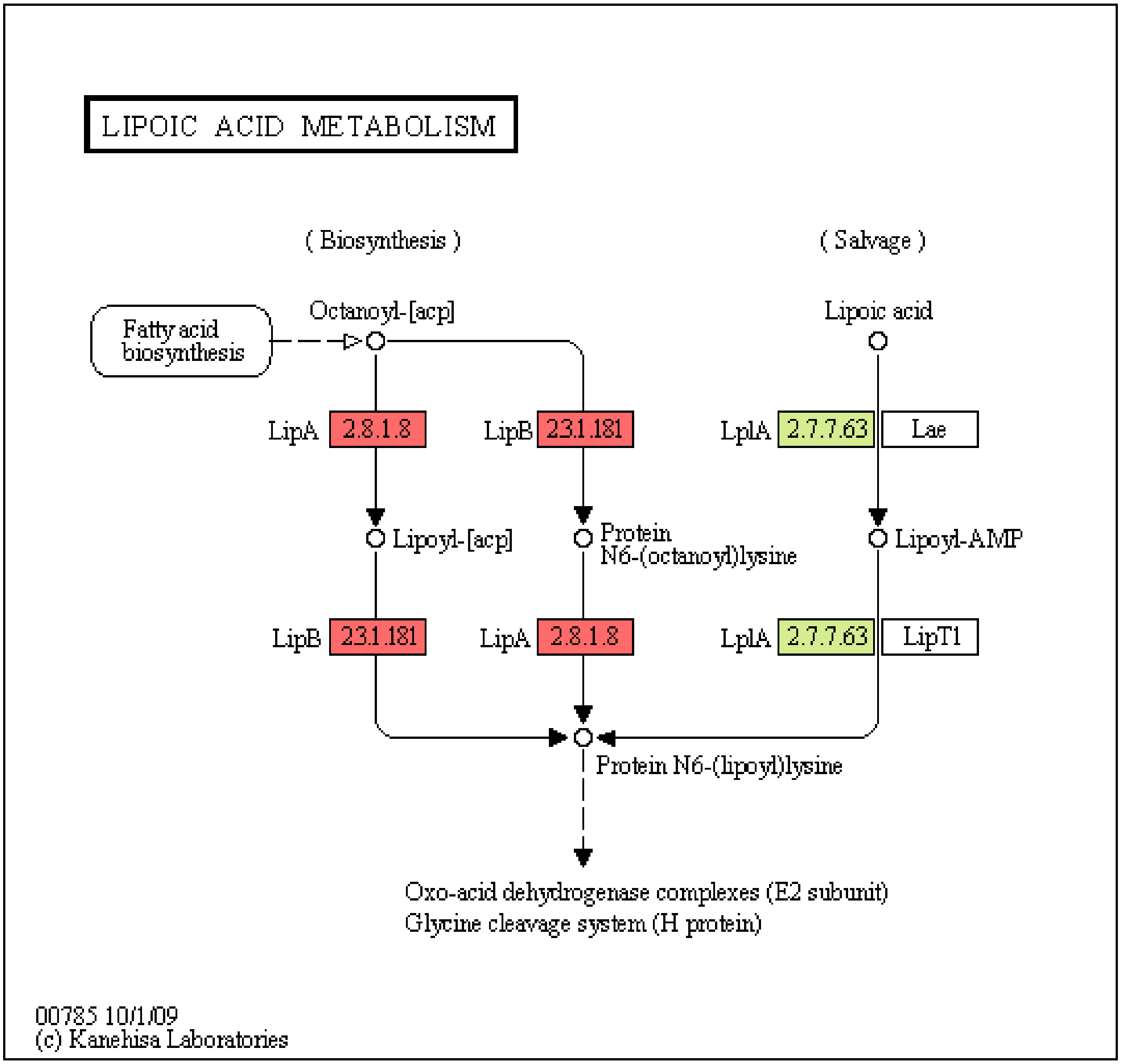

LIPOIC ACID METABOLISM
(Biosynthesis)
(Salvage)
Fatty acid biosynthesis
Octanoyl-[acp]
Lipoic acid
LipA
2.8.1.8
LipB
2.3.1.181
LplA
2.7.7.63
Lae
Lipoyl-[acp]
Protein N6-(octanoyl)lysine
Lipoyl-AMP
LipB
2.3.1.181
LipA
2.8.1.8
LplA
2.7.7.63
LipT1
Protein N6-(lipoyl)lysine
Oxo-acid dehydrogenase complexes (E2 subunit)
Glycine cleavage system (H protein)
00785 10/1/09
(c) Kanehisa Laboratories

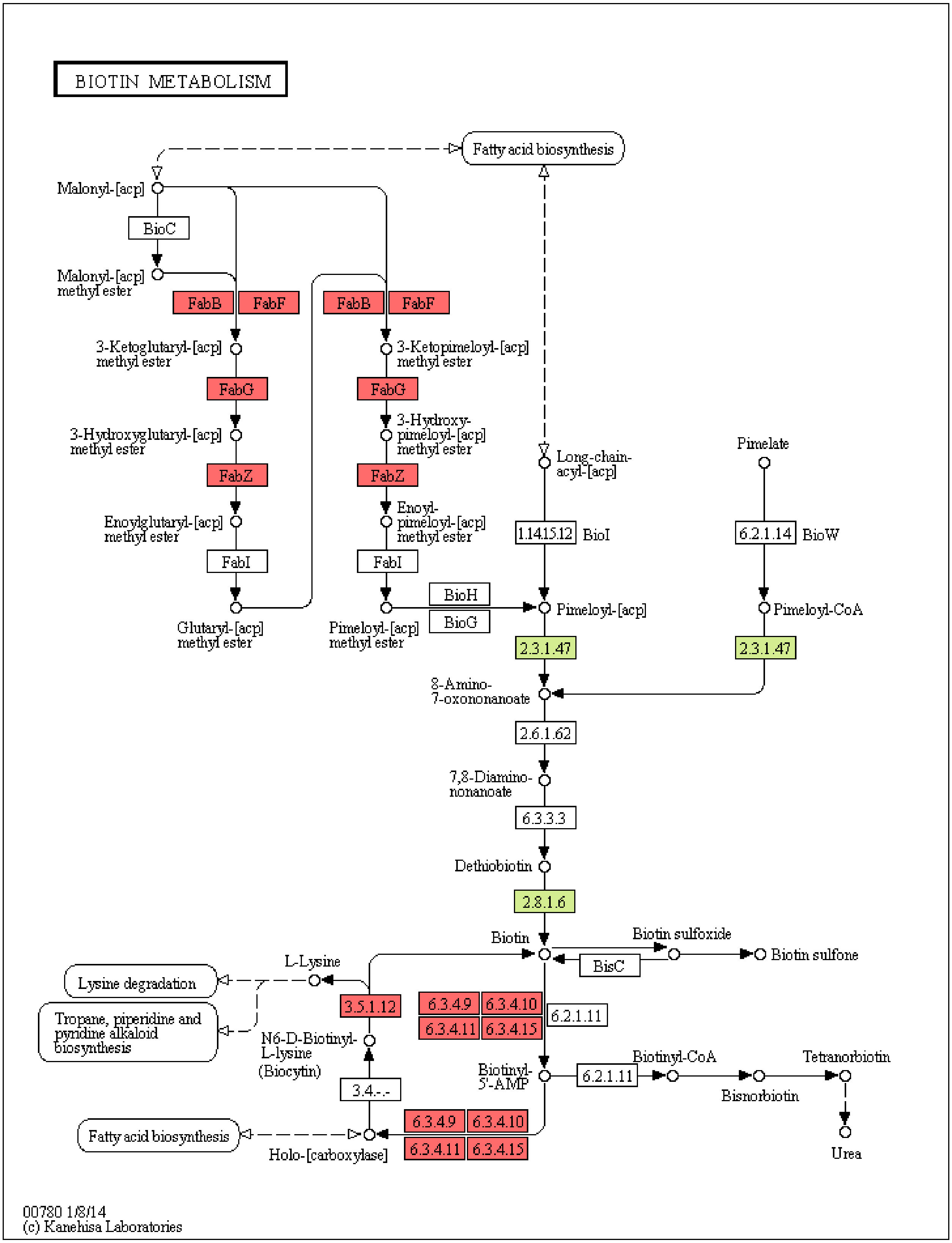
BIOTIN METABOLISM
Fatty acid biosynthesis
Malonyl-[acp]
BioC
Malonyl-[acp] methyl ester
FabB
FabF
FabB
FabF
3-Ketoglutaryl-[acp] methyl ester
3-Ketopimeloyl-[acp] methyl ester
FabG
FabG
3-Hydroxyglutaryl-[acp] methyl ester
3-Hydroxy-pimeloyl-[acp] methyl ester
FabZ
FabZ
Enoylglutaryl-[acp] methyl ester
Enoyl-pimeloyl-[acp] methyl ester
FabI
FabI
Glutaryl-[acp] methyl ester
Pimeloyl-[acp] methyl ester
BioH
BioG
Long-chain-acyl-[acp]
1.14.15.12
BioI
Pimelate
6.2.1.14
BioW
Pimeloyl-[acp]
Pimeloyl-CoA
2.3.1.47
2.3.1.47
8-Amino-7-oxononanoate
2.6.1.62
7,8-Diamino-nonanoate
6.3.3.3
Dethiobiotin
2.8.1.6
Biotin
Biotin sulfoxide
Biotin sulfone
BisC
L-Lysine
Lysine degradation
Tropane, piperidine and pyridine alkaloid biosynthesis
3.5.1.12
6.3.4.9
6.3.4.10
6.3.4.11
6.3.4.15
6.2.1.11
N6-D-Biotinyl-L-lysine (Biocytin)
3.4.-.-
Biotinyl-5'-AMP
6.2.1.11
Biotinyl-CoA
Bisnorbiotin
Tetranorbiotin
Urea
Fatty acid biosynthesis
6.3.4.9
6.3.4.10
6.3.4.11
6.3.4.15
Holo-[carboxylase]
00780 1/8/14
(c) Kanehisa Laboratories

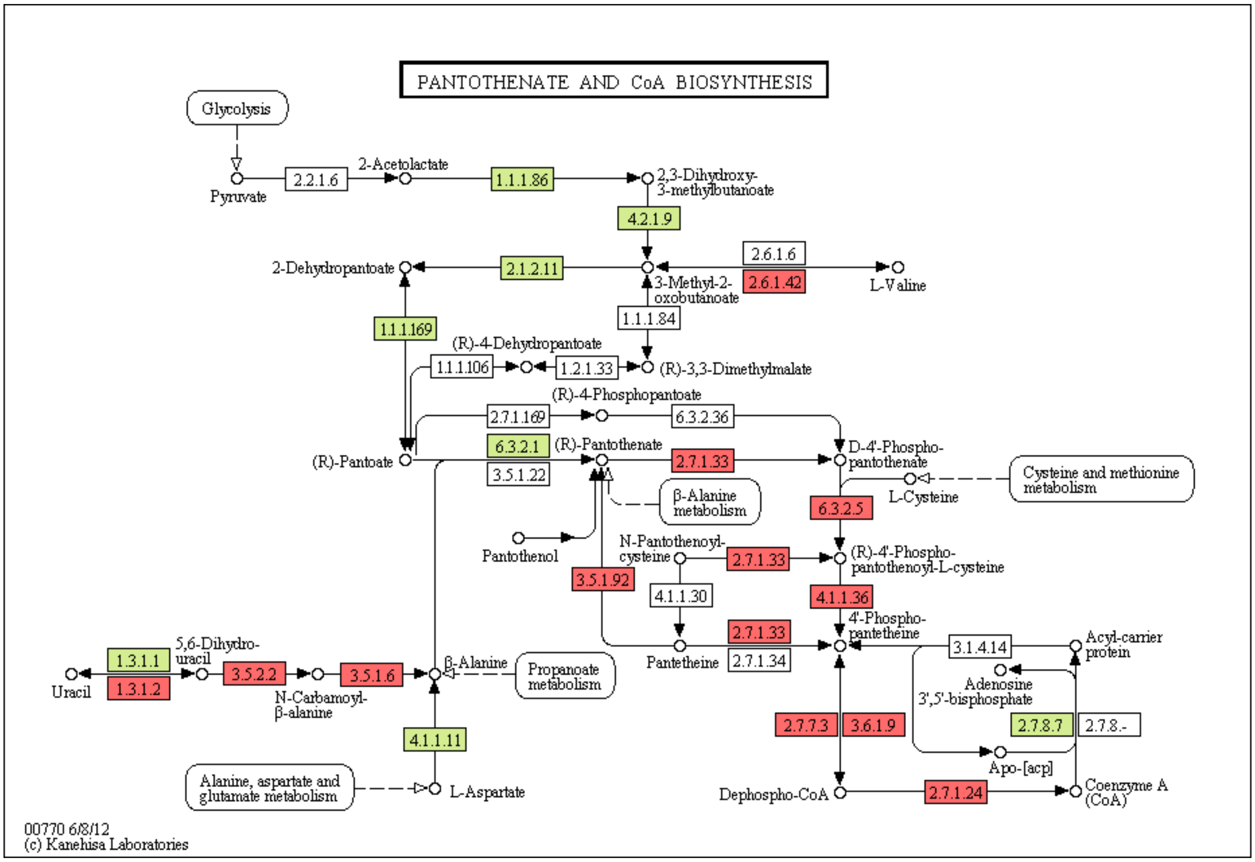
PANTOTHENATE AND CoA BIOSYNTHESIS
Glycolysis
Pyruvate
2.2.1.6
2-Acetolactate
1.1.1.86
2,3-Dihydroxy-
3-methylbutanoate
4.2.1.9
2-Dehydropantoate
2.1.2.11
2.6.1.6
2.6.1.42
L-Valine
3-Methyl-2-
oxobutanoate
1.1.1.84
1.1.1.169
(R)-4-Dehydropantoate
1.1.1.106
1.2.1.33
(R)-3,3-Dimethylmalate
(R)-4-Phosphopantoate
2.7.1.169
6.3.2.36
(R)-Pantoate
6.3.2.1
3.5.1.22
(R)-Pantothenate
2.7.1.33
D-4'-Phospho-
pantothenate
Cysteine and methionine
metabolism
L-Cysteine
β-Alanine
metabolism
6.3.2.5
Pantothenol
N-Pantothenoyl-
cysteine
2.7.1.33
(R)-4'-Phospho-
pantothenoyl-L-cysteine
3.5.1.92
4.1.1.30
4.1.1.36
4'-Phospho-
pantetheine
2.7.1.33
Pantetheine
2.7.1.34
3.1.4.14
Acyl-carrier
protein
Adenosine
3',5'-bisphosphate
1.3.1.1
5,6-Dihydro-
uracil
Uracil
1.3.1.2
3.5.2.2
3.5.1.6
N-Carbamoyl-
β-alanine
β-Alanine
Propanoate
metabolism
2.7.7.3
3.6.1.9
2.7.8.7
2.7.8.-
4.1.1.11
Apo-[acp]
Alanine, aspartate and
glutamate metabolism
L-Aspartate
Dephospho-CoA
2.7.1.24
Coenzyme A
(CoA)
00770 6/8/12
(c) Kanehisa Laboratories

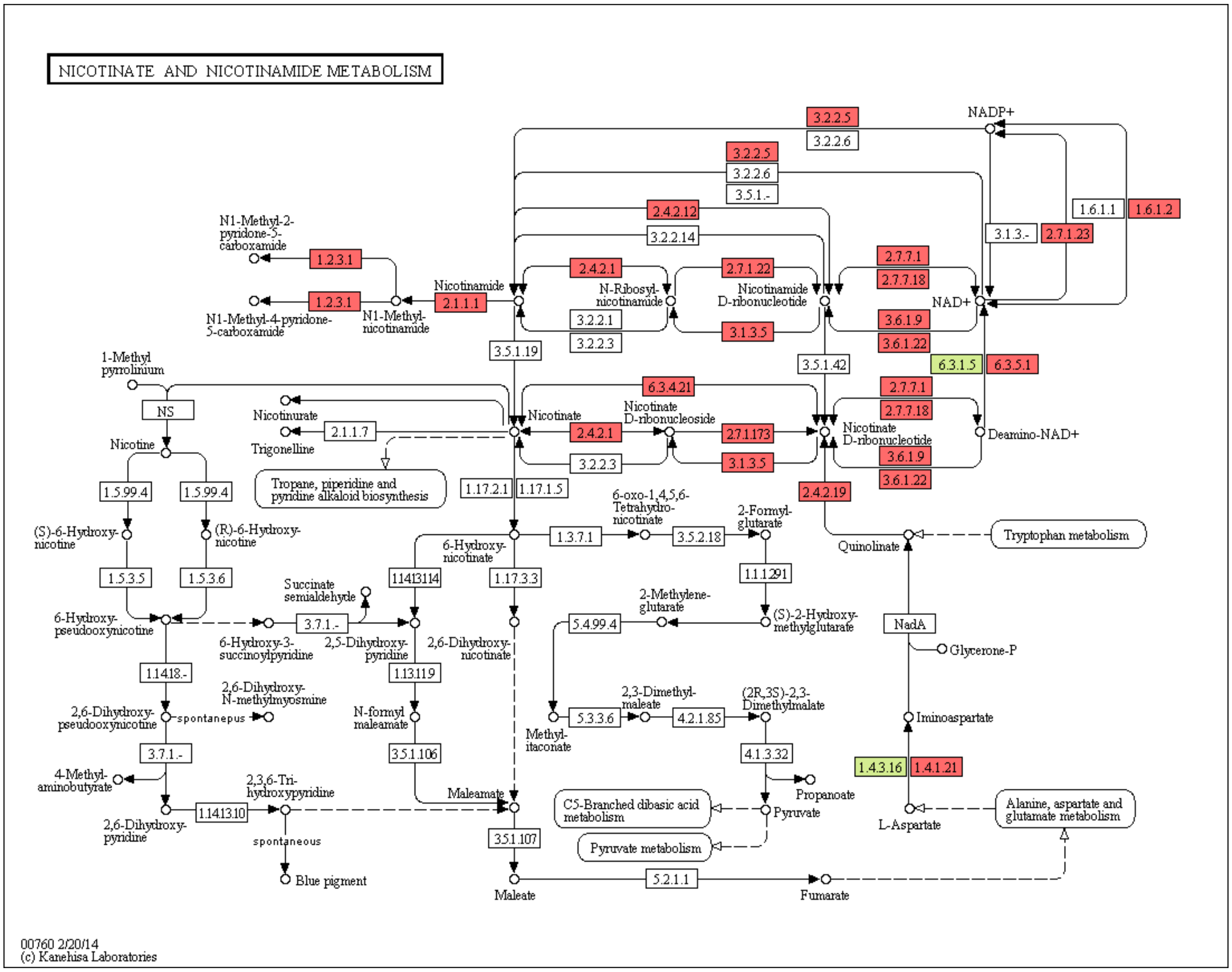
NICOTINATE AND NICOTINAMIDE METABOLISM
NADP+
3.2.2.5
3.2.2.6
3.5.1.-
2.4.2.12
3.2.2.14
1.6.1.1
1.6.1.2
3.1.3.-
2.7.1.23
N1-Methyl-2-pyridone-5-carboxamide
1.2.3.1
N1-Methyl-4-pyridone-5-carboxamide
N1-Methyl-nicotinamide
Nicotinamide
2.1.1.1
2.4.2.1
N-Ribosyl-nicotinamide
3.2.2.1
3.2.2.3
2.7.1.22
Nicotinamide D-ribonucleotide
3.1.3.5
2.7.7.1
2.7.7.18
NAD+
3.6.1.9
3.6.1.22
6.3.1.5
6.3.5.1
3.5.1.19
3.5.1.42
1-Methyl pyrrolinium
NS
Nicotine
Nicotinurate
2.1.1.7
Trigonelline
Nicotinate
6.3.4.21
Nicotinate D-ribonucleoside
2.7.1.173
Nicotinate D-ribonucleotide
Deamino-NAD+
Tropane, piperidine and pyridine alkaloid biosynthesis
1.5.99.4
1.17.2.1
1.17.1.5
2.4.2.19
(S)-6-Hydroxy-nicotine
(R)-6-Hydroxy-nicotine
6-oxo-1,4,5,6-Tetrahydro-nicotinate
2-Formyl-glutarate
Quinolinate
Tryptophan metabolism
6-Hydroxy-nicotinate
1.3.7.1
3.5.2.18
1.5.3.5
1.5.3.6
1.14.13.114
1.17.3.3
1.1.1.291
Succinate semialdehyde
2-Methylene-glutarate
(S)-2-Hydroxy-methylglutarate
6-Hydroxy-pseudooxynicotine
3.7.1.-
6-Hydroxy-3-succinoylpyridine
2,5-Dihydroxy-pyridine
2,6-Dihydroxy-nicotinate
5.4.99.4
NadA
Glycerone-P
1.14.18.-
1.13.11.9
2,6-Dihydroxy-N-methylmyosmine
2,3-Dimethyl-maleate
(2R,3S)-2,3-Dimethylmalate
2,6-Dihydroxy-pseudooxynicotine
spontaneous
N-formyl maleamate
5.3.3.6
4.2.1.85
Iminoaspartate
Methyl-itaconate
4-Methyl-aminobutyrate
3.5.1.106
4.1.3.32
1.4.3.16
1.4.1.21
2,3,6-Tri-hydroxypyridine
Propanoate
Maleamate
C5-Branched dibasic acid metabolism
Pyruvate
L-Aspartate
Alanine, aspartate and glutamate metabolism
2,6-Dihydroxy-pyridine
1.14.13.10
3.5.1.107
Pyruvate metabolism
Blue pigment
5.2.1.1
Maleate
Fumarate
00760 2/20/14
(c) Kanehisa Laboratories

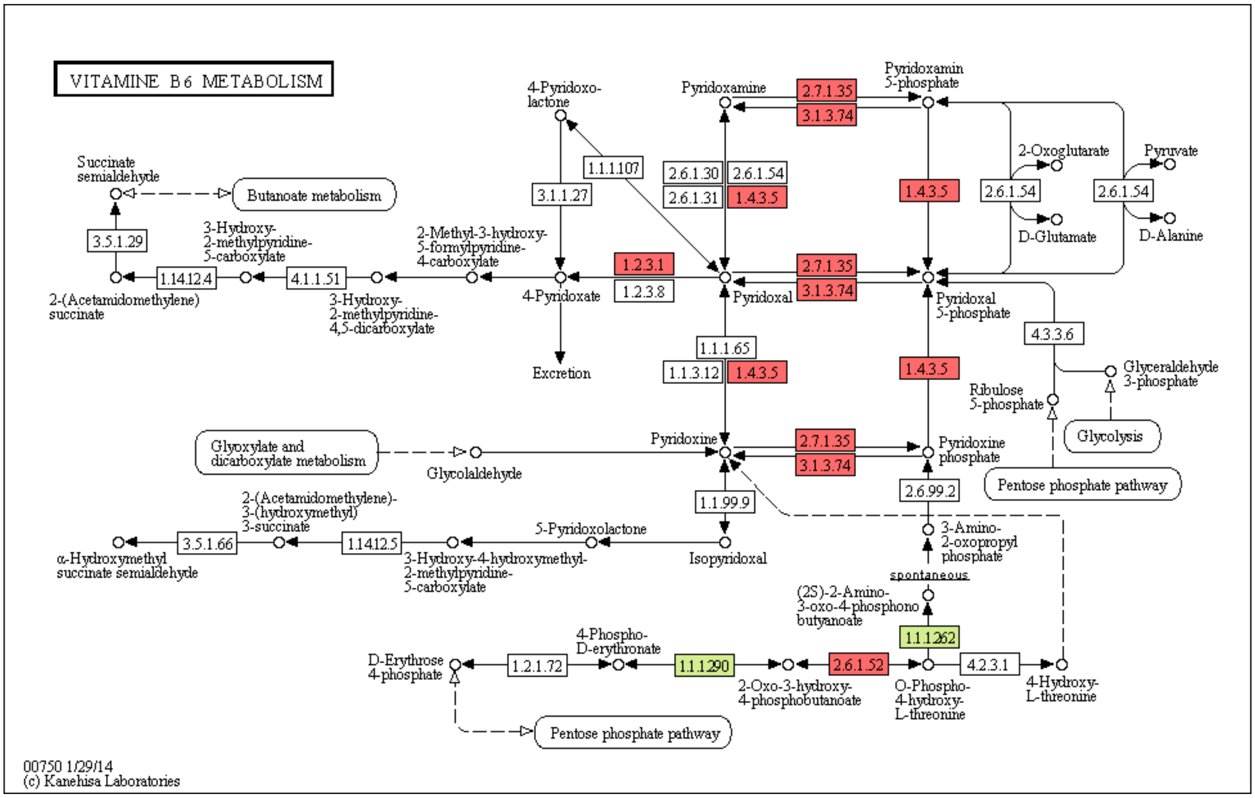
VITAMINE B6 METABOLISM
4-Pyridoxo-lactone
Pyridoxamine
2.7.1.35
3.1.3.74
Pyridoxamin 5-phosphate
Succinate semialdehyde
Butanoate metabolism
1.1.1.107
3.1.1.27
2.6.1.30
2.6.1.31
2.6.1.54
1.4.3.5
2-Oxoglutarate
Pyruvate
2.6.1.54
D-Glutamate
D-Alanine
3.5.1.29
3-Hydroxy-2-methylpyridine-5-carboxylate
2-Methyl-3-hydroxy-5-formylpyridine-4-carboxylate
1.2.1.1
1.2.3.8
2-(Acetamidomethylene) succinate
1.14.12.4
4.1.1.51
3-Hydroxy-2-methylpyridine-4,5-dicarboxylate
4-Pyridoxate
Pyridoxal
Pyridoxal 5-phosphate
4.3.3.6
1.1.1.65
1.1.3.12
Excretion
Glyceraldehyde 3-phosphate
Ribulose 5-phosphate
Glycolysis
Glyoxylate and dicarboxylate metabolism
Glycolaldehyde
Pyridoxine
Pyridoxine phosphate
Pentose phosphate pathway
2.6.99.2
1.1.99.9
2-(Acetamidomethylene)-3-(hydroxymethyl)-3-succinate
5-Pyridoxolactone
α-Hydroxymethyl succinate semialdehyde
3.5.1.66
1.14.12.5
3-Hydroxy-4-hydroxymethyl-2-methylpyridine-5-carboxylate
Isopyridoxal
3-Amino-2-oxopropyl phosphate
spontaneous
(2S)-2-Amino-3-oxo-4-phosphono butanoate
1.1.1.262
4-Phospho-D-erythronate
D-Erythrose 4-phosphate
1.2.1.72
1.1.1.290
2.6.1.52
4.2.1.3
2-Oxo-3-hydroxy-4-phosphobutanoate
O-Phospho-4-hydroxy-L-threonine
4-Hydroxy-L-threonine
Pentose phosphate pathway
00750 1/29/14
(c) Kanehisa Laboratories

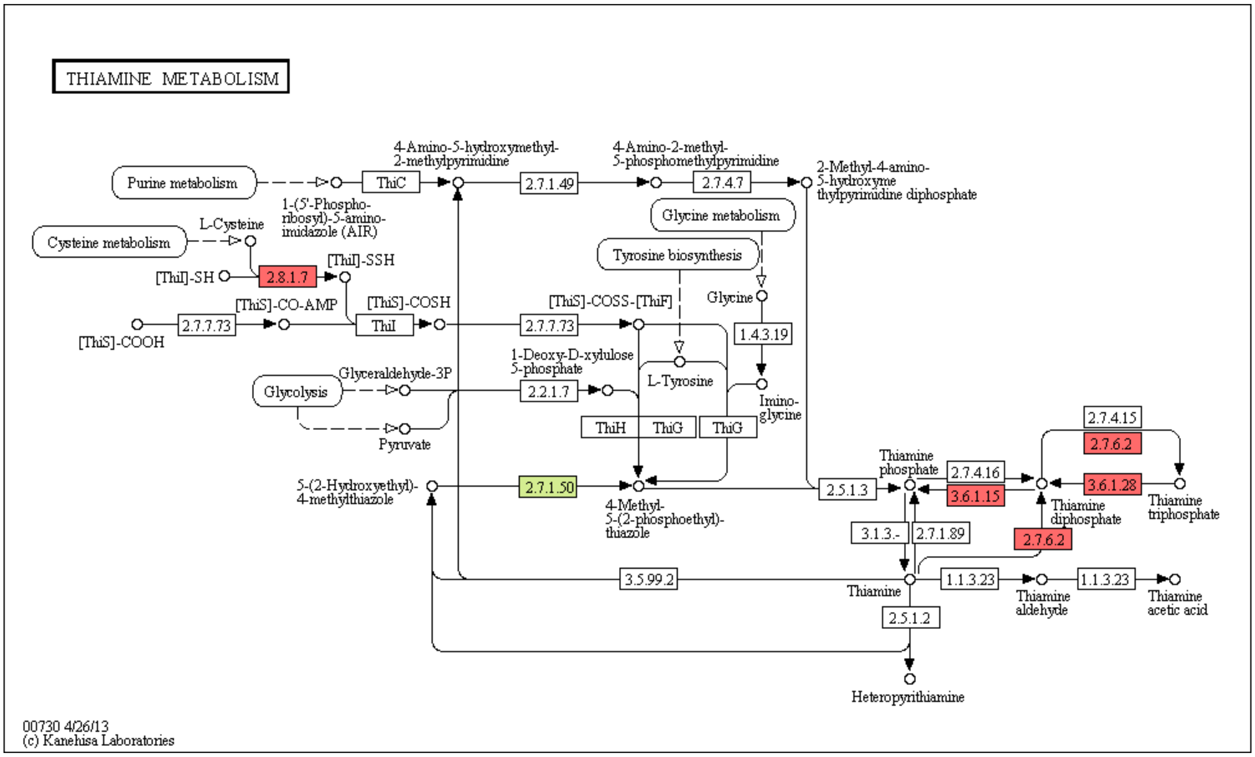

THIAMINE METABOLISM
Purine metabolism
4-Amino-5-hydroxymethyl-2-methylpyrimidine
ThiC
2.7.1.49
4-Amino-2-methyl-5-phosphomethylpyrimidine
2.7.4.7
2-Methyl-4-amino-5-hydroxyme thylpyrimidine diphosphate
1-(5'-Phospho-ribosyl)-5-amino-imidazole (AIR)
Cysteine metabolism
L-Cysteine
Glycine metabolism
Tyrosine biosynthesis
[ThiI]-SH
2.8.1.7
[ThiI]-SSH
Glycine
[ThiS]-CO-AMP
[ThiS]-COSH
[ThiS]-COSS-[ThiF]
1.4.3.19
[ThiS]-COOH
2.7.7.73
ThiI
2.7.7.73
1-Deoxy-D-xylulose 5-phosphate
Glycolysis
Glyceraldehyde-3P
2.2.1.7
L-Tyrosine
Imino-glycine
Pyruvate
ThiH
ThiG
ThiG
2.7.4.15
2.7.6.2
Thiamine phosphate
2.7.4.16
5-(2-Hydroxyethyl)-4-methylthiazole
2.7.1.50
2.5.1.3
3.6.1.15
3.6.1.28
Thiamine diphosphate
Thiamine triphosphate
4-Methyl-5-(2-phosphoethyl)-thiazole
3.1.3.-
2.7.1.89
2.7.6.2
3.5.99.2
Thiamine
1.1.3.23
Thiamine aldehyde
1.1.3.23
Thiamine acetic acid
2.5.1.2
Heteropyrithiamine
00730 4/26/13
(c) Kanehisa Laboratories

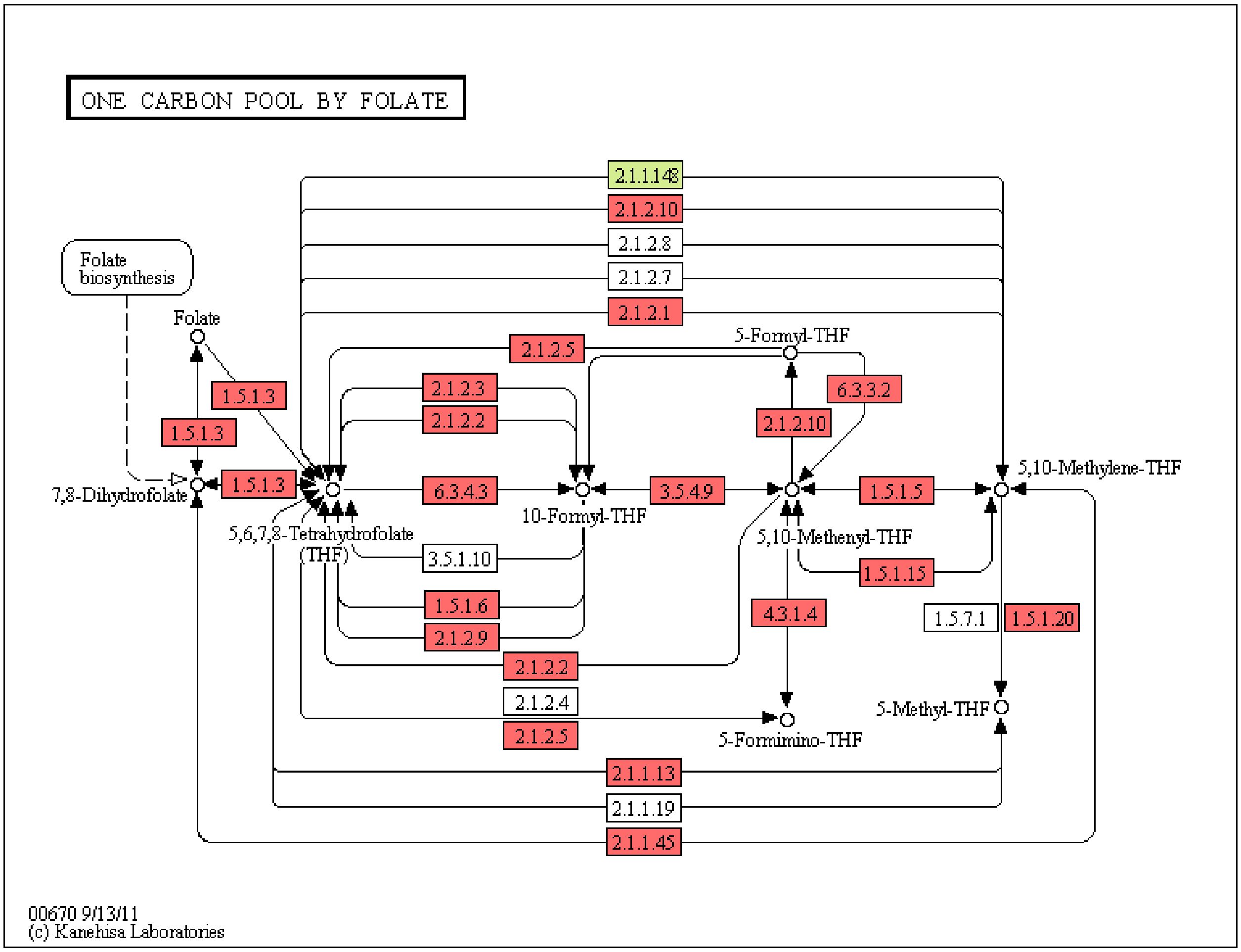
ONE CARBON POOL BY FOLATE
2.1.1.148
2.1.2.10
2.1.2.8
2.1.2.7
2.1.2.1
Folate biosynthesis
Folate
1.5.1.3
1.5.1.3
1.5.1.3
7,8-Dihydrofolate
2.1.2.5
2.1.2.3
2.1.2.2
6.3.4.3
5-Formyl-THF
6.3.3.2
2.1.2.10
3.5.4.9
1.5.1.5
5,10-Methylene-THF
5,6,7,8-Tetrahydrofolate (THF)
10-Formyl-THF
5,10-Methenyl-THF
3.5.1.10
1.5.1.15
1.5.1.6
4.3.1.4
1.5.7.1
1.5.1.20
2.1.2.9
2.1.2.2
2.1.2.4
2.1.2.5
5-Formimino-THF
5-Methyl-THF
2.1.1.13
2.1.1.19
2.1.1.45
00670 9/13/11
(c) Kanehisa Laboratories

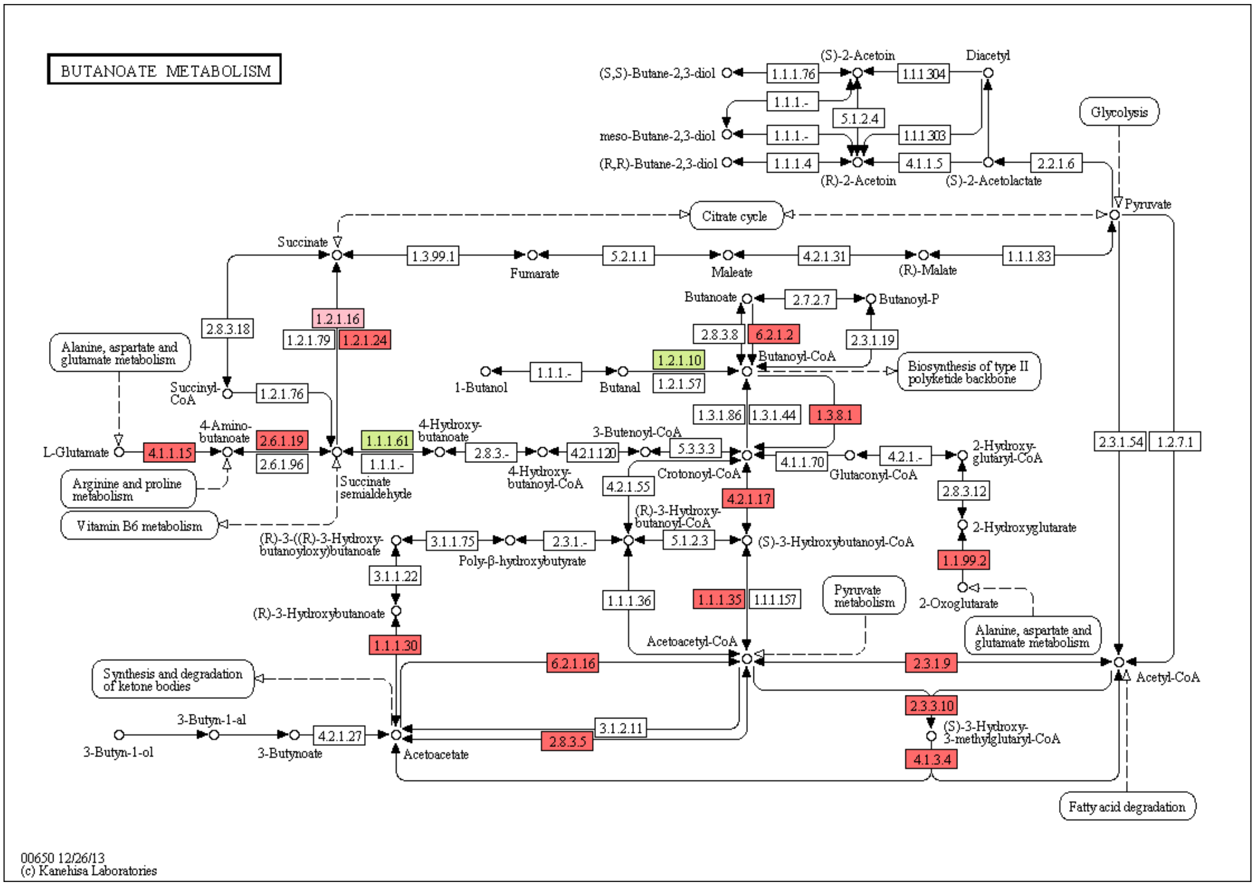
BUTANOATE METABOLISM
(S,S)-Butane-2,3-diol
1.1.1.76
(S)-2-Acetoin
1.1.1.304
Diacetyl
1.1.1.-
5.1.2.4
meso-Butane-2,3-diol
1.1.1.-
1.1.1.303
Glycolysis
(R,R)-Butane-2,3-diol
1.1.1.4
4.1.1.5
2.2.1.6
(R)-2-Acetoin
(S)-2-Acetolactate
Pyruvate
Citrate cycle
Succinate
1.3.99.1
Fumarate
5.2.1.1
Maleate
4.2.1.31
(R)-Malate
1.1.1.83
Butanoate
2.7.2.7
Butanoyl-P
2.8.3.18
1.2.1.16
1.2.1.79
1.2.1.24
2.8.3.8
6.2.1.2
2.3.1.19
Alanine, aspartate and glutamate metabolism
1.2.1.10
Butanoyl-CoA
1.1.1.-
Biosynthesis of type II polyketide backbone
1-Butanol
Butanal
1.2.1.57
Succinyl-CoA
1.2.1.76
1.3.1.86
1.3.1.44
1.3.8.1
2.3.1.54
1.2.7.1
4-Amino-butanoate
4-Hydroxy-butanoate
3-Butenoyl-CoA
L-Glutamate
4.1.1.15
2.6.1.19
1.1.1.61
2.8.3.-
4.2.1.120
5.3.3.3
4.1.1.70
4.2.1.-
2-Hydroxy-glutaryl-CoA
2.6.1.96
1.1.1.-
Arginine and proline metabolism
Succinate semialdehyde
4-Hydroxy-butanoyl-CoA
4.2.1.55
Crotonoyl-CoA
Glutaconyl-CoA
2.8.3.12
4.2.1.17
(R)-3-Hydroxy-butanoyl-CoA
Vitamin B6 metabolism
2-Hydroxyglutarate
(R)-3-((R)-3-Hydroxy-butanoyloxy)butanoate
3.1.1.75
2.3.1.-
5.1.2.3
(S)-3-Hydroxybutanoyl-CoA
Poly-β-hydroxybutyrate
1.1.99.2
3.1.1.22
Pyruvate metabolism
1.1.1.36
1.1.1.35
1.1.1.157
2-Oxoglutarate
(R)-3-Hydroxybutanoate
Alanine, aspartate and glutamate metabolism
Acetoacetyl-CoA
1.1.1.30
6.2.1.16
2.3.1.9
Acetyl-CoA
Synthesis and degradation of ketone bodies
2.3.3.10
3-Butyn-1-al
3.1.2.11
(S)-3-Hydroxy-3-methylglutaryl-CoA
3-Butyn-1-ol
3-Butynoate
4.2.1.27
Acetoacetate
2.8.3.5
4.1.3.4
Fatty acid degradation
00650 12/26/13
(c) Kanehisa Laboratories

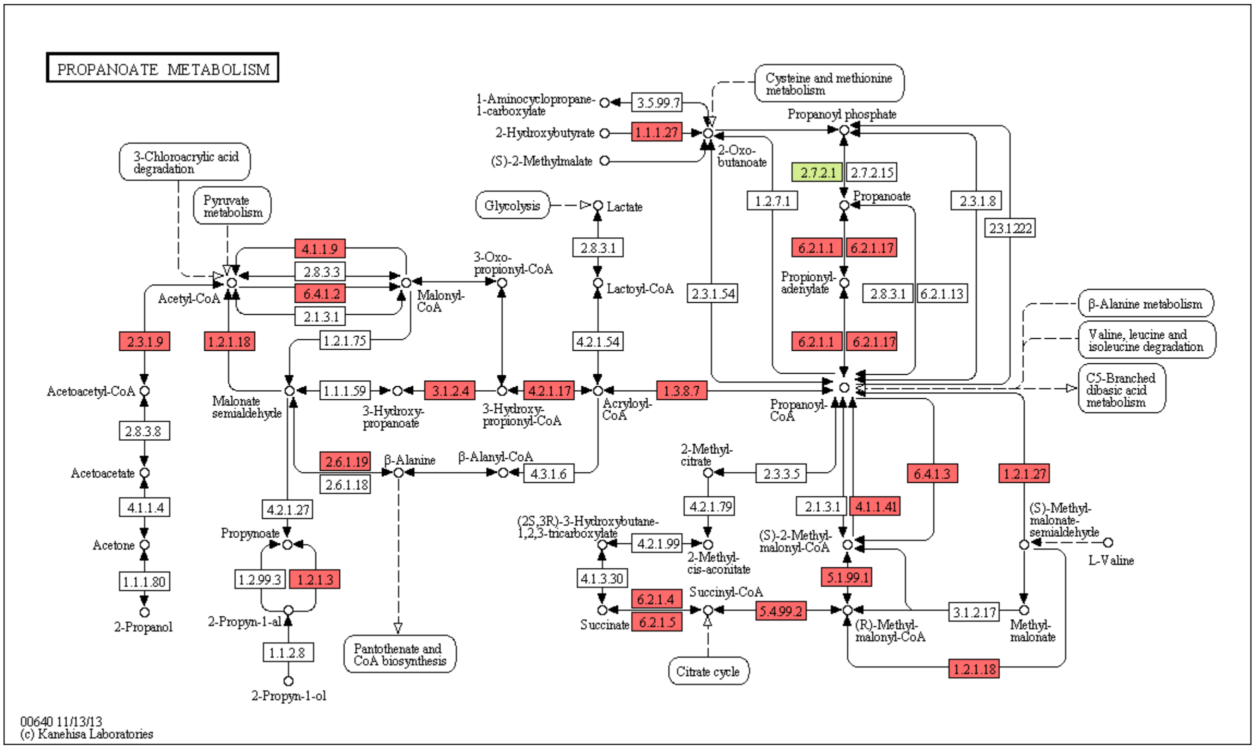

PROPANOATE METABOLISM
1-Aminocyclopropane-1-carboxylate
3.5.99.7
Cysteine and methionine metabolism
2-Hydroxybutyrate
1.1.1.27
2-Oxo-butanoate
Propanoyl phosphate
(S)-2-Methylmalate
3-Chloroacrylic acid degradation
2.7.2.1
2.7.2.15
Pyruvate metabolism
Glycolysis
Lactate
1.2.7.1
Propanoate
2.3.1.8
2.3.1.222
4.1.1.9
2.8.3.1
6.2.1.1
6.2.1.17
2.8.3.3
3-Oxo-propionyl-CoA
Acetyl-CoA
6.4.1.2
Malonyl-CoA
Lactoyl-CoA
2.3.1.54
Propionyl-adenylate
2.8.3.1
6.2.1.13
2.1.3.1
β-Alanine metabolism
2.3.1.9
1.2.1.18
1.2.1.75
4.2.1.54
6.2.1.1
6.2.1.17
Valine, leucine and isoleucine degradation
Acetoacetyl-CoA
1.1.1.59
3.1.2.4
4.2.1.17
1.3.8.7
C5-Branched dibasic acid metabolism
Malonate semialdehyde
3-Hydroxy-propanoate
3-Hydroxy-propionyl-CoA
Acryloyl-CoA
Propanoyl-CoA
2.8.3.8
2.6.1.19
β-Alanine
β-Alanyl-CoA
2-Methyl-citrate
Acetoacetate
2.6.1.18
4.3.1.6
2.3.3.5
6.4.1.3
1.2.1.27
4.1.1.4
4.2.1.27
4.2.1.79
2.1.3.1
4.1.1.41
(S)-Methyl-malonate-semialdehyde
Propynoate
(2S,3R)-3-Hydroxybutane-1,2,3-tricarboxylate
4.2.1.99
(S)-2-Methyl-malonyl-CoA
Acetone
L-Valine
2-Methyl-cis-aconitate
1.1.1.80
1.2.99.3
1.2.1.3
4.1.3.30
5.1.99.1
6.2.1.4
Succinyl-CoA
2-Propanol
2-Propyn-1-al
Succinate
6.2.1.5
5.4.99.2
3.1.2.17
(R)-Methyl-malonyl-CoA
Methyl-malonate
1.1.2.8
Pantothenate and CoA biosynthesis
Citrate cycle
1.2.1.18
2-Propyn-1-ol
00640 11/13/13
(c) Kanehisa Laboratories

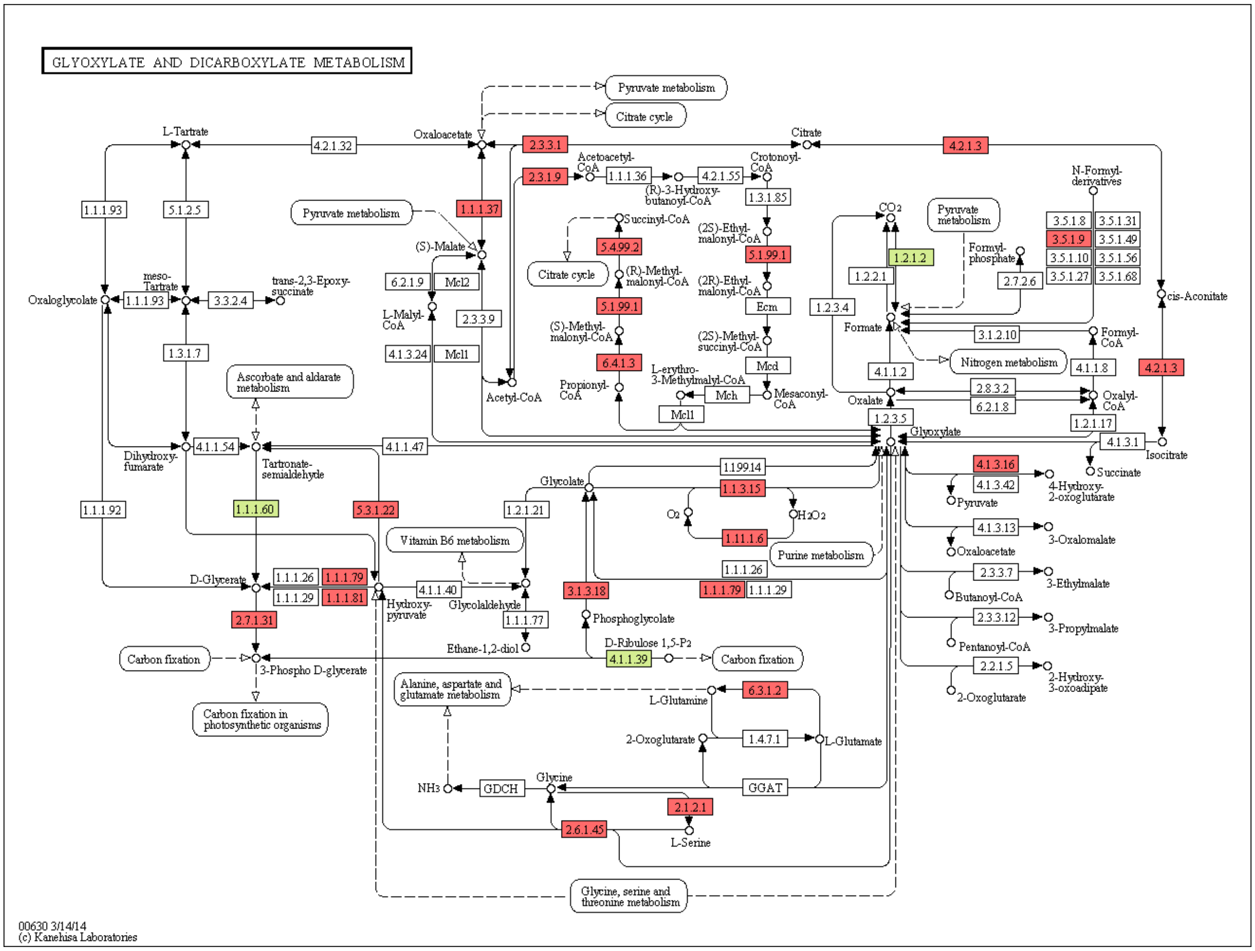
GLYOXYLATE AND DICARBOXYLATE METABOLISM
Pyruvate metabolism
Citrate cycle
L-Tartrate
4.2.1.32
Oxaloacetate
2.3.3.1
Citrate
4.2.1.3
Acetoacetyl-CoA
2.3.1.9
1.1.1.36
(R)-3-Hydroxy-butanoyl-CoA
4.2.1.55
Crotonoyl-CoA
1.3.1.85
N-Formyl-derivatives
1.1.1.93
5.1.2.5
Pyruvate metabolism
1.1.1.37
Succinyl-CoA
(2S)-Ethyl-malonyl-CoA
CO2
Pyruvate metabolism
3.5.1.8
3.5.1.31
3.5.1.9
3.5.1.49
3.5.1.10
3.5.1.56
3.5.1.27
3.5.1.68
(S)-Malate
5.4.99.2
5.1.99.1
1.2.1.2
Formyl-phosphate
Citrate cycle
(R)-Methyl-malonyl-CoA
(2R)-Ethyl-malonyl-CoA
1.2.2.1
2.7.2.6
meso-Tartrate
trans-2,3-Epoxy-succinate
6.2.1.9
Mcl2
Oxaloglycolate
1.1.1.93
3.3.2.4
5.1.99.1
Ecm
1.2.3.4
cis-Aconitate
L-Malyl-CoA
2.3.3.9
(S)-Methyl-malonyl-CoA
(2S)-Methyl-succinyl-CoA
Formate
3.1.2.10
Formyl-CoA
1.3.1.7
4.1.3.24
Mcl1
Nitrogen metabolism
Ascorbate and aldarate metabolism
6.4.1.3
L-erythro-3-Methylmalyl-CoA
Mcd
4.1.1.2
4.1.1.8
4.2.1.3
Acetyl-CoA
Propionyl-CoA
Mch
Mesaconyl-CoA
2.8.3.2
Oxalate
Oxalyl-CoA
6.2.1.8
Mcl1
1.2.3.5
1.2.1.17
Dihydroxy-fumarate
4.1.1.54
4.1.1.47
Glyoxylate
4.1.3.1
Isocitrate
Tartronate-semialdehyde
1.1.99.14
4.1.3.16
Succinate
Glycolate
1.1.3.15
4.1.3.42
4-Hydroxy-2-oxoglutarate
1.1.1.92
1.1.1.60
5.3.1.22
1.2.1.21
O2
H2O2
Pyruvate
Vitamin B6 metabolism
1.11.1.6
4.1.3.13
3-Oxalomalate
Purine metabolism
Oxaloacetate
1.1.1.26
2.3.3.7
3-Ethylmalate
D-Glycerate
1.1.1.26
1.1.1.79
4.1.1.40
3.1.3.18
1.1.1.79
1.1.1.29
Butanoyl-CoA
1.1.1.29
1.1.1.81
Hydroxy-pyruvate
Glycolaldehyde
2.3.3.12
3-Propylmalate
2.7.1.31
1.1.1.77
Phosphoglycolate
Ethane-1,2-diol
D-Ribulose 1,5-P2
Pentanoyl-CoA
Carbon fixation
3-Phospho D-glycerate
4.1.1.39
Carbon fixation
2.2.1.5
2-Hydroxy-3-oxoadipate
Alanine, aspartate and glutamate metabolism
6.3.1.2
2-Oxoglutarate
L-Glutamine
Carbon fixation in photosynthetic organisms
2-Oxoglutarate
1.4.7.1
L-Glutamate
Glycine
NH3
GDCH
GGAT
2.1.2.1
2.6.1.45
L-Serine
Glycine, serine and threonine metabolism
00630 3/14/14
(c) Kanehisa Laboratories

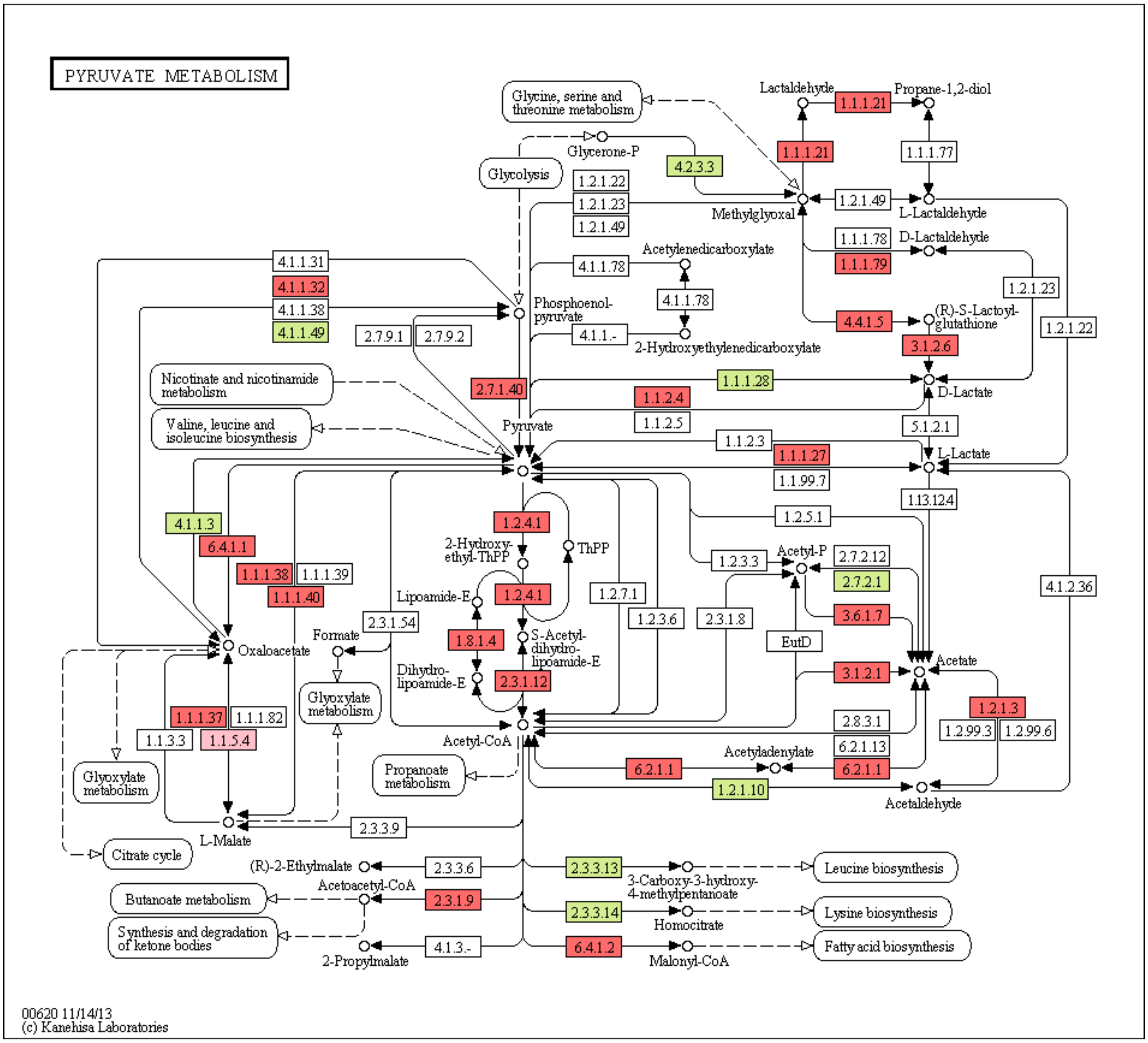
PYRUVATE METABOLISM
Glycine, serine and threonine metabolism
Lactaldehyde
Propane-1,2-diol
1.1.1.21
1.1.1.21
1.1.1.77
Glycerone-P
Glycolysis
4.2.3.3
1.2.1.22
1.2.1.23
1.2.1.49
Methylglyoxal
1.2.1.49
L-Lactaldehyde
1.1.1.78
D-Lactaldehyde
1.1.1.79
Acetylenedicarboxylate
4.1.1.78
4.1.1.78
4.1.1.31
4.1.1.32
4.1.1.38
4.1.1.49
Phosphoenol-pyruvate
2.7.9.1
2.7.9.2
4.1.1.-
2-Hydroxyethylenedicarboxylate
4.4.1.5
(R)-S-Lactoyl-glutathione
1.2.1.23
1.2.1.22
3.1.2.6
Nicotinate and nicotinamide metabolism
2.7.1.40
1.1.2.4
1.1.1.28
D-Lactate
Valine, leucine and isoleucine biosynthesis
1.1.2.5
Pyruvate
5.1.2.1
1.1.2.3
1.1.1.27
L-Lactate
1.1.99.7
1.13.12.4
4.1.1.3
1.2.4.1
1.2.5.1
6.4.1.1
2-Hydroxy-ethyl-ThPP
ThPP
1.1.1.38
1.1.1.39
1.2.3.3
Acetyl-P
2.7.2.12
1.1.1.40
Lipoamide-E
1.2.4.1
1.2.7.1
2.7.2.1
4.1.2.36
2.3.1.54
1.2.3.6
2.3.1.8
3.6.1.7
Formate
1.8.1.4
S-Acetyl-dihydro-lipoamide-E
EutD
Oxaloacetate
Acetate
Dihydro-lipoamide-E
2.3.1.12
3.1.2.1
Glyoxylate metabolism
1.2.1.3
1.1.1.37
1.1.1.82
2.8.3.1
1.2.99.3
1.2.99.6
1.1.3.3
1.1.5.4
Acetyl-CoA
6.2.1.13
Glyoxylate metabolism
Propanoate metabolism
Acetyladenylate
6.2.1.1
6.2.1.1
1.2.1.10
Acetaldehyde
L-Malate
2.3.3.9
Citrate cycle
(R)-2-Ethylmalate
2.3.3.6
2.3.3.13
Leucine biosynthesis
Acetoacetyl-CoA
3-Carboxy-3-hydroxy-4-methylpentanoate
Butanoate metabolism
2.3.1.9
2.3.3.14
Lysine biosynthesis
Homocitrate
Synthesis and degradation of ketone bodies
2-Propylmalate
4.1.3.-
6.4.1.2
Fatty acid biosynthesis
Malonyl-CoA
00620 11/14/13
(c) Kanehisa Laboratories

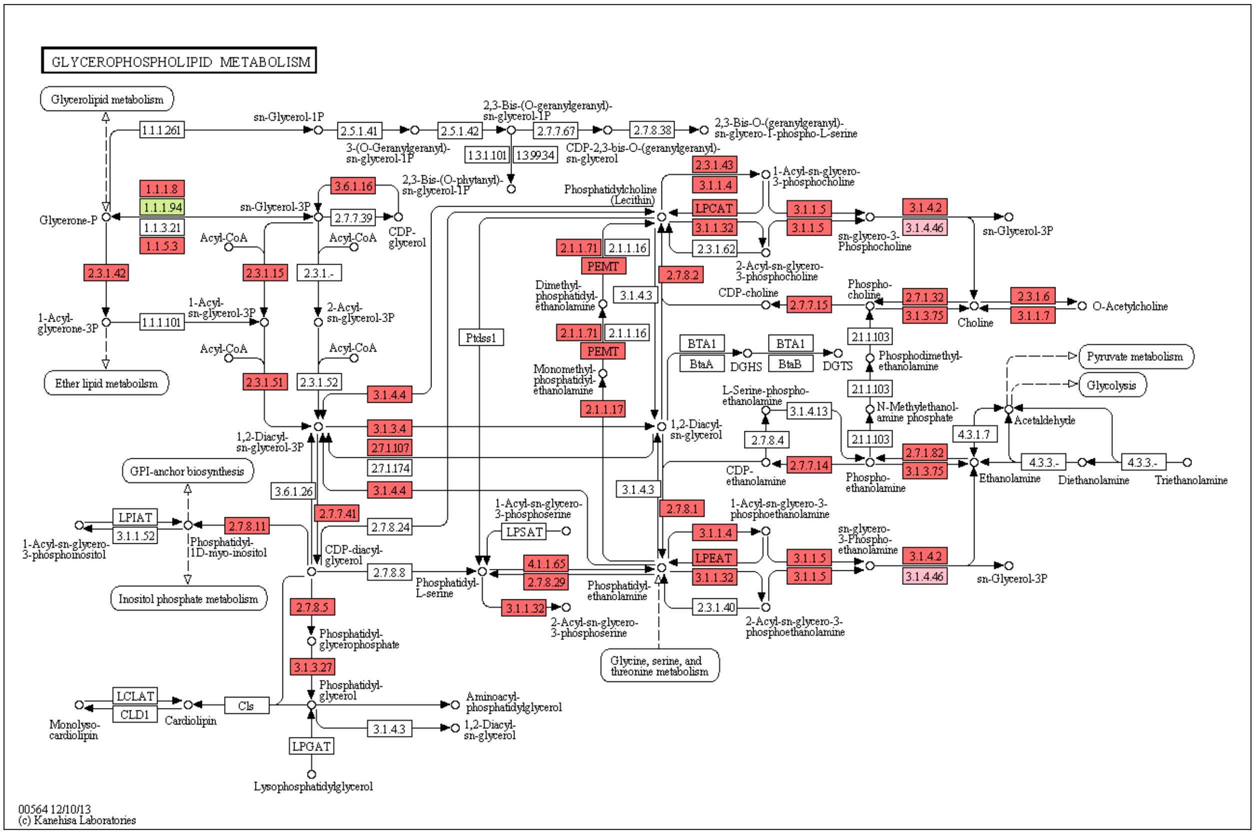

GLYCEROPHOSPHOLIPID METABOLISM
Glycerolipid metabolism
1.1.1.261
sn-Glycerol-1P
2.5.1.41
2.5.1.42
2,3-Bis-(O-geranylgeranyl)-sn-glycerol-1P
2.7.7.67
2.7.8.38
2,3-Bis-O-(geranylgeranyl)-sn-glycero-1-phospho-L-serine
3-(O-Geranylgeranyl)-sn-glycerol-1P
1.3.1.101
1.3.99.34
CDP-2,3-bis-O-(geranylgeranyl)-sn-glycerol
1.1.1.8
1.1.1.94
1.1.3.21
1.1.5.3
Glycerone-P
sn-Glycerol-3P
3.6.1.16
2.7.7.39
2,3-Bis-(O-phytanyl)-sn-glycerol-1P
CDP-glycerol
Acyl-CoA
2.3.1.15
2.3.1.-
2.3.1.42
1-Acyl-glycerone-3P
1.1.1.101
1-Acyl-sn-glycerol-3P
2-Acyl-sn-glycerol-3P
2.3.1.51
2.3.1.52
Ether lipid metaboilsm
3.1.4.4
3.1.3.4
2.7.1.107
2.7.1.174
1,2-Diacyl-sn-glycerol-3P
GPI-anchor biosynthesis
3.6.1.26
LPIAT
3.1.1.52
2.7.8.11
1-Acyl-sn-glycero-3-phosphoinositol
Phosphatidyl-1D-myo-inositol
Inositol phosphate metabolism
2.7.7.41
2.7.8.24
CDP-diacyl-glycerol
2.7.8.8
2.7.8.5
Phosphatidyl-glycerophosphate
3.1.3.27
Phosphatidyl-glycerol
LCLAT
CLD1
Cls
Monolyso-cardiolipin
Cardiolipin
Aminoacyl-phosphatidylglycerol
3.1.4.3
1,2-Diacyl-sn-glycerol
LPGAT
Lysophosphatidylglycerol
Phosphatidylcholine (Lecithin)
2.3.1.43
3.1.1.4
1-Acyl-sn-glycero-3-phosphocholine
LPCAT
3.1.1.32
3.1.1.5
3.1.4.2
3.1.4.46
sn-glycero-3-Phosphocholine
sn-Glycerol-3P
2.3.1.62
2-Acyl-sn-glycero-3-phosphocholine
2.1.1.71
2.1.1.16
PEMT
2.7.8.2
Dimethyl-phosphatidyl-ethanolamine
CDP-choline
2.7.7.15
Phospho-choline
2.7.1.32
3.1.3.75
Choline
2.3.1.6
3.1.1.7
O-Acetylcholine
Ptdss1
Monomethyl-phosphatidyl-ethanolamine
2.1.1.17
BTA1
BtaA
DGHS
BtaB
DGTS
2.1.1.103
Phosphodimethyl-ethanolamine
Pyruvate metabolism
Glycolysis
L-Serine-phospho-ethanolamine
3.1.4.13
N-Methylethanol-amine phosphate
4.3.1.7
Acetaldehyde
2.7.8.4
2.7.7.14
2.7.1.82
CDP-ethanolamine
Phospho-ethanolamine
Ethanolamine
4.3.3.-
Diethanolamine
Triethanolamine
3.1.4.3
2.7.8.1
1-Acyl-sn-glycero-3-phosphoethanolamine
1-Acyl-sn-glycero-3-phosphoserine
LPSAT
sn-glycero-3-Phospho-ethanolamine
4.1.1.65
2.7.8.29
LPEAT
Phosphatidyl-L-serine
Phosphatidyl-ethanolamine
3.1.1.32
2-Acyl-sn-glycero-3-phosphoserine
2.3.1.40
2-Acyl-sn-glycero-3-phosphoethanolamine
Glycine, serine, and threonine metabolism
sn-Glycerol-3P
00564 12/10/13
(c) Kanehisa Laboratories

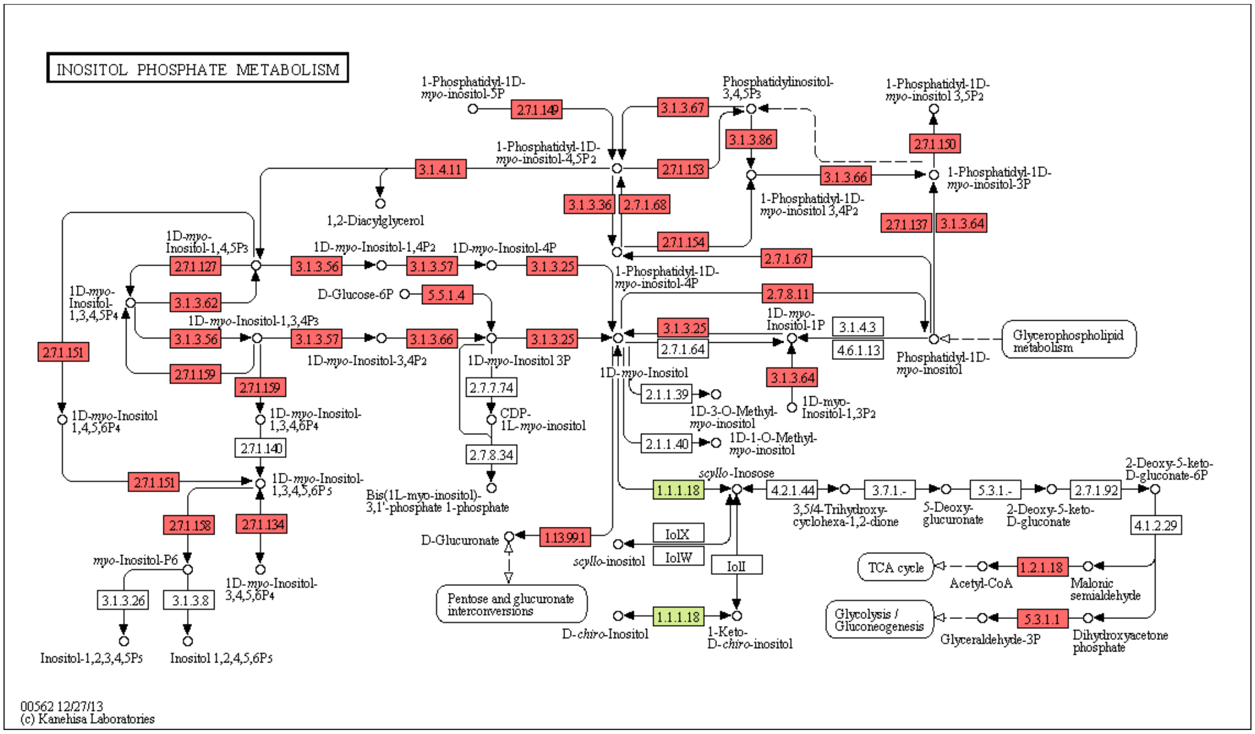
INOSITOL PHOSPHATE METABOLISM
1-Phosphatidyl-1D-myo-inositol-5P
2.7.1.149
3.1.3.67
Phosphatidylinositol-3,4,5P3
1-Phosphatidyl-1D-myo-inositol 3,5P2
3.1.3.86
2.7.1.150
1-Phosphatidyl-1D-myo-inositol-4,5P2
3.1.4.11
2.7.1.153
3.1.3.66
1-Phosphatidyl-1D-myo-inositol-3P
1,2-Diacylglycerol
3.1.3.36
2.7.1.68
1-Phosphatidyl-1D-myo-inositol 3,4P2
2.7.1.137
3.1.3.64
2.7.1.154
1D-myo-Inositol-1,4,5P3
1D-myo-Inositol-1,4P2
1D-myo-Inositol-4P
2.7.1.127
3.1.3.56
3.1.3.57
3.1.3.25
2.7.1.67
1-Phosphatidyl-1D-myo-inositol-4P
1D-myo-Inositol-1,3,4,5P4
3.1.3.62
D-Glucose-6P
5.5.1.4
2.7.8.11
1D-myo-Inositol-1,3,4P3
1D-myo-Inositol-1P
3.1.4.3
3.1.3.56
3.1.3.57
3.1.3.66
3.1.3.25
3.1.3.25
2.7.1.64
4.6.1.13
Glycerophospholipid metabolism
2.7.1.151
1D-myo-Inositol-3,4P2
1D-myo-Inositol 3P
1D-myo-Inositol
Phosphatidyl-1D-myo-inositol
2.7.1.159
2.7.1.159
2.7.7.74
2.1.1.39
3.1.3.64
1D-myo-Inositol 1,4,5,6P4
1D-myo-Inositol-1,3,4,6P4
CDP-1L-myo-inositol
1D-3-O-Methyl-myo-inositol
1D-myo-Inositol-1,3P2
2.7.1.140
2.7.8.34
2.1.1.40
1D-1-O-Methyl-myo-inositol
scyllo-Inosose
2-Deoxy-5-keto-D-gluconate-6P
2.7.1.151
1D-myo-Inositol-1,3,4,5,6P5
1.1.1.18
4.2.1.44
3.7.1.-
5.3.1.-
2.7.1.92
Bis(1L-myo-inositol)-3,1'-phosphate 1-phosphate
3,5/4-Trihydroxy-cyclohexa-1,2-dione
5-Deoxy-glucuronate
2-Deoxy-5-keto-D-gluconate
2.7.1.158
2.7.1.134
D-Glucuronate
1.13.99.1
IolX
4.1.2.29
scyllo-inositol
IolW
IolI
myo-Inositol-P6
TCA cycle
Acetyl-CoA
1.2.1.18
Malonic semialdehyde
1D-myo-Inositol-3,4,5,6P4
3.1.3.26
3.1.3.8
Pentose and glucuronate interconversions
1.1.1.18
Glycolysis / Gluconeogenesis
5.3.1.1
D-chiro-Inositol
1-Keto-D-chiro-inositol
Glyceraldehyde-3P
Dihydroxyacetone phosphate
Inositol-1,2,3,4,5P5
Inositol 1,2,4,5,6P5
00562 12/27/13
(c) Kanehisa Laboratories

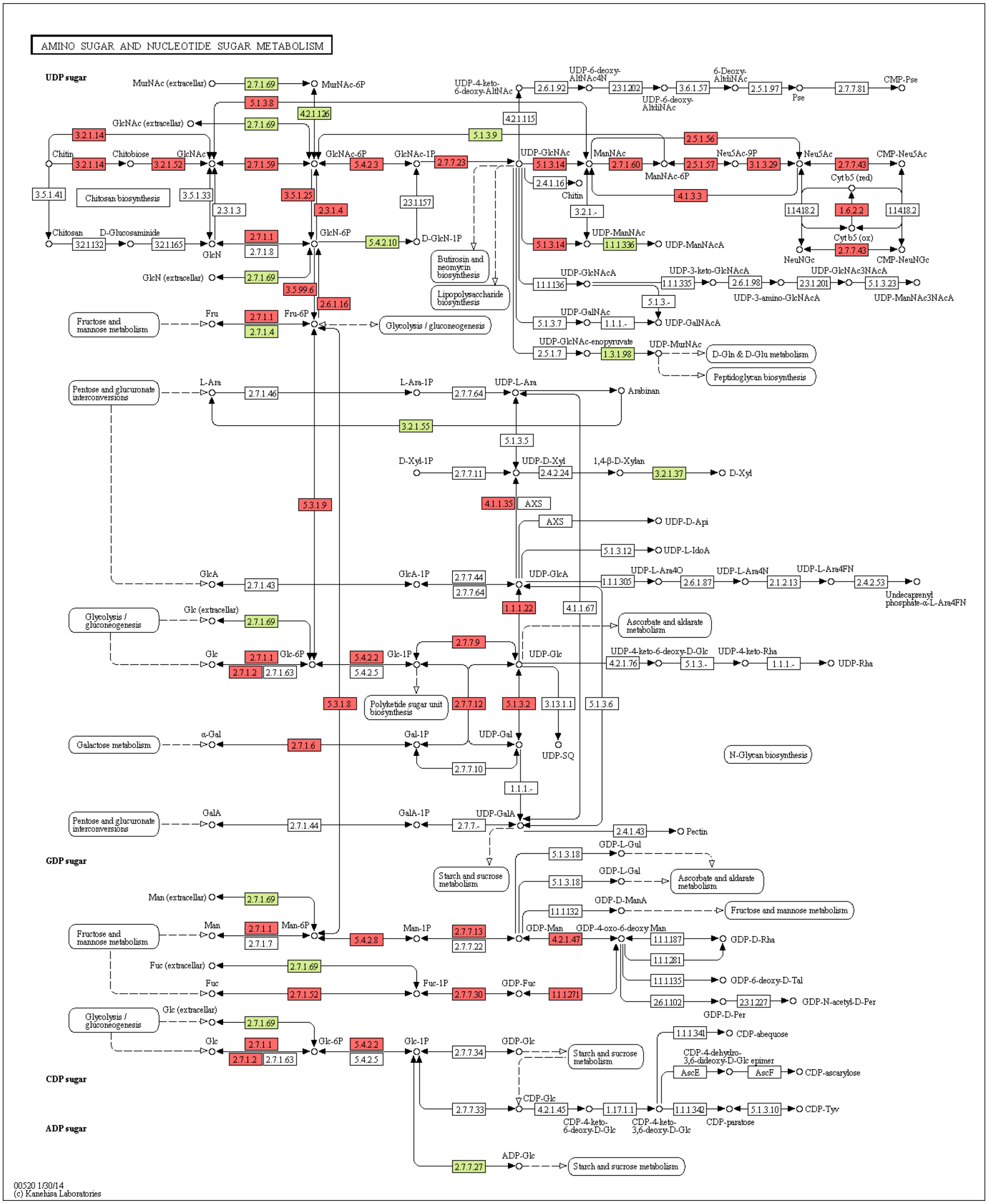

AMINO SUGAR AND NUCLEOTIDE SUGAR METABOLISM
UDP sugar
MurNAc (extracellar)
2.7.1.69
MurNAc-6P
5.1.3.8
4.2.1.126
GlcNAc (extracellar)
2.7.1.69
UDP-4-keto-6-deoxy-AltNAc
2.6.1.92
UDP-6-deoxy-AltNAc4N
2.3.1.202
UDP-6-deoxy-AltdiNAc
3.6.1.57
6-Deoxy-AltdiNAc
2.5.1.97
Pse
2.7.7.81
CMP-Pse
4.2.1.115
5.1.3.9
2.5.1.56
3.2.1.14
Chitin
3.2.1.14
Chitobiose
3.2.1.52
GlcNAc
2.7.1.59
GlcNAc-6P
5.4.2.3
GlcNAc-1P
2.7.7.23
UDP-GlcNAc
5.1.3.14
ManNAc
2.7.1.60
ManNAc-6P
2.5.1.57
Neu5Ac-9P
3.1.3.29
Neu5Ac
2.7.7.43
CMP-Neu5Ac
2.4.1.16
Chitin
4.1.3.3
Cyt b5 (red)
3.5.1.41
Chitosan biosynthesis
3.5.1.33
2.3.1.3
3.5.1.25
2.3.1.4
2.3.1.157
3.2.1.-
1.14.18.2
1.6.2.2
1.14.18.2
Chitosan
3.2.1.132
D-Glucosaminide
3.2.1.165
GlcN
2.7.1.1
2.7.1.8
GlcN-6P
5.4.2.10
D-GlcN-1P
5.1.3.14
UDP-ManNAc
1.1.1.336
UDP-ManNAcA
Cyt b5 (ox)
NeuNGc
2.7.7.43
CMP-NeuNGc
GlcN (extracellar)
2.7.1.69
Butirosin and neomycin biosynthesis
Lipopolysaccharide biosynthesis
3.5.99.6
2.6.1.16
UDP-GlcNAcA
1.1.1.136
UDP-3-keto-GlcNAcA
1.1.1.335
2.6.1.98
UDP-3-amino-GlcNAcA
UDP-GlcNAc3NAcA
2.3.1.201
5.1.3.23
UDP-ManNAc3NAcA
5.1.3.-
UDP-GalNAc
5.1.3.7
1.1.1.-
UDP-GalNAcA
Fructose and mannose metabolism
Fru
2.7.1.1
2.7.1.4
Fru-6P
Glycolysis / gluconeogenesis
UDP-GlcNAc-enopyruvate
2.5.1.7
1.3.1.98
UDP-MurNAc
D-Gln & D-Glu metabolism
Peptidoglycan biosynthesis
Pentose and glucuronate interconversions
L-Ara
2.7.1.46
L-Ara-1P
2.7.7.64
UDP-L-Ara
Arabinan
3.2.1.55
5.1.3.5
D-Xyl-1P
2.7.7.11
UDP-D-Xyl
2.4.2.24
1,4-β-D-Xylan
3.2.1.37
D-Xyl
5.3.1.9
4.1.1.35
AXS
AXS
UDP-D-Api
5.1.3.12
UDP-L-IdoA
GlcA
2.7.1.43
GlcA-1P
2.7.7.44
2.7.7.64
UDP-GlcA
1.1.1.305
UDP-L-Ara4O
2.6.1.87
UDP-L-Ara4N
2.1.2.13
UDP-L-Ara4FN
2.4.2.53
Undecaprenyl phosphate-α-L-Ara4FN
1.1.1.22
4.1.1.67
Glycolysis / gluconeogenesis
Glc (extracellar)
2.7.1.69
Ascorbate and aldarate metabolism
2.7.7.9
Glc
2.7.1.1
2.7.1.2
2.7.1.63
Glc-6P
5.4.2.2
5.4.2.5
Glc-1P
UDP-Glc
UDP-4-keto-6-deoxy-D-Glc
4.2.1.76
5.1.3.-
UDP-4-keto-Rha
1.1.1.-
UDP-Rha
5.3.1.8
Polyketide sugar unit biosynthesis
2.7.7.12
5.1.3.2
3.13.1.1
5.1.3.6
Galactose metabolism
α-Gal
2.7.1.6
Gal-1P
UDP-Gal
UDP-SQ
N-Glycan biosynthesis
2.7.7.10
1.1.1.-
Pentose and glucuronate interconversions
GalA
2.7.1.44
GalA-1P
2.7.7.-
UDP-GalA
2.4.1.43
Pectin
GDP sugar
GDP-L-Gul
5.1.3.18
Starch and sucrose metabolism
GDP-L-Gal
5.1.3.18
Ascorbate and aldarate metabolism
GDP-D-ManA
1.1.1.132
Fructose and mannose metabolism
Man (extracellar)
2.7.1.69
Fructose and mannose metabolism
Man
2.7.1.1
2.7.1.7
Man-6P
5.4.2.8
Man-1P
2.7.7.13
2.7.7.22
GDP-Man
4.2.1.47
GDP-4-oxo-6-deoxy Man
1.1.1.187
GDP-D-Rha
1.1.1.281
1.1.1.135
GDP-6-deoxy-D-Tal
2.6.1.102
GDP-D-Per
2.3.1.227
GDP-N-acetyl-D-Per
Fuc (extracellar)
2.7.1.69
Fuc
2.7.1.52
Fuc-1P
2.7.7.30
GDP-Fuc
1.1.1.271
Glycolysis / gluconeogenesis
Glc (extracellar)
2.7.1.69
Glc
2.7.1.1
2.7.1.2
2.7.1.63
Glc-6P
5.4.2.2
5.4.2.5
Glc-1P
2.7.7.34
GDP-Glc
Starch and sucrose metabolism
1.1.1.341
CDP-abequose
CDP-4-dehydro-3,6-dideoxy-D-Glc epimer
AscE
AscF
CDP-ascarylose
CDP sugar
2.7.7.33
CDP-Glc
4.2.1.45
CDP-4-keto-6-deoxy-D-Glc
1.17.1.1
CDP-4-keto-3,6-deoxy-D-Glc
1.1.1.342
CDP-paratose
5.1.3.10
CDP-Tyv
ADP sugar
ADP-Glc
2.7.7.27
Starch and sucrose metabolism
00520 1/30/14
(c) Kanehisa Laboratories

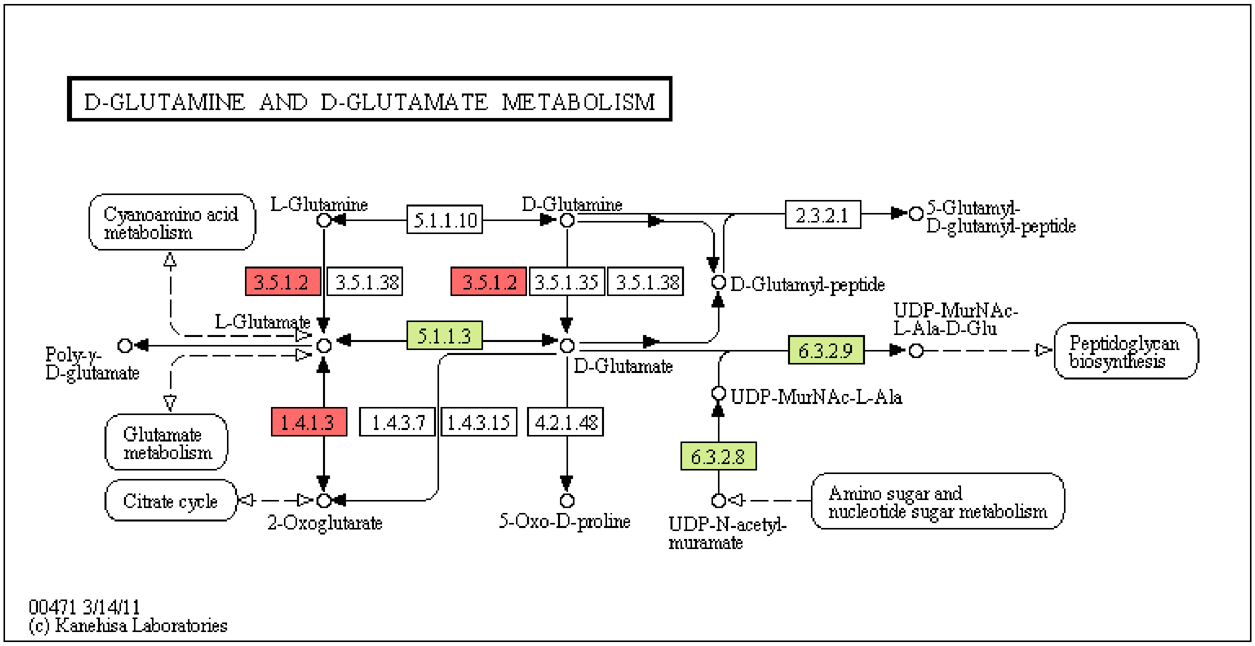
D-GLUTAMINE AND D-GLUTAMATE METABOLISM
Cyanoamino acid metabolism
L-Glutamine
5.1.1.10
D-Glutamine
2.3.2.1
5-Glutamyl-D-glutamyl-peptide
3.5.1.2
3.5.1.38
3.5.1.2
3.5.1.35
3.5.1.38
D-Glutamyl-peptide
L-Glutamate
5.1.1.3
UDP-MurNAc-L-Ala-D-Glu
Poly-γ-D-glutamate
D-Glutamate
6.3.2.9
Peptidoglycan biosynthesis
UDP-MurNAc-L-Ala
Glutamate metabolism
1.4.1.3
1.4.3.7
1.4.3.15
4.2.1.48
6.3.2.8
Citrate cycle
2-Oxoglutarate
5-Oxo-D-proline
UDP-N-acetyl-muramate
Amino sugar and nucleotide sugar metabolism
00471 3/14/11
(c) Kanehisa Laboratories

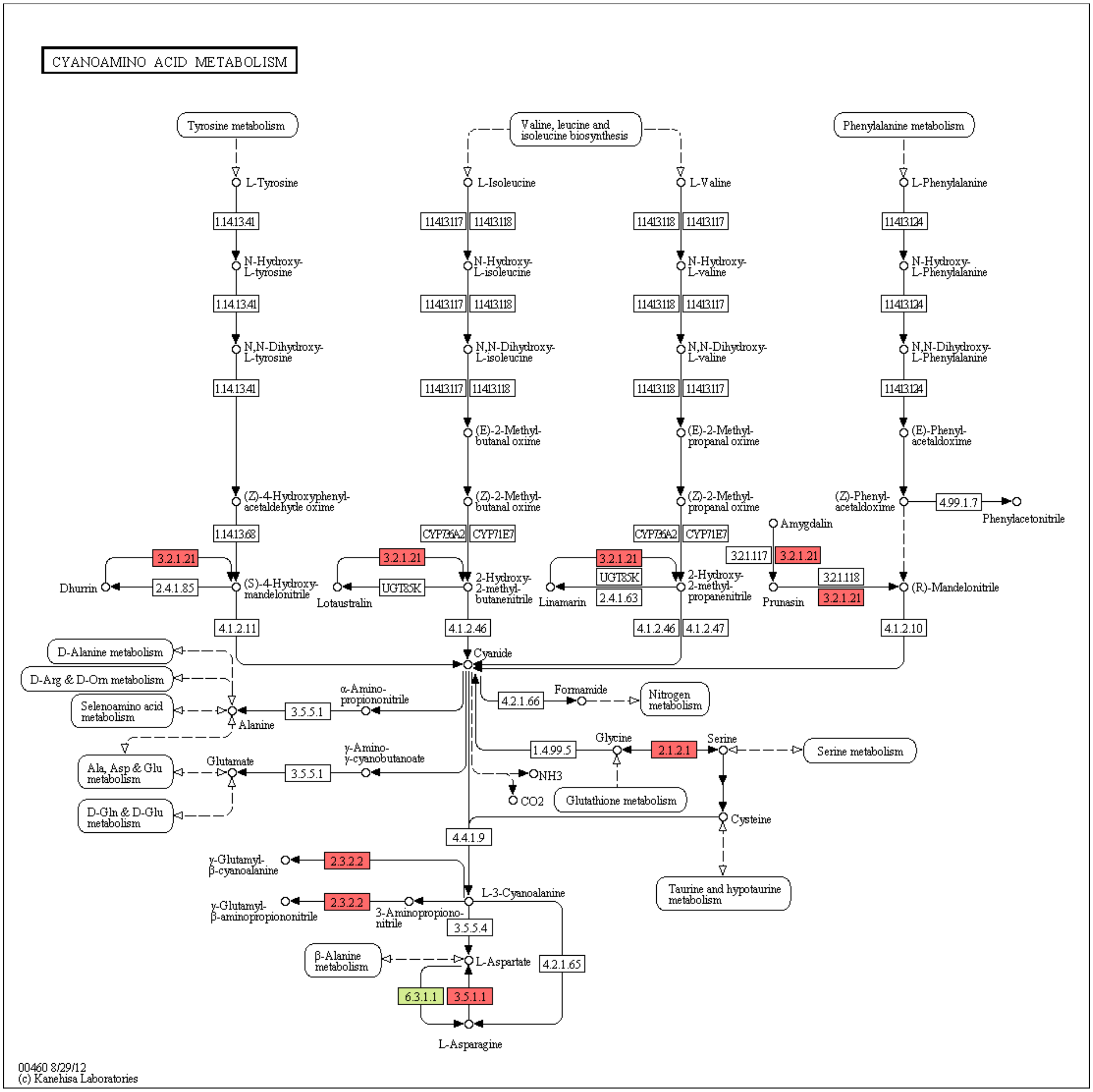

CYANOAMINO ACID METABOLISM
Tyrosine metabolism
Valine, leucine and isoleucine biosynthesis
Phenylalanine metabolism
L-Tyrosine
L-Isoleucine
L-Valine
L-Phenylalanine
1.14.13.41
1.14.13.117
1.14.13.118
1.14.13.124
N-Hydroxy-L-tyrosine
N-Hydroxy-L-isoleucine
N-Hydroxy-L-valine
N-Hydroxy-L-Phenylalanine
N,N-Dihydroxy-L-tyrosine
N,N-Dihydroxy-L-isoleucine
N,N-Dihydroxy-L-valine
N,N-Dihydroxy-L-Phenylalanine
(E)-2-Methyl-butanal oxime
(E)-2-Methyl-propanal oxime
(E)-Phenyl-acetaldoxime
(Z)-4-Hydroxyphenyl-acetaldehyde oxime
(Z)-2-Methyl-butanal oxime
(Z)-2-Methyl-propanal oxime
(Z)-Phenyl-acetaldoxime
4.99.1.7
Phenylacetonitrile
1.14.13.68
CYP79A2
CYP71E7
Amygdalin
3.2.1.117
3.2.1.21
3.2.1.118
Dhurrin
2.4.1.85
(S)-4-Hydroxy-mandelonitrile
UGT85K
Lotaustralin
2-Hydroxy-2-methyl-butanenitrile
Linamarin
2.4.1.63
2-Hydroxy-2-methyl-propanenitrile
Prunasin
(R)-Mandelonitrile
4.1.2.11
4.1.2.46
4.1.2.47
4.1.2.10
Cyanide
D-Alanine metabolism
D-Arg & D-Orn metabolism
Selenoamino acid metabolism
3.5.5.1
α-Amino-propiononitrile
Alanine
4.2.1.66
Formamide
Nitrogen metabolism
Ala, Asp & Glu metabolism
Glutamate
γ-Amino-γ-cyanobutanoate
1.4.99.5
Glycine
2.1.2.1
Serine
Serine metabolism
NH3
CO2
Glutathione metabolism
D-Gln & D-Glu metabolism
Cysteine
4.4.1.9
γ-Glutamyl-β-cyanoalanine
2.3.2.2
L-3-Cyanoalanine
γ-Glutamyl-β-aminopropiononitrile
3-Aminopropiono-nitrile
3.5.5.4
Taurine and hypotaurine metabolism
β-Alanine metabolism
L-Aspartate
4.2.1.65
6.3.1.1
3.5.1.1
L-Asparagine
00460 8/29/12
(c) Kanehisa Laboratories

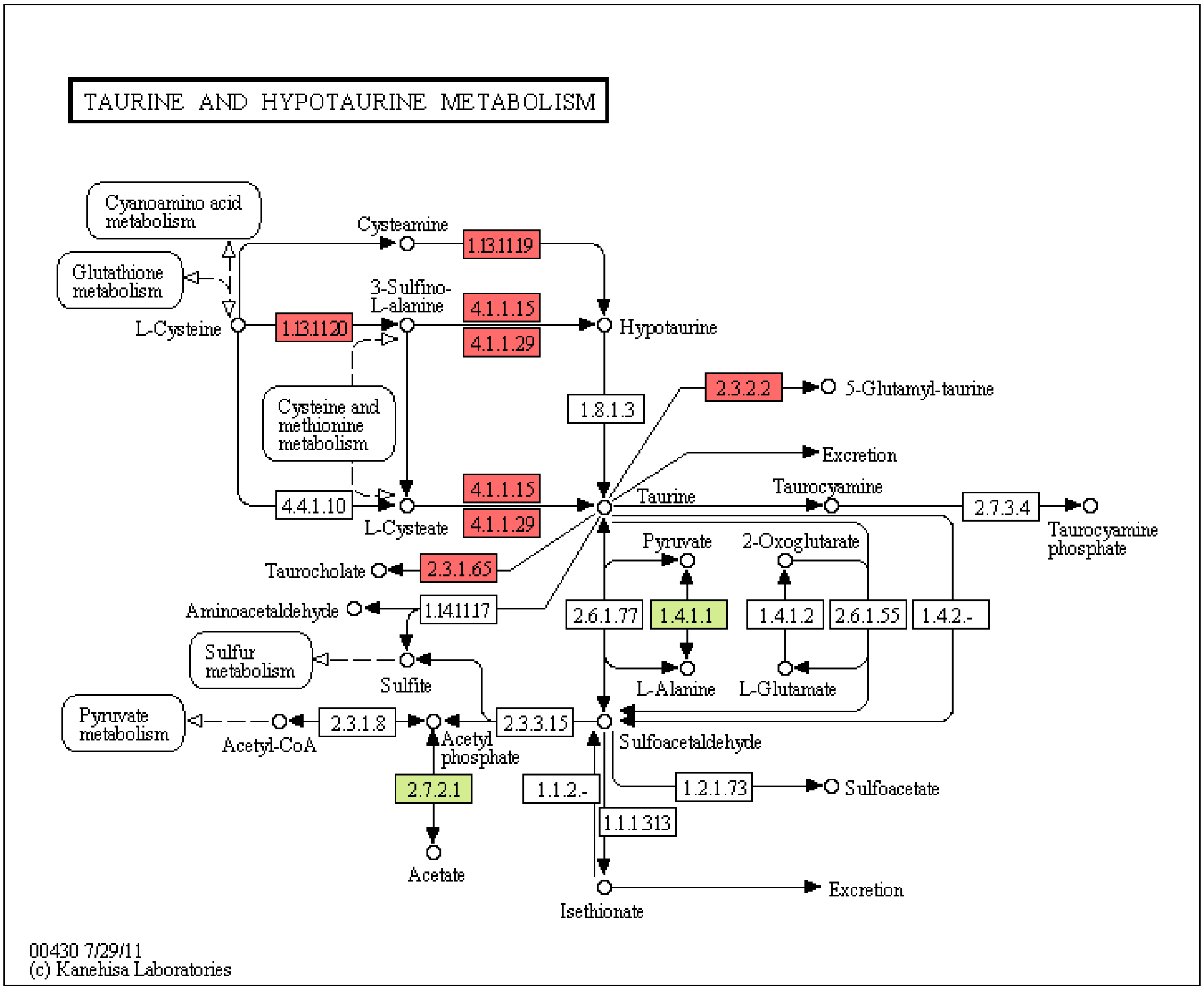

TAURINE AND HYPOTAURINE METABOLISM
Cyanoamino acid metabolism
Glutathione metabolism
Cysteamine
1.13.11.19
3-Sulfino-L-alanine
4.1.1.15
4.1.1.29
L-Cysteine
1.13.11.20
Hypotaurine
Cysteine and methionine metabolism
1.8.1.3
2.3.2.2
5-Glutamyl-taurine
Excretion
4.4.1.10
4.1.1.15
4.1.1.29
L-Cysteate
Taurine
Taurocyamine
2.7.3.4
Taurocyamine phosphate
Taurocholate
2.3.1.65
Pyruvate
2-Oxoglutarate
Aminoacetaldehyde
1.14.11.17
2.6.1.77
1.4.1.1
1.4.1.2
2.6.1.55
1.4.2.-
Sulfur metabolism
Sulfite
L-Alanine
L-Glutamate
Pyruvate metabolism
2.3.1.8
2.3.3.15
Acetyl-CoA
Acetyl phosphate
Sulfoacetaldehyde
2.7.2.1
1.1.2.-
1.2.1.73
Sulfoacetate
1.1.1.313
Acetate
Isethionate
Excretion
00430 7/29/11
(c) Kanehisa Laboratories

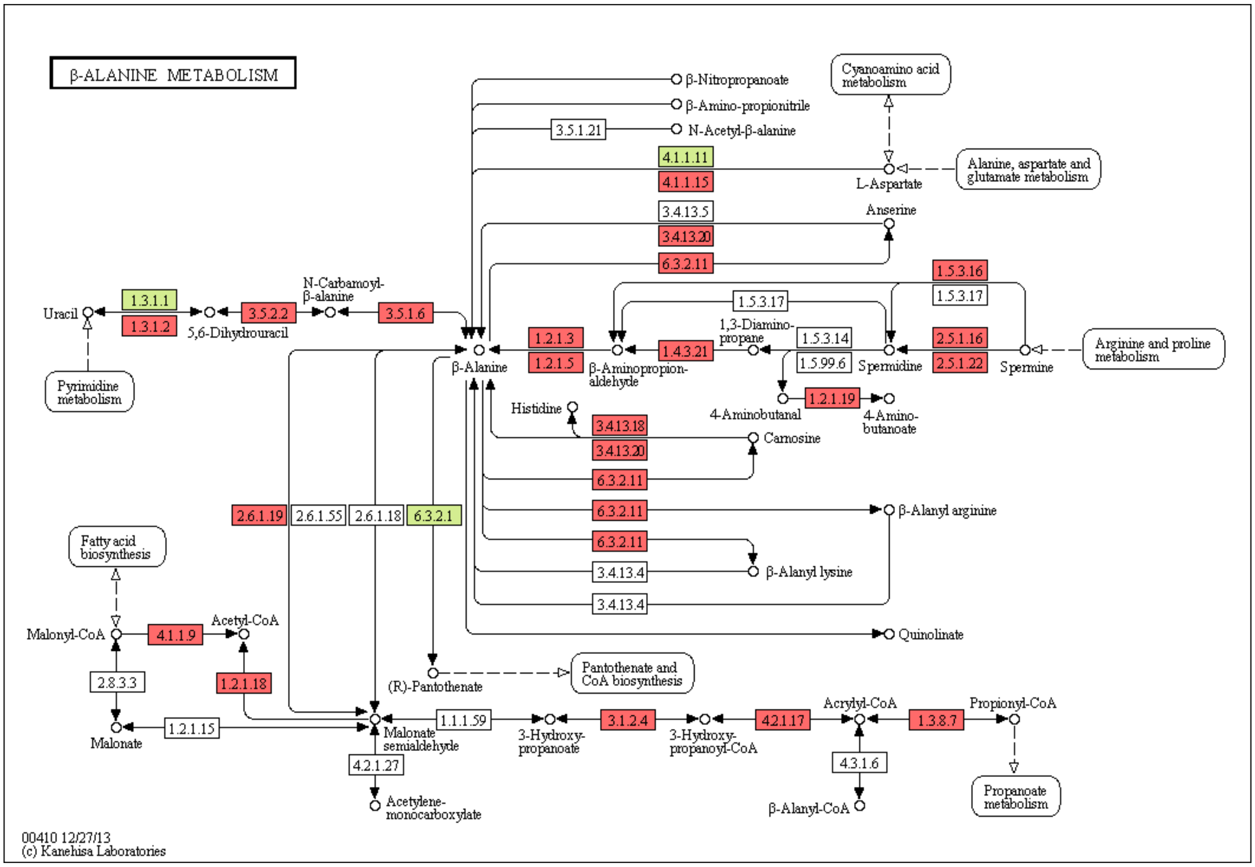
β-ALANINE METABOLISM
β-Nitropropanoate
β-Amino-propionitrile
N-Acetyl-β-alanine
3.5.1.21
4.1.1.11
4.1.1.15
L-Aspartate
Cyanoamino acid metabolism
Alanine, aspartate and glutamate metabolism
3.4.13.5
3.4.13.20
6.3.2.11
Anserine
1.5.3.16
1.5.3.17
1.5.3.17
1,3-Diamino-propane
1.5.3.14
1.5.99.6
2.5.1.16
2.5.1.22
Spermidine
Spermine
Arginine and proline metabolism
Uracil
1.3.1.1
1.3.1.2
5,6-Dihydrouracil
3.5.2.2
N-Carbamoyl-β-alanine
3.5.1.6
β-Alanine
1.2.1.3
1.2.1.5
β-Aminopropion-aldehyde
1.4.3.21
4-Aminobutanal
1.2.1.19
4-Amino-butanoate
Pyrimidine metabolism
Histidine
3.4.13.18
3.4.13.20
Carnosine
6.3.2.11
2.6.1.19
2.6.1.55
2.6.1.18
6.3.2.1
6.3.2.11
β-Alanyl arginine
6.3.2.11
β-Alanyl lysine
3.4.13.4
3.4.13.4
Quinolinate
Fatty acid biosynthesis
Malonyl-CoA
4.1.1.9
Acetyl-CoA
2.8.3.3
1.2.1.18
(R)-Pantothenate
Pantothenate and CoA biosynthesis
Malonate
1.2.1.15
Malonate semialdehyde
1.1.1.59
3-Hydroxy-propanoate
3.1.2.4
3-Hydroxy-propanoyl-CoA
4.2.1.17
Acrylyl-CoA
1.3.8.7
Propionyl-CoA
4.2.1.27
Acetylene-monocarboxylate
4.3.1.6
β-Alanyl-CoA
Propanoate metabolism
00410 12/27/13
(c) Kanehisa Laboratories

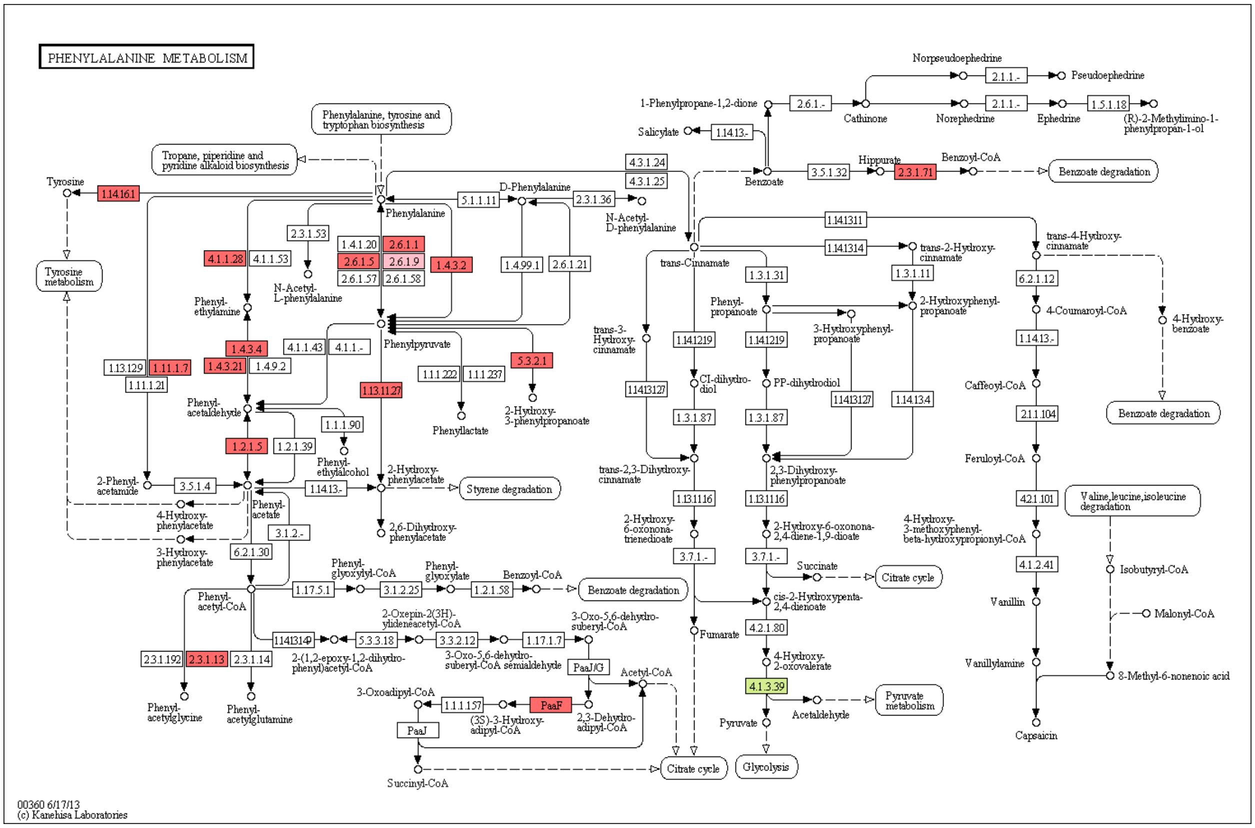

PHENYLALANINE METABOLISM
Phenylalanine, tyrosine and tryptophan biosynthesis
Tropane, piperidine and pyridine alkaloid biosynthesis
Tyrosine
1.14.16.1
Tyrosine metabolism
Phenylalanine
D-Phenylalanine
5.1.1.11
2.3.1.36
N-Acetyl-D-phenylalanine
4.3.1.24
4.3.1.25
2.3.1.53
N-Acetyl-L-phenylalanine
1.4.1.20
2.6.1.1
2.6.1.5
2.6.1.9
2.6.1.57
2.6.1.58
1.4.3.2
1.4.99.1
2.6.1.21
4.1.1.28
4.1.1.53
Phenyl-ethylamine
1.4.3.4
1.4.3.21
1.4.9.2
4.1.1.43
4.1.1.-
Phenylpyruvate
1.13.12.9
1.11.1.7
1.11.1.21
Phenyl-acetaldehyde
1.13.11.27
1.1.1.222
1.1.1.237
5.3.2.1
Phenyllactate
2-Hydroxy-3-phenylpropanoate
1.1.1.90
Phenyl-ethylalcohol
1.2.1.5
1.2.1.39
2-Phenyl-acetamide
3.5.1.4
1.14.13.-
2-Hydroxy-phenylacetate
Styrene degradation
2,6-Dihydroxy-phenylacetate
4-Hydroxy-phenylacetate
Phenyl-acetate
3.1.2.-
3-Hydroxy-phenylacetate
6.2.1.30
Phenyl-acetyl-CoA
Phenyl-glyoxylyl-CoA
1.17.5.1
3.1.2.25
Phenyl-glyoxylate
1.2.1.58
Benzoyl-CoA
Benzoate degradation
2-Oxepin-2(3H)-ylideneacetyl-CoA
3-Oxo-5,6-dehydro-suberyl-CoA
1.14.13.149
5.3.3.18
3.3.2.12
1.17.1.7
2-(1,2-epoxy-1,2-dihydro-phenyl)acetyl-CoA
3-Oxo-5,6-dehydro-suberyl-CoA semialdehyde
PaaJ/G
Acetyl-CoA
2.3.1.192
2.3.1.13
2.3.1.14
Phenyl-acetylglycine
Phenyl-acetylglutamine
3-Oxoadipyl-CoA
1.1.1.157
PaaF
(3S)-3-Hydroxy-adipyl-CoA
2,3-Dehydro-adipyl-CoA
PaaJ
Succinyl-CoA
Citrate cycle
1-Phenylpropane-1,2-dione
2.6.1.-
Cathinone
Norpseudoephedrine
2.1.1.-
Pseudoephedrine
Norephedrine
Ephedrine
1.5.1.18
(R)-2-Methylimino-1-phenylpropan-1-ol
Salicylate
1.14.13.-
Benzoate
3.5.1.32
Hippurate
2.3.1.71
Benzoyl-CoA
Benzoate degradation
1.14.13.11
trans-4-Hydroxy-cinnamate
1.14.13.14
trans-2-Hydroxy-cinnamate
trans-Cinnamate
1.3.1.31
1.3.1.11
Phenyl-propanoate
2-Hydroxyphenyl-propanoate
3-Hydroxyphenyl-propanoate
6.2.1.12
4-Coumaroyl-CoA
4-Hydroxy-benzoate
trans-3-Hydroxy-cinnamate
1.14.12.19
1.14.12.19
1.14.13.127
CI-dihydro-diol
PP-dihydrodiol
1.14.13.127
1.14.13.4
1.14.13.-
Caffeoyl-CoA
1.3.1.87
1.3.1.87
2.1.1.104
Benzoate degradation
trans-2,3-Dihydroxy-cinnamate
2,3-Dihydroxy-phenylpropanoate
Feruloyl-CoA
1.13.11.16
1.13.11.16
4.2.1.101
Valine, leucine, isoleucine degradation
2-Hydroxy-6-oxonona-trienedioate
2-Hydroxy-6-oxonona-2,4-diene-1,9-dioate
4-Hydroxy-3-methoxyphenyl-beta-hydroxypropionoyl-CoA
3.7.1.-
3.7.1.-
Succinate
Citrate cycle
4.1.2.41
Isobutyryl-CoA
cis-2-Hydroxypenta-2,4-dienoate
Vanillin
Malonyl-CoA
Fumarate
4.2.1.80
4-Hydroxy-2-oxovalerate
Vanillylamine
8-Methyl-6-nonenoic acid
4.1.3.39
Acetaldehyde
Pyruvate metabolism
Pyruvate
Capsaicin
Citrate cycle
Glycolysis
00360 6/17/13
(c) Kanehisa Laboratories

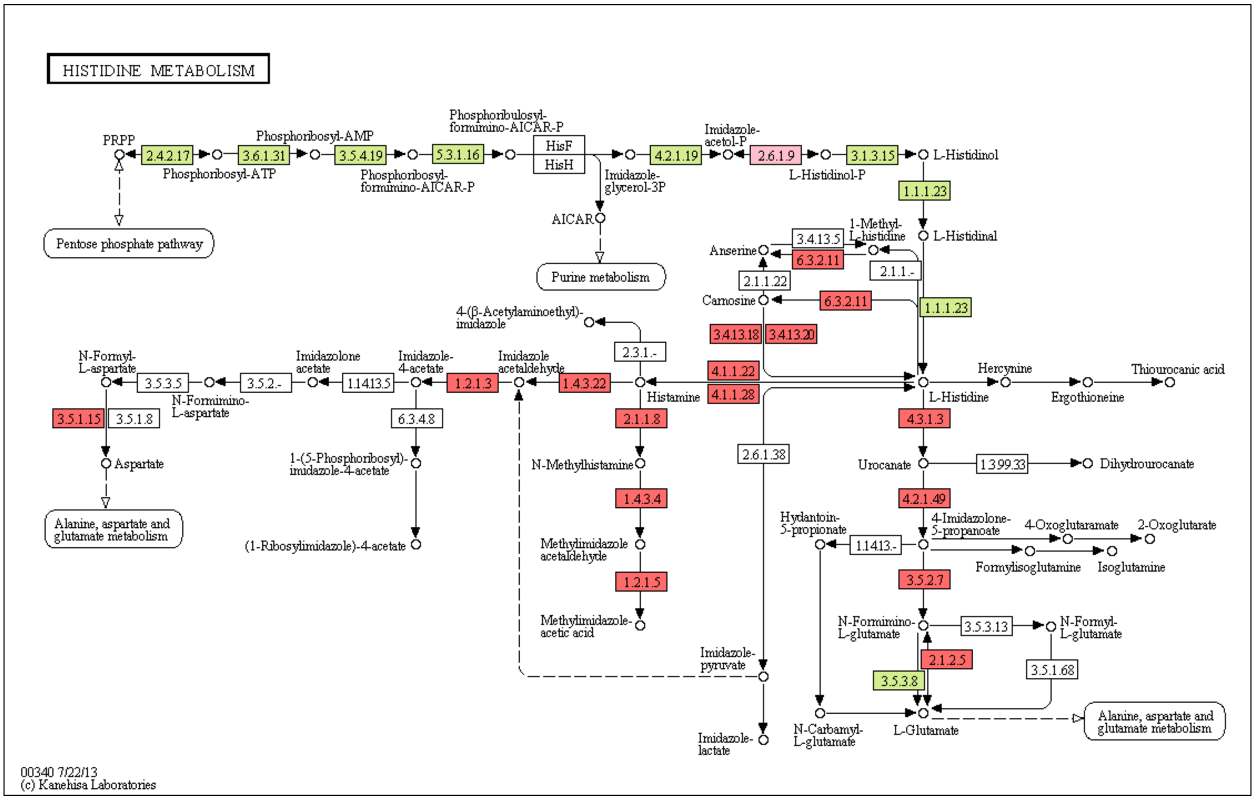
HISTIDINE METABOLISM
PRPP
2.4.2.17
Phosphoribosyl-ATP
3.6.1.31
Phosphoribosyl-AMP
3.5.4.19
Phosphoribosyl-formimino-AICAR-P
5.3.1.16
Phosphoribulosyl-formimino-AICAR-P
HisF
HisH
Imidazole-glycerol-3P
4.2.1.19
Imidazole-acetol-P
2.6.1.9
L-Histidinol-P
3.1.3.15
L-Histidinol
1.1.1.23
L-Histidinal
Pentose phosphate pathway
AICAR
Purine metabolism
1-Methyl-L-histidine
3.4.13.5
Anserine
6.3.2.11
2.1.1.-
2.1.1.22
Carnosine
6.3.2.11
1.1.1.23
4-(β-Acetylaminoethyl)-imidazole
3.4.13.18
3.4.13.20
2.3.1.-
N-Formyl-L-aspartate
3.5.3.5
N-Formimino-L-aspartate
3.5.2.-
Imidazolone acetate
1.14.13.5
Imidazole-4-acetate
1.2.1.3
Imidazole acetaldehyde
1.4.3.22
Histamine
4.1.1.22
4.1.1.28
L-Histidine
Hercynine
Ergothioneine
Thiourocanic acid
3.5.1.15
3.5.1.8
6.3.4.8
2.1.1.8
2.6.1.38
4.3.1.3
Aspartate
1-(5-Phosphoribosyl)-imidazole-4-acetate
N-Methylhistamine
Urocanate
1.3.99.33
Dihydrourocanate
Alanine, aspartate and glutamate metabolism
1.4.3.4
4.2.1.49
Hydantoin-5-propionate
1.14.13.-
4-Imidazolone-5-propanoate
4-Oxoglutaramate
2-Oxoglutarate
Formylisoglutamine
Isoglutamine
(1-Ribosylimidazole)-4-acetate
Methylimidazole acetaldehyde
1.2.1.5
3.5.2.7
Methylimidazole-acetic acid
N-Formimino-L-glutamate
3.5.3.13
N-Formyl-L-glutamate
Imidazole-pyruvate
2.1.2.5
3.5.3.8
3.5.1.68
N-Carbamyl-L-glutamate
L-Glutamate
Alanine, aspartate and glutamate metabolism
Imidazole-lactate
00340 7/22/13
(c) Kanehisa Laboratories

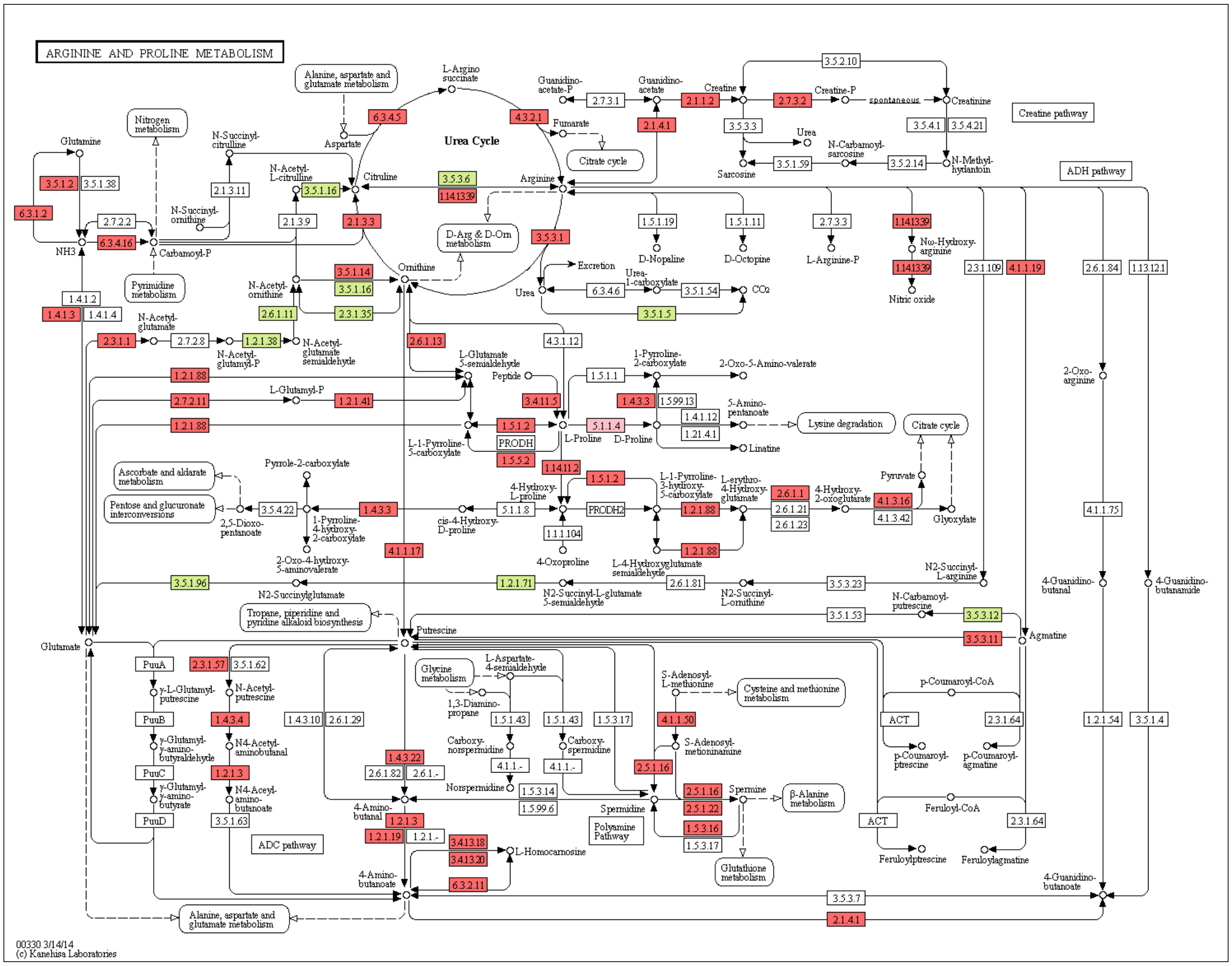
ARGININE AND PROLINE METABOLISM
Urea Cycle
Alanine, aspartate and glutamate metabolism
Nitrogen metabolism
Pyrimidine metabolism
Citrate cycle
D-Arg & D-Orn metabolism
Creatine pathway
ADH pathway
Lysine degradation
Ascorbate and aldarate metabolism
Pentose and glucuronate interconversions
Tropane, piperidine and pyridine alkaloid biosynthesis
Glycine metabolism
Cysteine and methionine metabolism
β-Alanine metabolism
Polyamine Pathway
Glutathione metabolism
ADC pathway
Glutamine
NH3
Carbamoyl-P
N-Succinyl-citrulline
N-Succinyl-ornithine
N-Acetyl-L-citrulline
Citrulline
Aspartate
L-Argino succinate
Fumarate
Arginine
Ornithine
Urea
Excretion
Urea-1-carboxylate
CO2
N-Acetyl-ornithine
N-Acetyl-glutamate
N-Acetyl-glutamyl-P
N-Acetyl-glutamate semialdehyde
Guanidino-acetate-P
Guanidino-acetate
Creatine
Creatine-P
spontaneous
Creatinine
Sarcosine
N-Carbamoyl-sarcosine
N-Methyl-hydantoin
D-Nopaline
D-Octopine
L-Arginine-P
Nω-Hydroxy-arginine
Nitric oxide
L-Glutamate 5-semialdehyde
L-Glutamyl-P
Peptide
L-Proline
D-Proline
1-Pyrroline-2-carboxylate
2-Oxo-5-Amino-valerate
5-Amino-pentanoate
Linatine
L-1-Pyrroline-5-carboxylate
Pyrrole-2-carboxylate
2,5-Dioxo-pentanoate
1-Pyrroline-4-hydroxy-2-carboxylate
2-Oxo-4-hydroxy-5-aminovalerate
cis-4-Hydroxy-D-proline
4-Hydroxy-L-proline
4-Oxoproline
L-1-Pyrroline-3-hydroxy-5-carboxylate
L-erythro-4-Hydroxy-glutamate
4-Hydroxy-2-oxoglutarate
Pyruvate
Glyoxylate
L-4-Hydroxyglutamate semialdehyde
N2-Succinylglutamate
N2-Succinyl-L-glutamate 5-semialdehyde
N2-Succinyl-L-ornithine
N2-Succinyl-L-arginine
2-Oxo-arginine
4-Guanidino-butanal
4-Guanidino-butanamide
N-Carbamoyl-putrescine
Agmatine
Putrescine
Glutamate
γ-L-Glutamyl-putrescine
γ-Glutamyl-γ-amino-butyraldehyde
γ-Glutamyl-γ-amino-butyrate
N-Acetyl-putrescine
N4-Acetyl-aminobutanal
N4-Acetyl-amino-butanoate
L-Aspartate-4-semialdehyde
1,3-Diamino-propane
Carboxy-norspermidine
Carboxy-spermidine
Norspermidine
Spermidine
Spermine
S-Adenosyl-L-methionine
S-Adenosyl-methioninamine
p-Coumaroyl-CoA
p-Coumaroyl-ptrescine
p-Coumaroyl-agmatine
Feruloyl-CoA
Feruloylptrescine
Feruloylagmatine
4-Amino-butanal
4-Amino-butanoate
L-Homocarnosine
4-Guanidino-butanoate
PuuA
PuuB
PuuC
PuuD
ACT
PRODH
PRODH2
3.5.1.2
3.5.1.38
6.3.1.2
2.7.2.2
6.3.4.16
1.4.1.2
1.4.1.3
1.4.1.4
2.1.3.11
2.1.3.9
2.1.3.3
3.5.1.16
3.5.3.6
1.14.13.39
6.3.4.5
4.3.2.1
3.5.3.1
3.5.1.14
3.5.1.16
2.6.1.11
2.3.1.35
2.3.1.1
2.7.2.8
1.2.1.38
2.7.3.1
2.1.4.1
2.1.1.2
2.7.3.2
3.5.2.10
3.5.3.3
3.5.4.1
3.5.4.21
3.5.1.59
3.5.2.14
1.5.1.19
1.5.1.11
2.7.3.3
1.14.13.39
6.3.4.6
3.5.1.54
3.5.1.5
2.3.1.109
4.1.1.19
2.6.1.84
1.13.12.1
2.6.1.13
4.3.1.12
1.2.1.88
2.7.2.11
1.2.1.41
3.4.11.5
1.5.1.1
1.4.3.3
1.5.99.13
1.4.1.12
1.2.1.41
1.5.1.2
5.1.1.4
1.5.5.2
1.14.11.2
1.5.1.2
1.4.3.3
3.5.4.22
5.1.1.8
1.1.1.104
1.2.1.88
2.6.1.1
2.6.1.21
2.6.1.23
4.1.3.16
4.1.3.42
4.1.1.17
3.5.1.96
1.2.1.71
2.6.1.81
3.5.3.23
4.1.1.75
3.5.1.53
3.5.3.12
3.5.3.11
2.3.1.57
3.5.1.62
1.4.3.4
1.2.1.3
3.5.1.63
1.4.3.10
2.6.1.29
1.4.3.22
2.6.1.82
2.6.1.-
1.2.1.3
1.2.1.19
1.2.1.-
1.5.1.43
4.1.1.-
1.5.3.14
1.5.99.6
1.5.3.17
4.1.1.50
2.5.1.16
2.5.1.22
1.5.3.16
3.4.13.18
3.4.13.20
6.3.2.11
2.3.1.64
1.2.1.54
3.5.1.4
3.5.3.7
2.1.4.1
00330 3/14/14
(c) Kanehisa Laboratories

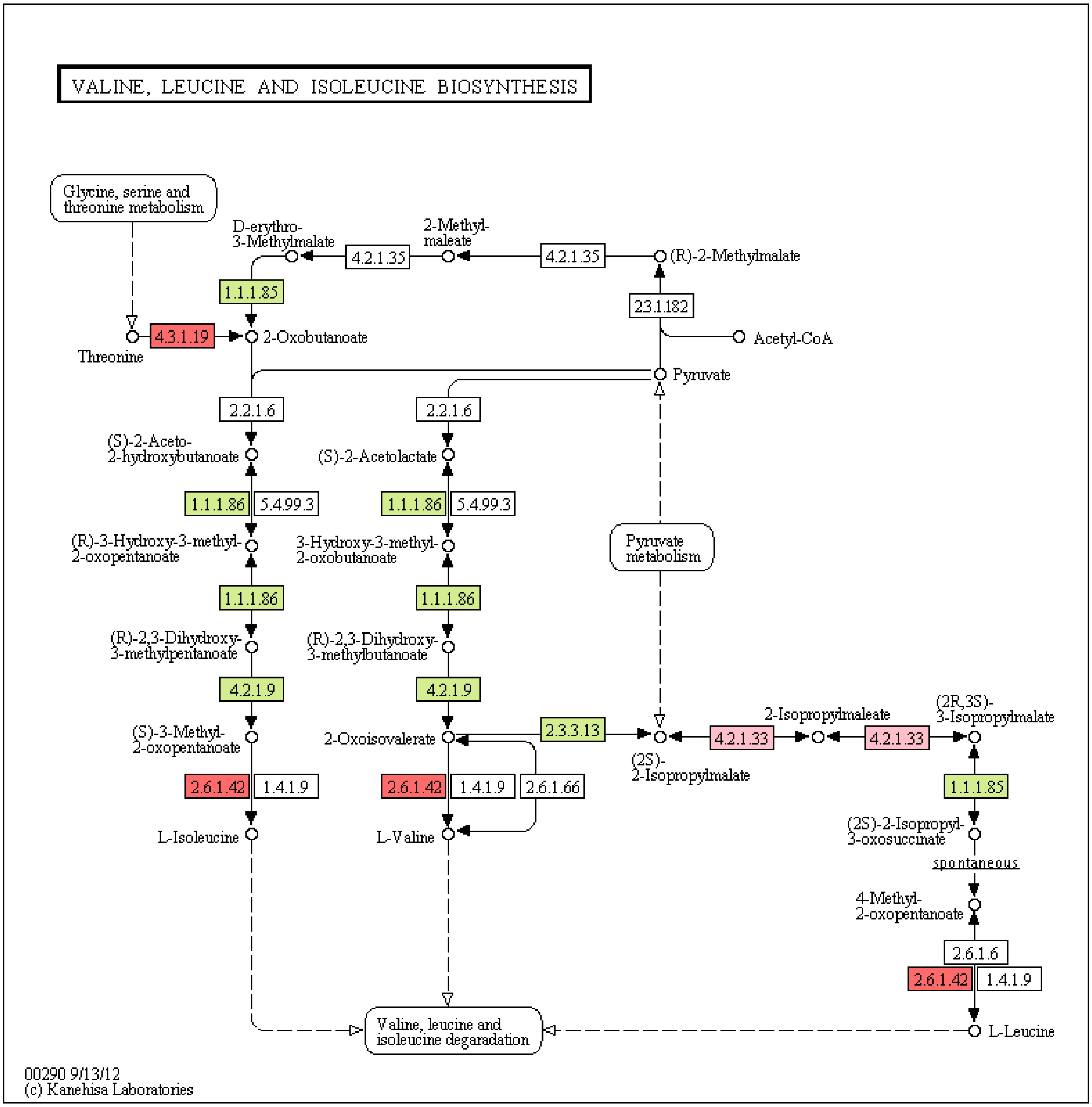
VALINE, LEUCINE AND ISOLEUCINE BIOSYNTHESIS
Glycine, serine and threonine metabolism
D-erythro-3-Methylmalate
2-Methylmaleate
4.2.1.35
4.2.1.35
(R)-2-Methylmalate
1.1.1.85
2.3.1.182
Threonine
4.3.1.19
2-Oxobutanoate
Acetyl-CoA
Pyruvate
2.2.1.6
2.2.1.6
(S)-2-Aceto-2-hydroxybutanoate
(S)-2-Acetolactate
1.1.1.86
5.4.99.3
1.1.1.86
5.4.99.3
(R)-3-Hydroxy-3-methyl-2-oxopentanoate
3-Hydroxy-3-methyl-2-oxobutanoate
Pyruvate metabolism
1.1.1.86
1.1.1.86
(R)-2,3-Dihydroxy-3-methylpentanoate
(R)-2,3-Dihydroxy-3-methylbutanoate
4.2.1.9
4.2.1.9
(S)-3-Methyl-2-oxopentanoate
2-Oxoisovalerate
2.3.3.13
(2S)-2-Isopropylmalate
4.2.1.33
2-Isopropylmaleate
4.2.1.33
(2R,3S)-3-Isopropylmalate
2.6.1.42
1.4.1.9
2.6.1.42
1.4.1.9
2.6.1.66
1.1.1.85
L-Isoleucine
L-Valine
(2S)-2-Isopropyl-3-oxosuccinate
spontaneous
4-Methyl-2-oxopentanoate
2.6.1.6
2.6.1.42
1.4.1.9
Valine, leucine and isoleucine degaradation
L-Leucine
00290 9/13/12
(c) Kanehisa Laboratories

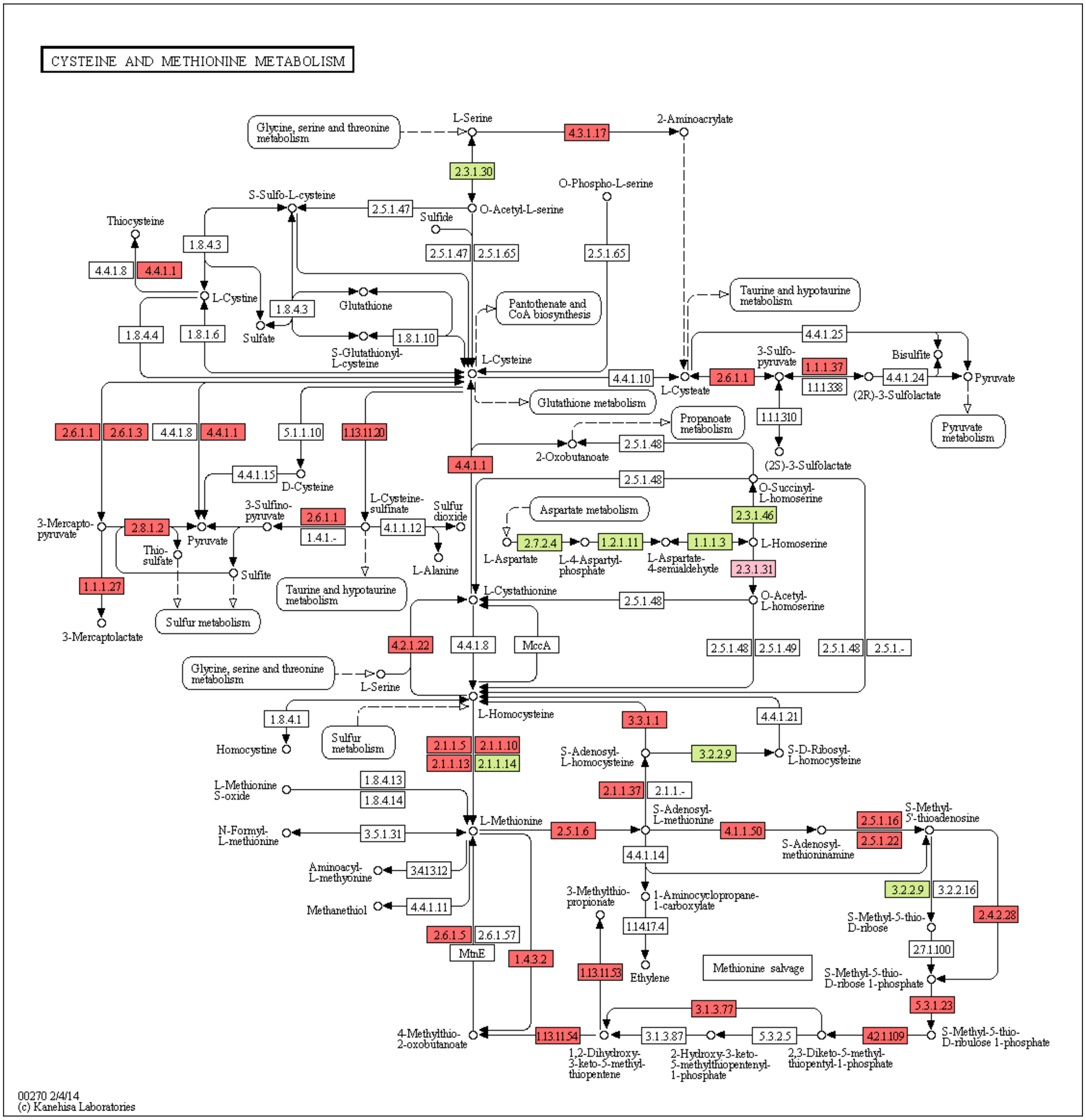
CYSTEINE AND METHIONINE METABOLISM
Glycine, serine and threonine metabolism
L-Serine
4.3.1.17
2-Aminoacrylate
2.3.1.30
O-Phospho-L-serine
S-Sulfo-L-cysteine
2.5.1.47
O-Acetyl-L-serine
Sulfide
Thiocysteine
1.8.4.3
2.5.1.47
2.5.1.65
2.5.1.65
4.4.1.8
4.4.1.1
L-Cystine
Glutathione
Pantothenate and CoA biosynthesis
Taurine and hypotaurine metabolism
1.8.4.3
Sulfate
1.8.4.4
1.8.1.6
1.8.1.10
S-Glutathionyl-L-cysteine
L-Cysteine
4.4.1.10
L-Cysteate
2.6.1.1
3-Sulfo-pyruvate
1.1.1.37
1.1.1.338
4.4.1.25
Bisulfite
4.4.1.24
(2R)-3-Sulfolactate
Pyruvate
Pyruvate metabolism
Glutathione metabolism
Propanoate metabolism
1.1.1.310
2.6.1.1
2.6.1.3
4.4.1.8
4.4.1.1
5.1.1.10
1.13.11.20
2.5.1.48
2-Oxobutanoate
4.4.1.1
(2S)-3-Sulfolactate
4.4.1.15
D-Cysteine
2.5.1.48
O-Succinyl-L-homoserine
3-Mercapto-pyruvate
2.8.1.2
Pyruvate
3-Sulfino-pyruvate
2.6.1.1
1.4.1.-
L-Cysteine-sulfinate
4.1.1.12
Sulfur dioxide
Aspartate metabolism
2.3.1.46
Thio-sulfate
L-Alanine
2.7.2.4
1.2.1.11
1.1.1.3
L-Homoserine
L-Aspartate
L-4-Aspartyl-phosphate
L-Aspartate-4-semialdehyde
2.3.1.31
Sulfite
1.1.1.27
Taurine and hypotaurine metabolism
L-Cystathionine
2.5.1.48
O-Acetyl-L-homoserine
Sulfur metabolism
3-Mercaptolactate
4.2.1.22
4.4.1.8
MccA
2.5.1.48
2.5.1.49
2.5.1.48
2.5.1.-
Glycine, serine and threonine metabolism
L-Serine
L-Homocysteine
1.8.4.1
Sulfur metabolism
3.3.1.1
4.4.1.21
Homocystine
2.1.1.5
2.1.1.10
S-Adenosyl-L-homocysteine
3.2.2.9
S-D-Ribosyl-L-homocysteine
2.1.1.13
2.1.1.14
L-Methionine S-oxide
1.8.4.13
1.8.4.14
2.1.1.37
2.1.1.-
S-Adenosyl-L-methionine
S-Methyl-5'-thioadenosine
L-Methionine
2.5.1.6
4.1.1.50
2.5.1.16
N-Formyl-L-methionine
3.5.1.31
S-Adenosyl-methioninamine
2.5.1.22
4.4.1.14
Aminoacyl-L-methionine
3.4.13.12
3.2.2.9
3.2.2.16
3-Methylthio-propionate
1-Aminocyclopropane-1-carboxylate
Methanethiol
4.4.1.11
S-Methyl-5-thio-D-ribose
2.4.2.28
1.14.17.4
2.7.1.100
2.6.1.5
2.6.1.57
MtnE
1.4.3.2
1.13.11.53
Ethylene
Methionine salvage
S-Methyl-5-thio-D-ribose 1-phosphate
3.1.3.77
5.3.1.23
4-Methylthio-2-oxobutanoate
1.13.11.54
3.1.3.87
5.3.2.5
4.2.1.109
S-Methyl-5-thio-D-ribulose 1-phosphate
1,2-Dihydroxy-3-keto-5-methyl-thiopentene
2-Hydroxy-3-keto-5-methylthiopentenyl-1-phosphate
2,3-Diketo-5-methyl-thiopentyl-1-phosphate
00270 2/4/14
(c) Kanehisa Laboratories

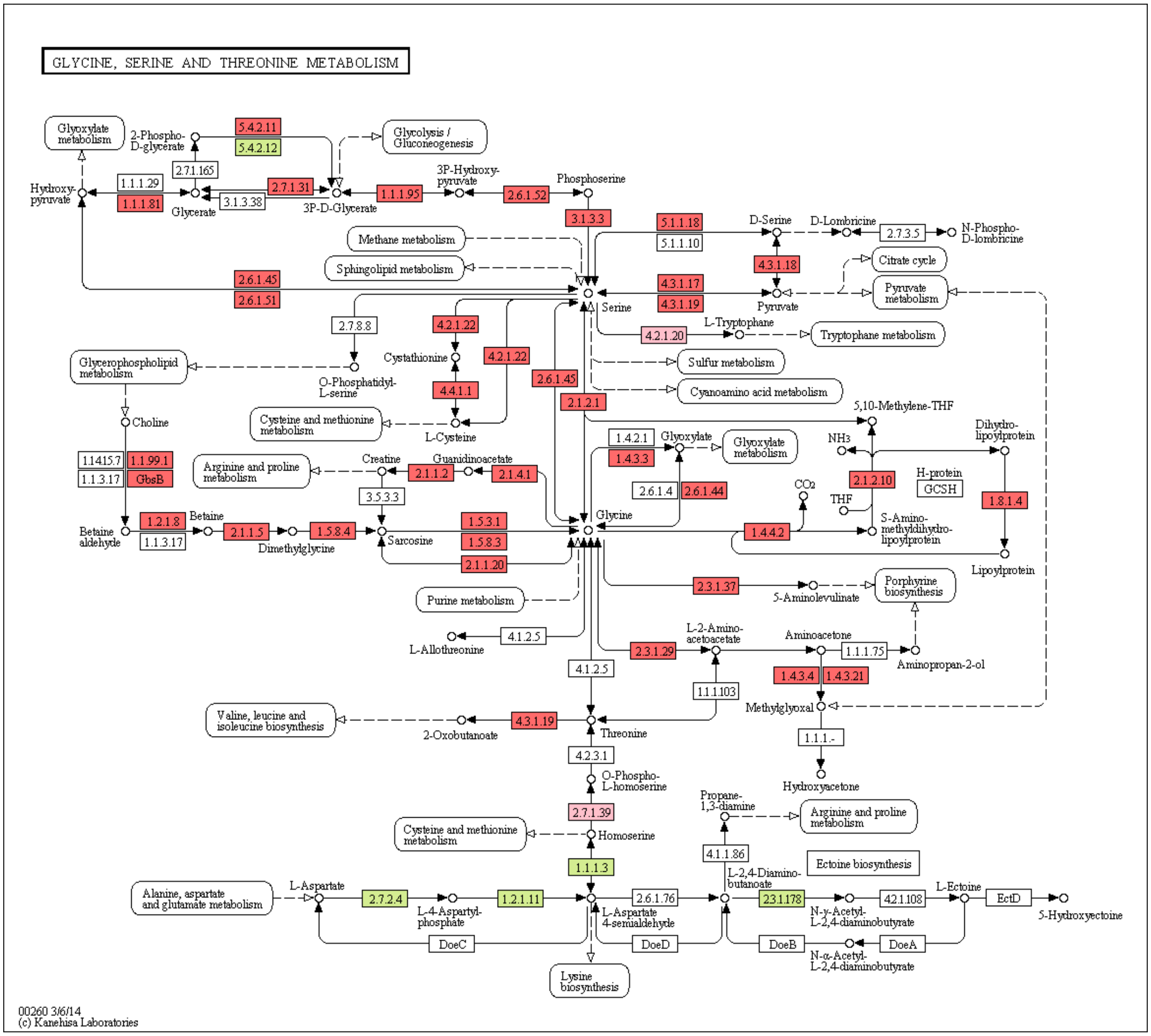
GLYCINE, SERINE AND THREONINE METABOLISM
Glyoxylate metabolism
2-Phospho-D-glycerate
5.4.2.11
5.4.2.12
Glycolysis / Gluconeogenesis
2.7.1.165
1.1.1.29
Hydroxy-pyruvate
1.1.1.81
Glycerate
3.1.3.38
2.7.1.31
3P-D-Glycerate
1.1.1.95
3P-Hydroxy-pyruvate
2.6.1.52
Phosphoserine
3.1.3.3
5.1.1.18
5.1.1.10
D-Serine
D-Lombricine
2.7.3.5
N-Phospho-D-lombricine
Methane metabolism
Sphingolipid metabolism
4.3.1.18
Citrate cycle
2.6.1.45
2.6.1.51
4.3.1.17
4.3.1.19
Pyruvate
Pyruvate metabolism
Serine
2.7.8.8
4.2.1.22
4.2.1.22
4.2.1.20
L-Tryptophane
Tryptophane metabolism
Glycerophospholipid metabolism
O-Phosphatidyl-L-serine
Cystathionine
2.6.1.45
Sulfur metabolism
4.4.1.1
Cyanoamino acid metabolism
2.1.2.1
Choline
Cysteine and methionine metabolism
L-Cysteine
5,10-Methylene-THF
1.4.2.1
Glyoxylate
Glyoxylate metabolism
NH3
Dihydro-lipoylprotein
1.4.3.3
1.1.4.15.7
1.1.99.1
1.1.3.17
GbsB
Arginine and proline metabolism
Creatine
Guanidinoacetate
2.1.1.2
2.1.4.1
3.5.3.3
2.6.1.4
2.6.1.44
H-protein
GCSH
2.1.2.10
CO2
THF
1.8.1.4
1.2.1.8
Betaine
Betaine aldehyde
1.1.3.17
2.1.1.5
1.5.8.4
Dimethylglycine
Sarcosine
1.5.3.1
1.5.8.3
Glycine
1.4.4.2
S-Amino-methyldihydro-lipoylprotein
Lipoylprotein
2.1.1.20
2.3.1.37
5-Aminolevulinate
Porphyrine biosynthesis
Purine metabolism
L-Allothreonine
4.1.2.5
4.1.2.5
2.3.1.29
L-2-Amino-acetoacetate
Aminoacetone
1.1.1.75
Aminopropan-2-ol
1.1.1.103
1.4.3.4
1.4.3.21
Valine, leucine and isoleucine biosynthesis
2-Oxobutanoate
4.3.1.19
Threonine
Methylglyoxal
1.1.1.-
4.2.3.1
Hydroxyacetone
O-Phospho-L-homoserine
Propane-1,3-diamine
Arginine and proline metabolism
2.7.1.39
Cysteine and methionine metabolism
Homoserine
4.1.1.86
Ectoine biosynthesis
1.1.1.3
L-2,4-Diamino-butanoate
Alanine, aspartate and glutamate metabolism
L-Aspartate
2.7.2.4
L-4-Aspartyl-phosphate
1.2.1.11
2.6.1.76
L-Aspartate 4-semialdehyde
2.3.1.178
N-γ-Acetyl-L-2,4-diaminobutyrate
4.2.1.108
L-Ectoine
EctD
5-Hydroxyectoine
DoeC
DoeD
DoeB
DoeA
N-α-Acetyl-L-2,4-diaminobutyrate
Lysine biosynthesis
00260 3/6/14
(c) Kanehisa Laboratories

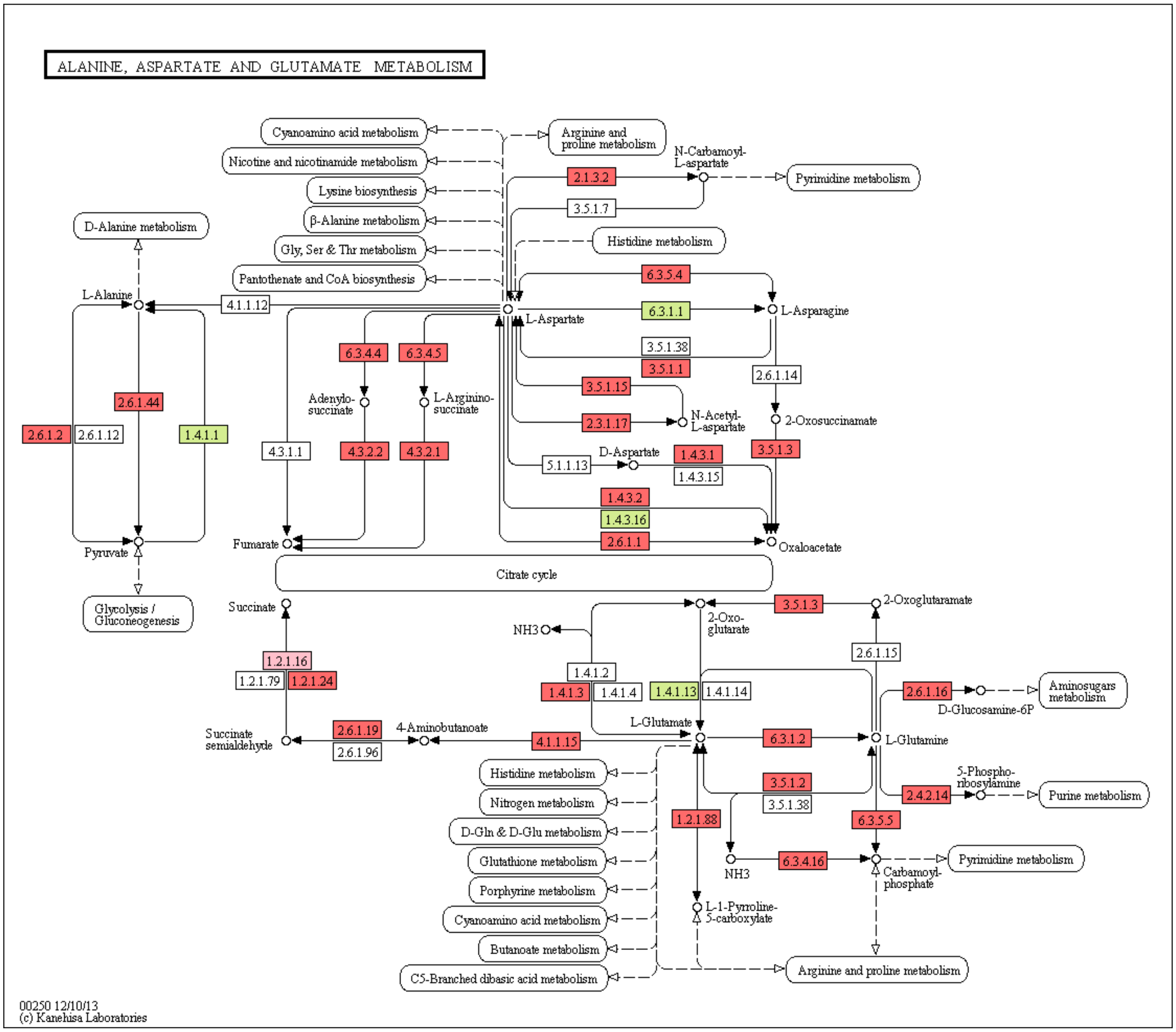
ALANINE, ASPARTATE AND GLUTAMATE METABOLISM
Cyanoamino acid metabolism
Nicotine and nicotinamide metabolism
Lysine biosynthesis
β-Alanine metabolism
Gly, Ser & Thr metabolism
Pantothenate and CoA biosynthesis
Arginine and proline metabolism
N-Carbamoyl-L-aspartate
Pyrimidine metabolism
2.1.3.2
3.5.1.7
Histidine metabolism
6.3.5.4
D-Alanine metabolism
L-Alanine
4.1.1.12
L-Aspartate
6.3.1.1
L-Asparagine
6.3.4.4
6.3.4.5
3.5.1.38
3.5.1.1
2.6.1.14
2.6.1.44
Adenylo-succinate
L-Argininosuccinate
3.5.1.15
N-Acetyl-L-aspartate
2.3.1.17
2-Oxosuccinamate
2.6.1.2
2.6.1.12
1.4.1.1
4.3.1.1
4.3.2.2
4.3.2.1
D-Aspartate
1.4.3.1
3.5.1.3
5.1.1.13
1.4.3.15
1.4.3.2
1.4.3.16
2.6.1.1
Pyruvate
Fumarate
Oxaloacetate
Citrate cycle
Glycolysis / Gluconeogenesis
Succinate
2-Oxo-glutarate
3.5.1.3
2-Oxoglutaramate
NH3
1.2.1.16
1.2.1.79
1.2.1.24
2.6.1.15
1.4.1.2
1.4.1.3
1.4.1.4
1.4.1.13
1.4.1.14
2.6.1.16
D-Glucosamine-6P
Aminosugars metabolism
Succinate semialdehyde
2.6.1.19
2.6.1.96
4-Aminobutanoate
4.1.1.15
L-Glutamate
6.3.1.2
L-Glutamine
Histidine metabolism
Nitrogen metabolism
D-Gln & D-Glu metabolism
Glutathione metabolism
Porphyrine metabolism
Cyanoamino acid metabolism
Butanoate metabolism
C5-Branched dibasic acid metabolism
3.5.1.2
3.5.1.38
5-Phospho-ribosylamine
2.4.2.14
Purine metabolism
1.2.1.88
6.3.5.5
NH3
6.3.4.16
Carbamoyl-phosphate
Pyrimidine metabolism
L-1-Pyrroline-5-carboxylate
Arginine and proline metabolism
00250 12/10/13
(c) Kanehisa Laboratories

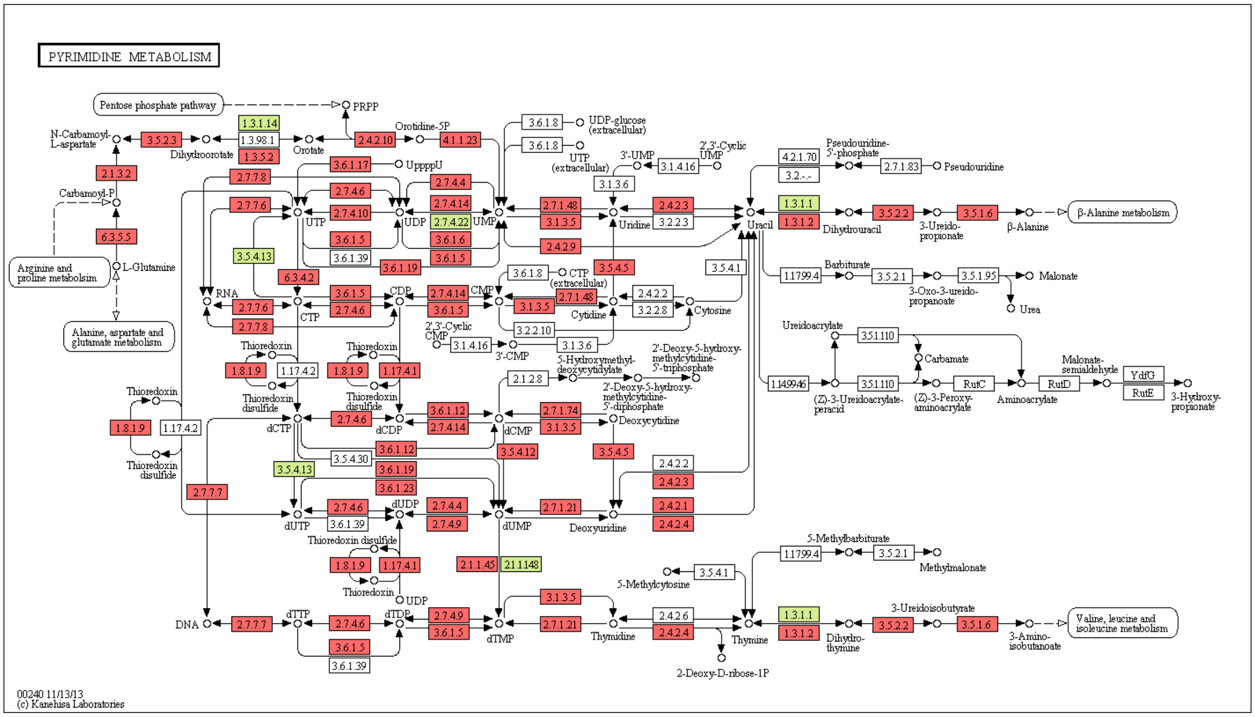
PYRIMIDINE METABOLISM
Pentose phosphate pathway
PRPP
N-Carbamoyl-L-aspartate
3.5.2.3
1.3.1.14
1.3.98.1
1.3.5.2
Dihydroorotate
Orotate
2.4.2.10
Orotidine-5P
4.1.1.23
3.6.1.8
UDP-glucose (extracellular)
UTP (extracellular)
3'-UMP
2',3'-Cyclic UMP
3.1.4.16
3.1.3.6
Pseudouridine-5'-phosphate
4.2.1.70
3.2.-.-
2.7.1.83
Pseudouridine
2.1.3.2
Carbamoyl-P
6.3.5.5
L-Glutamine
Arginine and proline metabolsim
Alanine, aspartate and glutamate metabolism
3.6.1.17
UpppppU
2.7.7.8
2.7.7.6
2.7.4.6
2.7.4.10
2.7.4.4
2.7.4.14
2.7.4.22
3.6.1.5
3.6.1.6
3.6.1.39
3.6.1.19
3.5.4.13
6.3.4.2
UTP
UDP
UMP
2.7.1.48
3.1.3.5
2.4.2.9
2.4.2.3
3.2.2.3
Uridine
Uracil
1.3.1.1
1.3.1.2
Dihydrouracil
3.5.2.2
3-Ureido-propionate
3.5.1.6
β-Alanine
β-Alanine metabolism
3.5.4.1
1.17.99.4
Barbiturate
3.5.2.1
3-Oxo-3-ureido-propanoate
3.5.1.95
Malonate
Urea
RNA
CTP
CDP
CMP
CTP (extracellular)
3.5.4.5
2.4.2.2
3.2.2.8
Cytidine
Cytosine
2',3'-Cyclic CMP
3.1.4.16
3'-CMP
3.2.2.10
Ureidoacrylate
3.5.1.110
Carbamate
1.14.99.46
(Z)-3-Ureidoacrylate-peracid
(Z)-3-Peroxy-aminoacrylate
RutC
Aminoacrylate
RutD
Malonate-semialdehyde
YdfG
RutE
3-Hydroxy-propionate
Thioredoxin
Thioredoxin disulfide
1.8.1.9
1.17.4.2
1.17.4.1
dCTP
dCDP
dCMP
3.6.1.12
2.7.1.74
3.5.4.12
3.5.4.30
5-Hydroxymethyl-deoxycytidylate
2.1.2.8
2'-Deoxy-5-hydroxy-methylcytidine-5'-triphosphate
2'-Deoxy-5-hydroxy-methylcytidine-5'-diphosphate
Deoxycytidine
2.7.7.7
3.6.1.23
dUTP
dUDP
dUMP
2.7.4.9
2.7.1.21
2.4.2.1
2.4.2.4
Deoxyuridine
2.1.1.45
2.1.1.148
5-Methylbarbiturate
Methylmalonate
5-Methylcytosine
DNA
dTTP
dTDP
dTMP
UDP
Thymidine
2.4.2.6
Thymine
Dihydro-thymine
3-Ureidoisobutyrate
3-Amino-isobutanoate
Valine, leucine and isoleucine metabolism
2-Deoxy-D-ribose-1P
00240 11/13/13
(c) Kanehisa Laboratories

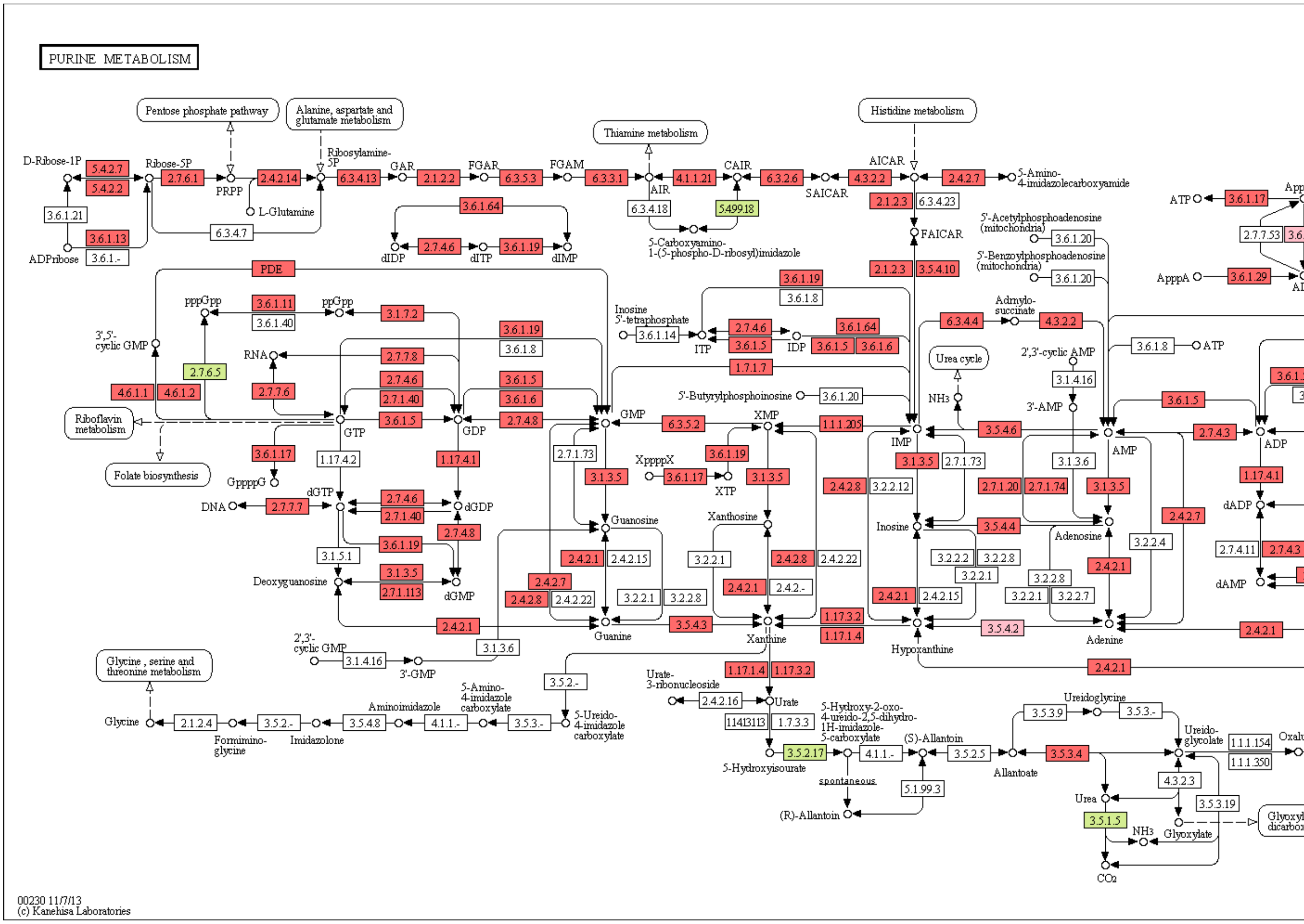
PURINE METABOLISM
Pentose phosphate pathway
Alanine, aspartate and glutamate metabolism
Thiamine metabolism
Histidine metabolism
D-Ribose-1P
5.4.2.7
5.4.2.2
Ribose-5P
2.7.6.1
PRPP
2.4.2.14
Ribosylamine-5P
6.3.4.13
GAR
2.1.2.2
FGAR
6.3.5.3
FGAM
6.3.3.1
AIR
4.1.1.21
CAIR
6.3.2.6
SAICAR
4.3.2.2
AICAR
2.4.2.7
5-Amino-4-imidazolecarboxyamide
3.6.1.21
3.6.1.13
ADPribose
3.6.1.-
L-Glutamine
6.3.4.7
3.6.1.64
dIDP
2.7.4.6
dITP
3.6.1.19
dIMP
6.3.4.18
5.4.99.18
5-Carboxyamino-1-(5-phospho-D-ribosyl)imidazole
2.1.2.3
6.3.4.23
FAICAR
2.1.2.3
3.5.4.10
ATP
3.6.1.17
5'-Acetylphosphoadenosine (mitochondria)
3.6.1.20
5'-Benzoylphosphoadenosine (mitochondria)
3.6.1.20
2.7.7.53
ApppA
3.6.1.29
PDE
pppGpp
3.6.1.11
3.6.1.40
ppGpp
3.1.7.2
3',5'-cyclic GMP
RNA
2.7.7.6
2.7.6.5
4.6.1.1
4.6.1.2
2.7.7.6
Riboflavin metabolism
Folate biosynthesis
GTP
2.7.4.6
2.7.1.40
3.6.1.5
3.6.1.19
3.6.1.8
3.6.1.5
3.6.1.6
GDP
2.7.4.8
GMP
3.6.1.17
GppppG
1.17.4.2
1.17.4.1
Inosine 5'-tetraphosphate
3.6.1.14
ITP
2.7.4.6
3.6.1.5
IDP
3.6.1.64
3.6.1.5
3.6.1.6
3.6.1.19
3.6.1.8
1.7.1.7
6.3.4.4
Adenylo-succinate
4.3.2.2
3.6.1.8
ATP
2',3'-cyclic AMP
Urea cycle
NH3
3.1.4.16
3'-AMP
5'-Butyrylphosphoinosine
3.6.1.20
6.3.5.2
XMP
1.1.1.205
IMP
3.5.4.6
AMP
3.6.1.5
2.7.4.3
ADP
2.7.1.73
XppppX
3.6.1.17
XTP
3.6.1.19
3.1.3.5
3.1.3.5
3.1.3.5
2.7.1.73
3.1.3.6
2.7.1.20
2.7.1.74
3.1.3.5
1.17.4.1
dGTP
DNA
2.7.7.7
2.7.4.6
2.7.1.40
dGDP
2.7.4.8
3.6.1.19
3.1.5.1
3.1.3.5
2.7.1.113
Deoxyguanosine
dGMP
2.4.2.1
2.4.2.15
Guanosine
Xanthosine
3.2.2.1
2.4.2.8
2.4.2.22
2.4.2.8
3.2.2.12
Inosine
3.5.4.4
Adenosine
3.2.2.4
2.4.2.7
dADP
2.7.4.11
2.7.4.3
3.2.2.2
3.2.2.8
3.2.2.1
2.4.2.7
2.4.2.8
2.4.2.22
3.2.2.1
3.2.2.8
2.4.2.1
2.4.2.-
2.4.2.1
2.4.2.15
3.2.2.8
3.2.2.1
3.2.2.7
2.4.2.1
dAMP
Guanine
3.5.4.3
Xanthine
1.17.3.2
1.17.1.4
Hypoxanthine
3.5.4.2
Adenine
2.4.2.1
2.4.2.1
2',3'-cyclic GMP
3.1.4.16
3'-GMP
3.1.3.6
2.4.2.1
Glycine, serine and threonine metabolism
Glycine
2.1.2.4
Formimino-glycine
3.5.2.-
Imidazolone
3.5.4.8
Aminoimidazole
4.1.1.-
5-Amino-4-imidazole carboxylate
3.5.3.-
3.5.2.-
5-Ureido-4-imidazole carboxylate
1.17.1.4
1.17.3.2
Urate-3-ribonucleoside
2.4.2.16
Urate
1.14.13.113
1.7.3.3
5-Hydroxyisourate
3.5.2.17
5-Hydroxy-2-oxo-4-ureido-2,5-dihydro-1H-imidazole-5-carboxylate
4.1.1.-
(S)-Allantoin
spontaneous
5.1.99.3
(R)-Allantoin
3.5.2.5
Allantoate
3.5.3.4
Ureidoglycine
3.5.3.9
3.5.3.-
Ureido-glycolate
1.1.1.154
1.1.1.350
4.3.2.3
Urea
3.5.3.19
3.5.1.5
NH3
Glyoxylate
CO2
00230 11/7/13
(c) Kanehisa Laboratories

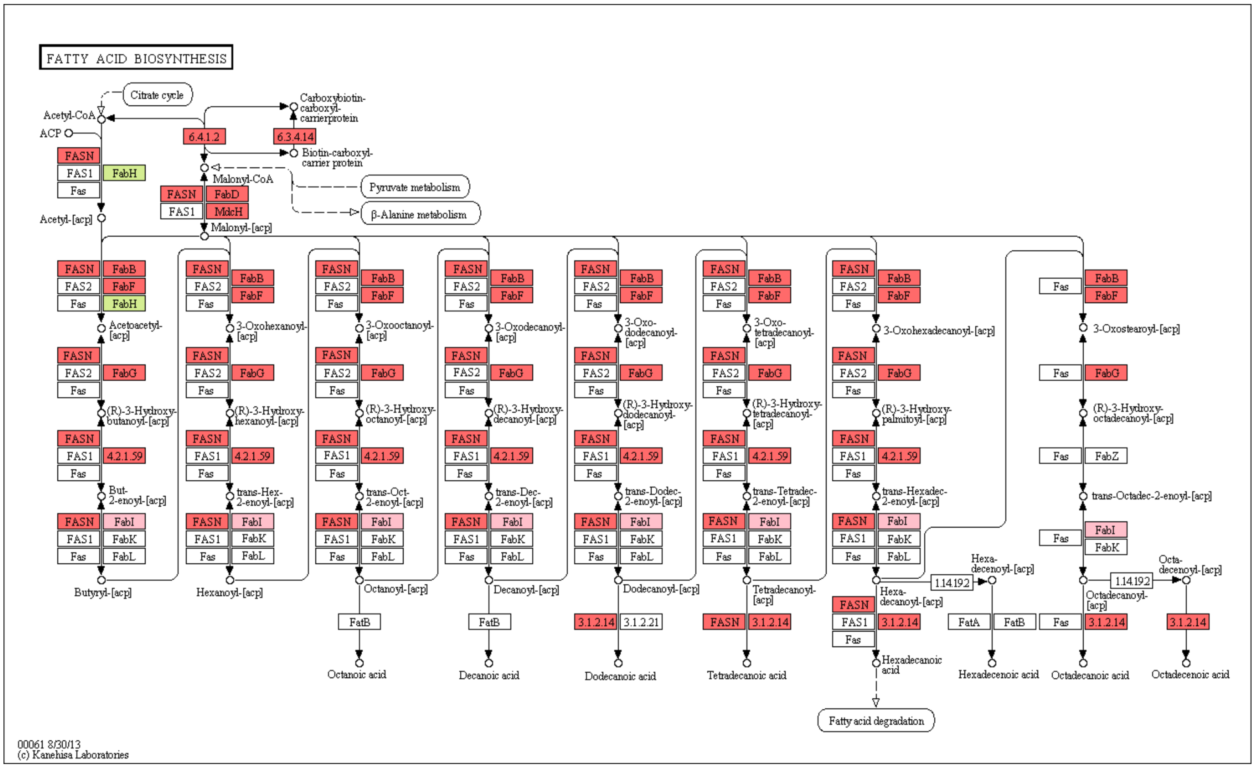

(c) Kanehisa Laboratories

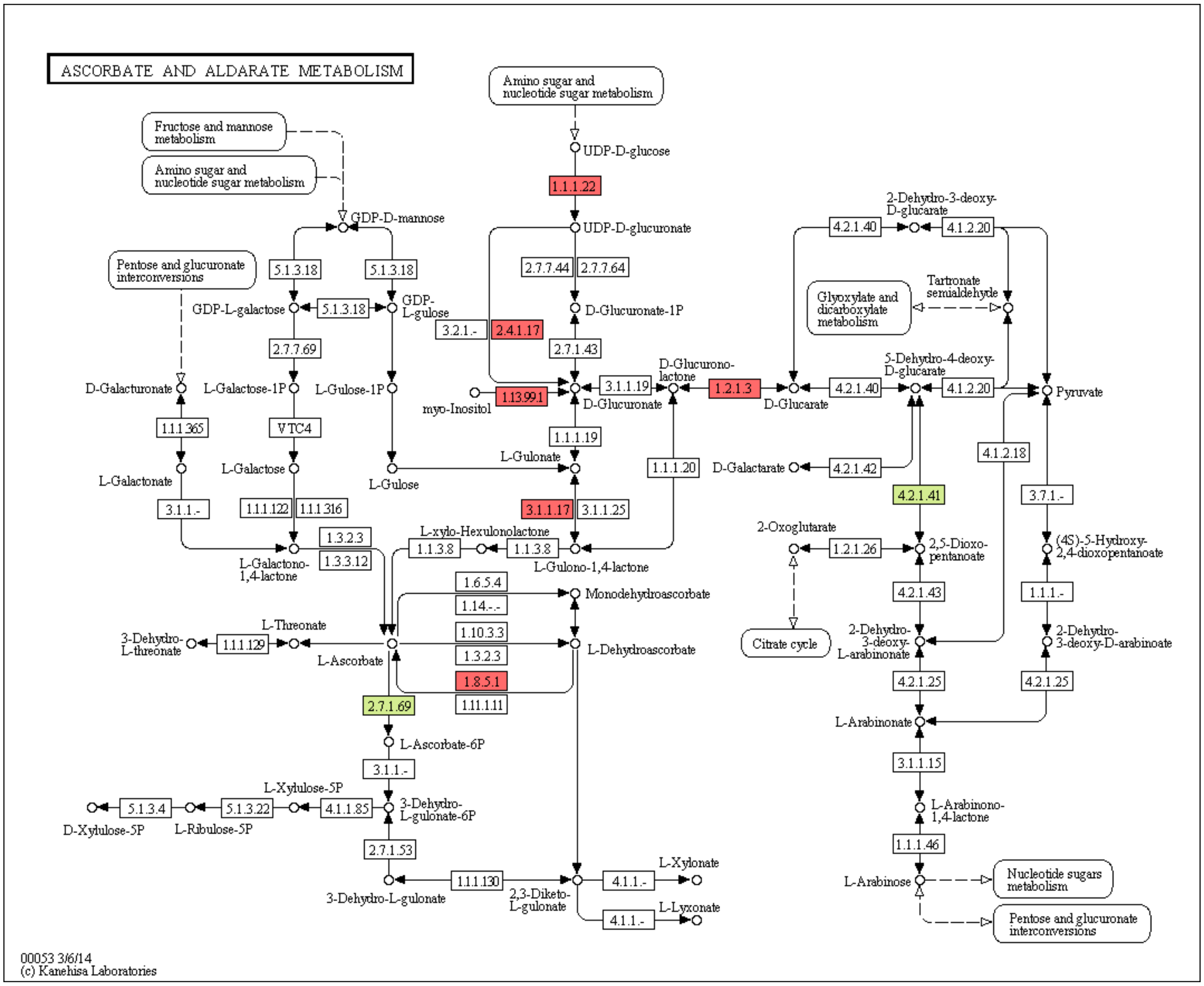
ASCORBATE AND ALDARATE METABOLISM
Amino sugar and nucleotide sugar metabolism
Fructose and mannose metabolism
Amino sugar and nucleotide sugar metabolism
UDP-D-glucose
1.1.1.22
GDP-D-mannose
UDP-D-glucuronate
2-Dehydro-3-deoxy-D-glucarate
4.2.1.40
4.1.2.20
Pentose and glucuronate interconversions
5.1.3.18
5.1.3.18
2.7.7.44
2.7.7.64
Glyoxylate and dicarboxylate metabolism
Tartronate semialdehyde
GDP-L-galactose
5.1.3.18
GDP-L-gulose
D-Glucuronate-1P
3.2.1.-
2.4.1.17
2.7.7.69
2.7.1.43
D-Glucurono-lactone
5-Dehydro-4-deoxy-D-glucarate
D-Galacturonate
L-Galactose-1P
L-Gulose-1P
3.1.1.19
1.2.1.3
4.2.1.40
4.1.2.20
Pyruvate
myo-Inositol
1.13.99.1
D-Glucuronate
D-Glucarate
1.1.1.365
VTC4
1.1.1.19
L-Gulonate
4.1.2.18
L-Galactonate
L-Galactose
L-Gulose
1.1.1.20
D-Galactarate
4.2.1.42
4.2.1.41
3.7.1.-
3.1.1.-
1.1.1.122
1.1.1.316
3.1.1.17
3.1.1.25
1.3.2.3
L-xylo-Hexulonolactone
2-Oxoglutarate
1.3.3.12
1.1.3.8
1.1.3.8
1.2.1.26
2,5-Dioxo-pentanoate
(4S)-5-Hydroxy-2,4-dioxopentanoate
L-Galactono-1,4-lactone
L-Gulono-1,4-lactone
1.6.5.4
Monodehydroascorbate
4.2.1.43
1.1.1.-
1.14.-.-
L-Threonate
3-Dehydro-L-threonate
1.1.1.129
1.10.3.3
L-Dehydroascorbate
Citrate cycle
2-Dehydro-3-deoxy-L-arabinonate
2-Dehydro-3-deoxy-D-arabinoate
L-Ascorbate
1.3.2.3
1.8.5.1
4.2.1.25
4.2.1.25
2.7.1.69
1.11.1.11
L-Arabinonate
L-Ascorbate-6P
3.1.1.-
3.1.1.15
L-Xylulose-5P
L-Arabinono-1,4-lactone
5.1.3.4
5.1.3.22
4.1.1.85
3-Dehydro-L-gulonate-6P
D-Xylulose-5P
L-Ribulose-5P
2.7.1.53
1.1.1.46
1.1.1.130
4.1.1.-
L-Xylonate
L-Arabinose
Nucleotide sugars metabolism
3-Dehydro-L-gulonate
2,3-Diketo-L-gulonate
4.1.1.-
L-Lyxonate
Pentose and glucuronate interconversions
00053 3/6/14
(c) Kanehisa Laboratories

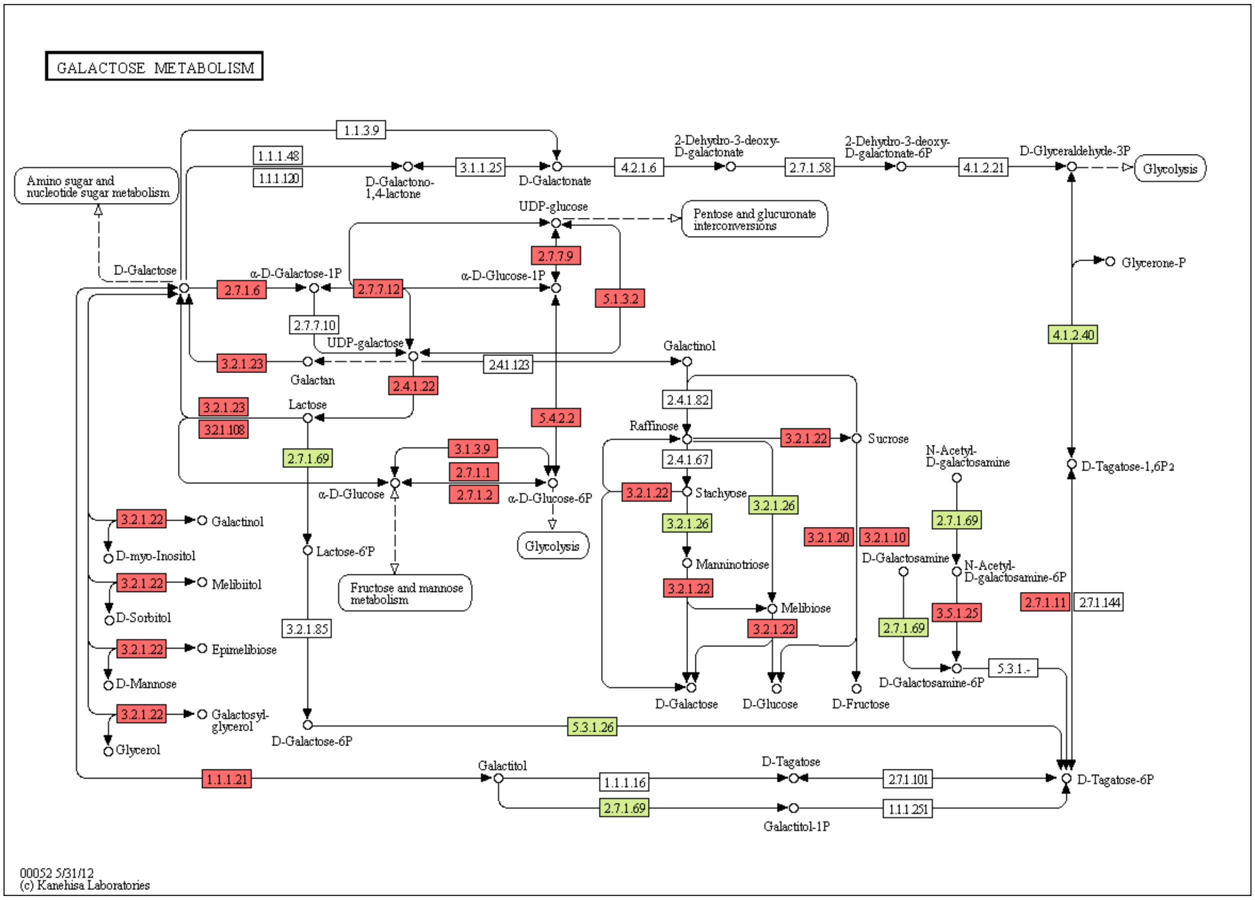
GALACTOSE METABOLISM
1.1.3.9
1.1.1.48
1.1.1.120
3.1.1.25
4.2.1.6
2.7.1.58
4.1.2.21
2-Dehydro-3-deoxy-
D-galactonate
2-Dehydro-3-deoxy-
D-galactonate-6P
D-Glyceraldehyde-3P
Glycolysis
Amino sugar and
nucleotide sugar metabolism
D-Galactono-
1,4-lactone
D-Galactonate
UDP-glucose
Pentose and glucuronate
interconversions
2.7.7.9
5.1.3.2
D-Galactose
α-D-Galactose-1P
2.7.1.6
2.7.7.12
α-D-Glucose-1P
Glycerone-P
4.1.2.40
2.7.7.10
UDP-galactose
3.2.1.23
Galactan
2.4.1.22
2.4.1.123
Galactinol
2.4.1.82
3.2.1.23
3.2.1.108
Lactose
5.4.2.2
Raffinose
3.2.1.22
Sucrose
2.7.1.69
3.1.3.9
2.7.1.1
2.7.1.2
2.4.1.67
N-Acetyl-
D-galactosamine
D-Tagatose-1,6P2
α-D-Glucose
α-D-Glucose-6P
3.2.1.22
Stachyose
3.2.1.26
3.2.1.22
Galactinol
3.2.1.26
2.7.1.69
D-myo-Inositol
Lactose-6'P
Glycolysis
3.2.1.20
3.2.1.10
Manninotriose
D-Galactosamine
N-Acetyl-
D-galactosamine-6P
3.2.1.22
Melibiitol
Fructose and mannose
metabolism
3.2.1.22
Melibiose
3.5.1.25
2.7.1.11
2.7.1.144
D-Sorbitol
3.2.1.85
3.2.1.22
2.7.1.69
3.2.1.22
Epimelibiose
5.3.1.-
D-Mannose
D-Galactose
D-Glucose
D-Fructose
D-Galactosamine-6P
3.2.1.22
Galactosyl-
glycerol
5.3.1.26
Glycerol
D-Galactose-6P
Galactitol
D-Tagatose
1.1.1.21
1.1.1.16
2.7.1.101
D-Tagatose-6P
2.7.1.69
Galactitol-1P
1.1.1.251
00052 5/31/12
(c) Kanehisa Laboratories

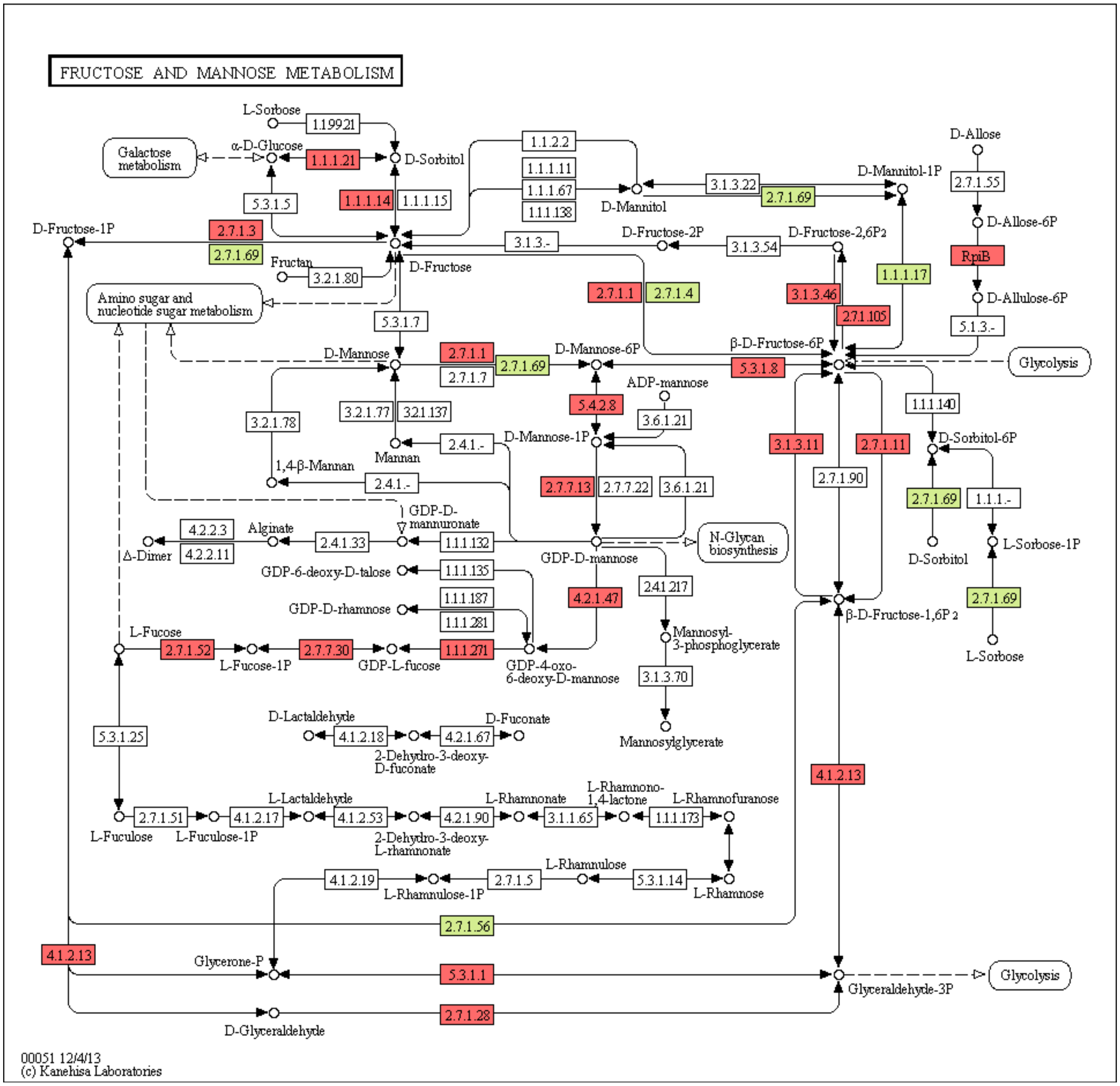
FRUCTOSE AND MANNOSE METABOLISM
L-Sorbose
1.1.99.21
α-D-Glucose
1.1.1.21
D-Sorbitol
Galactose metabolism
5.3.1.5
1.1.1.14
1.1.1.15
1.1.2.2
1.1.1.11
1.1.1.67
1.1.1.138
D-Mannitol
3.1.3.22
2.7.1.69
D-Mannitol-1P
D-Allose
2.7.1.55
D-Allose-6P
RpiB
D-Allulose-6P
5.1.3.-
D-Fructose-1P
2.7.1.3
2.7.1.69
Fructan
3.2.1.80
D-Fructose
3.1.3.-
D-Fructose-2P
3.1.3.54
D-Fructose-2,6P2
1.1.1.17
Amino sugar and nucleotide sugar metabolism
5.3.1.7
2.7.1.1
2.7.1.4
3.1.3.46
2.7.1.105
D-Mannose
2.7.1.1
2.7.1.69
2.7.1.7
D-Mannose-6P
β-D-Fructose-6P
5.3.1.8
Glycolysis
ADP-mannose
5.4.2.8
3.6.1.21
3.2.1.78
3.2.1.77
3.2.1.137
1.1.1.140
D-Sorbitol-6P
3.1.3.11
2.7.1.11
Mannan
2.4.1.-
D-Mannose-1P
1,4-β-Mannan
2.4.1.-
2.7.7.13
2.7.7.22
3.6.1.21
2.7.1.90
2.7.1.69
1.1.1.-
GDP-D-mannuronate
4.2.2.3
4.2.2.11
Alginate
2.4.1.33
1.1.1.132
Δ-Dimer
GDP-D-mannose
N-Glycan biosynthesis
L-Sorbose-1P
D-Sorbitol
GDP-6-deoxy-D-talose
1.1.1.135
GDP-D-rhamnose
1.1.1.187
1.1.1.281
4.2.1.47
2.4.1.217
β-D-Fructose-1,6P2
2.7.1.69
L-Sorbose
L-Fucose
2.7.1.52
L-Fucose-1P
2.7.7.30
GDP-L-fucose
1.1.1.271
GDP-4-oxo-6-deoxy-D-mannose
Mannosyl-3-phosphoglycerate
3.1.3.70
Mannosylglycerate
D-Lactaldehyde
4.1.2.18
2-Dehydro-3-deoxy-D-fuconate
4.2.1.67
D-Fuconate
5.3.1.25
4.1.2.13
L-Fuculose
2.7.1.51
L-Fuculose-1P
4.1.2.17
L-Lactaldehyde
4.1.2.53
2-Dehydro-3-deoxy-L-rhamnonate
4.2.1.90
L-Rhamnonate
3.1.1.65
L-Rhamnono-1,4-lactone
1.1.1.173
L-Rhamnofuranose
4.1.2.19
L-Rhamnulose-1P
2.7.1.5
L-Rhamnulose
5.3.1.14
L-Rhamnose
2.7.1.56
4.1.2.13
Glycerone-P
5.3.1.1
Glyceraldehyde-3P
Glycolysis
D-Glyceraldehyde
2.7.1.28
00051 12/4/13
(c) Kanehisa Laboratories

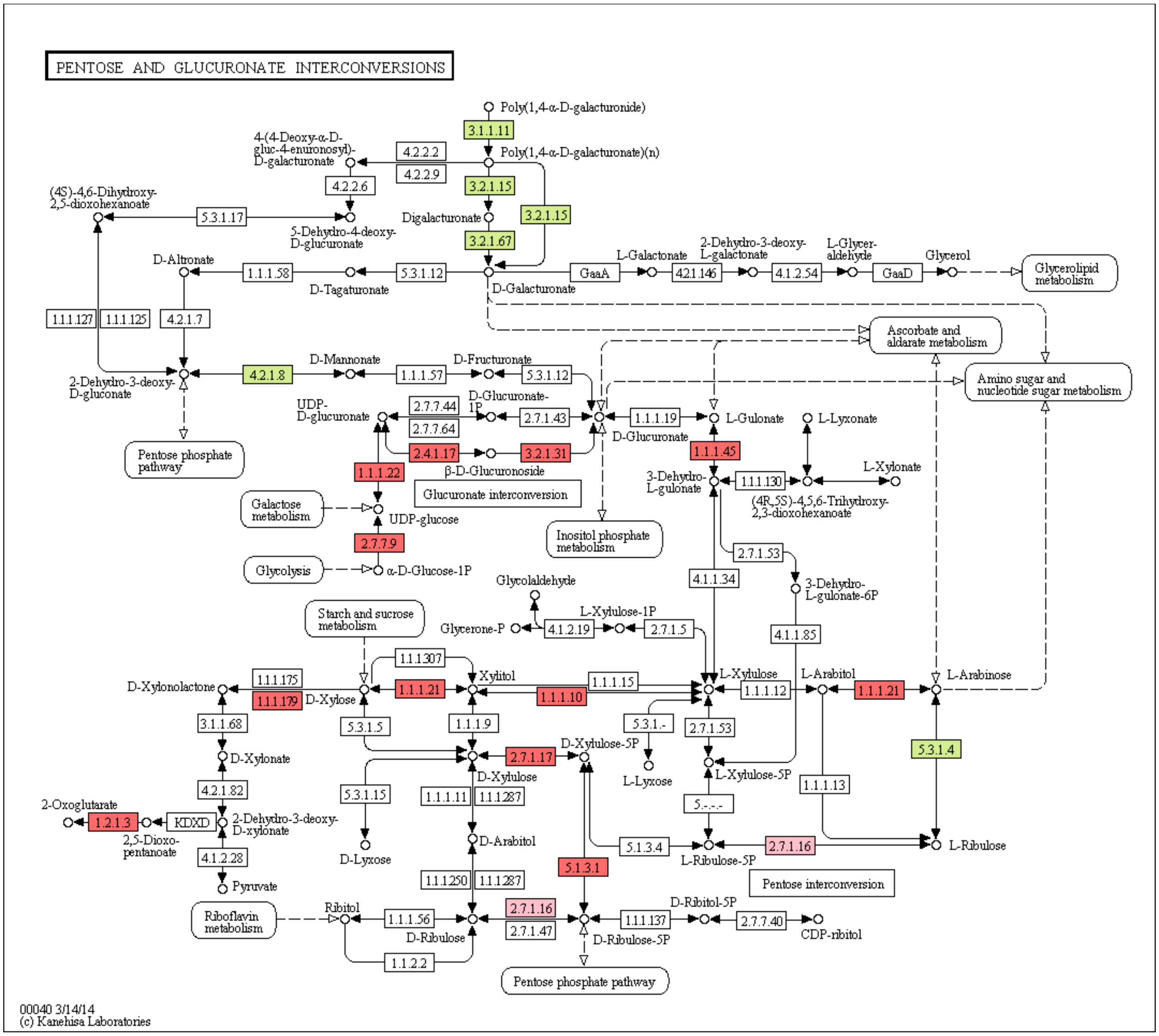
PENTOSE AND GLUCURONATE INTERCONVERSIONS
Poly(1,4-α-D-galacturonide)
3.1.1.11
Poly(1,4-α-D-galacturonate)(n)
4-(4-Deoxy-α-D-gluc-4-enuronosyl)-D-galacturonate
4.2.2.2
4.2.2.9
4.2.2.6
3.2.1.15
3.2.1.15
(4S)-4,6-Dihydroxy-2,5-dioxohexanoate
5.3.1.17
5-Dehydro-4-deoxy-D-glucuronate
Digalacturonate
3.2.1.67
D-Altronate
1.1.1.58
5.3.1.12
D-Tagaturonate
D-Galacturonate
GaaA
L-Galactonate
4.2.1.146
2-Dehydro-3-deoxy-L-galactonate
4.1.2.54
L-Glyceraldehyde
GaaD
Glycerol
Glycerolipid metabolism
1.1.1.127
1.1.1.125
4.2.1.7
Ascorbate and aldarate metabolism
D-Mannonate
D-Fructuronate
4.2.1.8
1.1.1.57
5.3.1.12
Amino sugar and nucleotide sugar metabolism
2-Dehydro-3-deoxy-D-gluconate
UDP-D-glucuronate
2.7.7.44
2.7.7.64
D-Glucuronate-1P
2.7.1.43
1.1.1.19
D-Glucuronate
L-Gulonate
L-Lyxonate
2.4.1.17
3.2.1.31
β-D-Glucuronoside
1.1.1.45
Pentose phosphate pathway
1.1.1.22
Glucuronate interconversion
3-Dehydro-L-gulonate
1.1.1.130
L-Xylonate
(4R,5S)-4,5,6-Trihydroxy-2,3-dioxohexanoate
Galactose metabolism
UDP-glucose
2.7.7.9
Inositol phosphate metabolism
2.7.1.53
Glycolysis
α-D-Glucose-1P
Glycolaldehyde
4.1.1.34
3-Dehydro-L-gulonate-6P
Starch and sucrose metabolism
L-Xylulose-1P
Glycerone-P
4.1.2.19
2.7.1.5
4.1.1.85
1.1.1.307
Xylitol
1.1.1.15
L-Xylulose
L-Arabitol
L-Arabinose
D-Xylonolactone
1.1.1.175
1.1.1.179
D-Xylose
1.1.1.21
1.1.1.10
1.1.1.12
1.1.1.21
3.1.1.68
5.3.1.5
1.1.1.9
5.3.1.-
2.7.1.53
5.3.1.4
D-Xylonate
2.7.1.17
D-Xylulose-5P
D-Xylulose
L-Lyxose
L-Xylulose-5P
4.2.1.82
5.3.1.15
1.1.1.11
1.1.1.287
1.1.1.13
5.-.-.-
2-Oxoglutarate
1.2.1.3
KDXD
2-Dehydro-3-deoxy-D-xylonate
2,5-Dioxopentanoate
D-Lyxose
D-Arabitol
5.1.3.4
2.7.1.16
L-Ribulose-5P
L-Ribulose
4.1.2.28
1.1.1.250
1.1.1.287
5.1.3.1
Pentose interconversion
Pyruvate
Ribitol
2.7.1.16
D-Ribitol-5P
Riboflavin metabolism
1.1.1.56
1.1.1.137
2.7.7.40
CDP-ribitol
D-Ribulose
2.7.1.47
D-Ribulose-5P
1.1.2.2
Pentose phosphate pathway
00040 3/14/14
(c) Kanehisa Laboratories

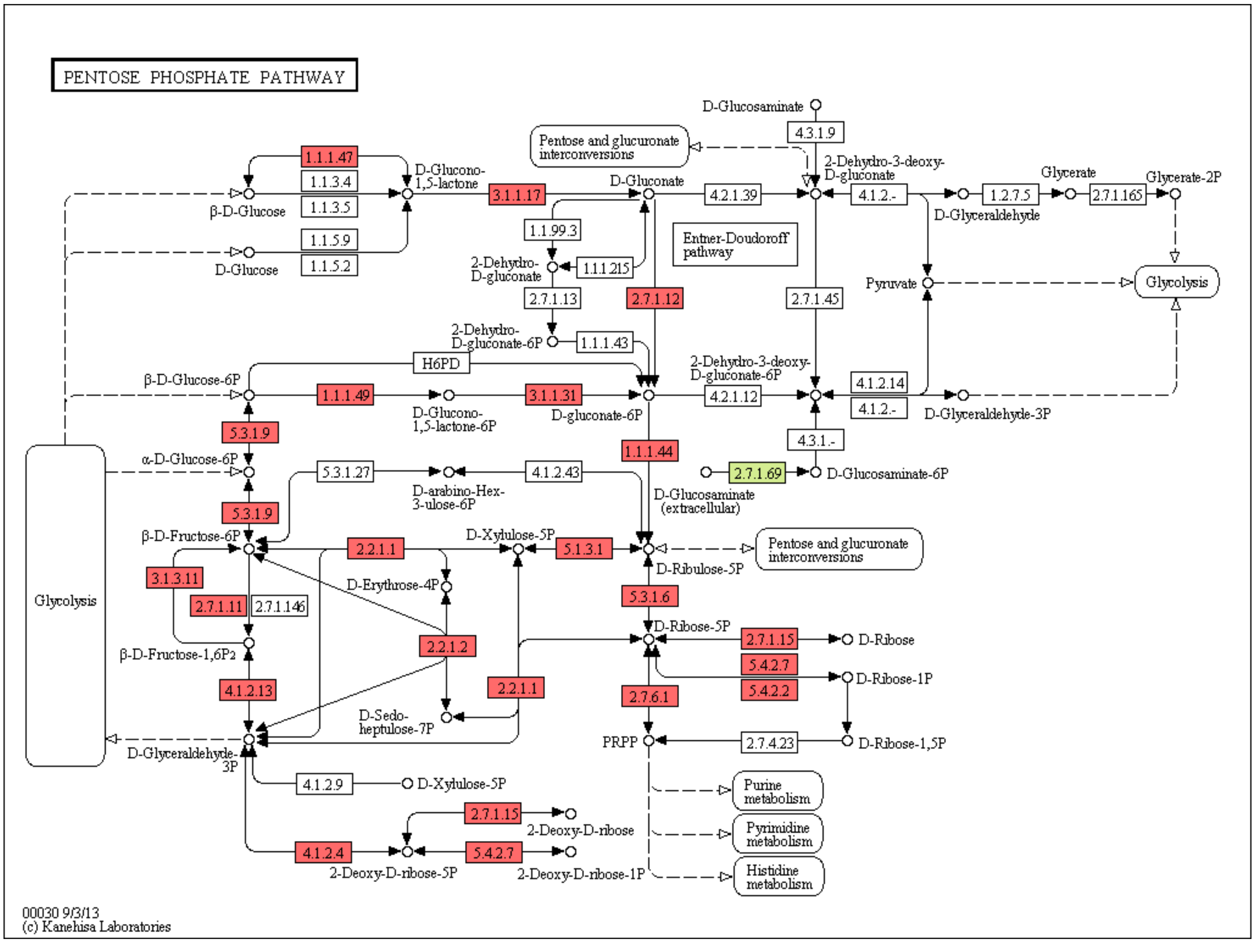
PENTOSE PHOSPHATE PATHWAY
D-Glucosaminate
4.3.1.9
Pentose and glucuronate interconversions
2-Dehydro-3-deoxy-D-gluconate
1.1.1.47
1.1.3.4
1.1.3.5
1.1.5.9
1.1.5.2
D-Glucono-1,5-lactone
β-D-Glucose
D-Glucose
3.1.1.17
D-Gluconate
4.2.1.39
4.1.2.-
D-Glyceraldehyde
1.2.7.5
Glycerate
2.7.1.165
Glycerate-2P
1.1.99.3
1.1.1.215
2-Dehydro-D-gluconate
Entner-Doudoroff pathway
2.7.1.13
2.7.1.12
2.7.1.45
Pyruvate
Glycolysis
2-Dehydro-D-gluconate-6P
1.1.1.43
H6PD
β-D-Glucose-6P
1.1.1.49
D-Glucono-1,5-lactone-6P
3.1.1.31
D-gluconate-6P
4.2.1.12
2-Dehydro-3-deoxy-D-gluconate-6P
4.1.2.14
4.1.2.-
D-Glyceraldehyde-3P
5.3.1.9
1.1.1.44
4.3.1.-
α-D-Glucose-6P
5.3.1.27
D-arabino-Hex-3-ulose-6P
4.1.2.43
2.7.1.69
D-Glucosaminate-6P
D-Glucosaminate (extracellular)
5.3.1.9
β-D-Fructose-6P
2.2.1.1
D-Xylulose-5P
5.1.3.1
D-Ribulose-5P
Pentose and glucuronate interconversions
3.1.3.11
D-Erythrose-4P
2.7.1.11
2.7.1.146
5.3.1.6
D-Ribose-5P
2.7.1.15
D-Ribose
β-D-Fructose-1,6P2
2.2.1.2
5.4.2.7
5.4.2.2
D-Ribose-1P
Glycolysis
4.1.2.13
2.2.1.1
2.7.6.1
D-Sedo-heptulose-7P
PRPP
2.7.4.23
D-Ribose-1,5P
D-Glyceraldehyde-3P
4.1.2.9
D-Xylulose-5P
Purine metabolism
2.7.1.15
2-Deoxy-D-ribose
Pyrimidine metabolism
4.1.2.4
5.4.2.7
2-Deoxy-D-ribose-5P
2-Deoxy-D-ribose-1P
Histidine metabolism
00030 9/3/13
(c) Kanehisa Laboratories

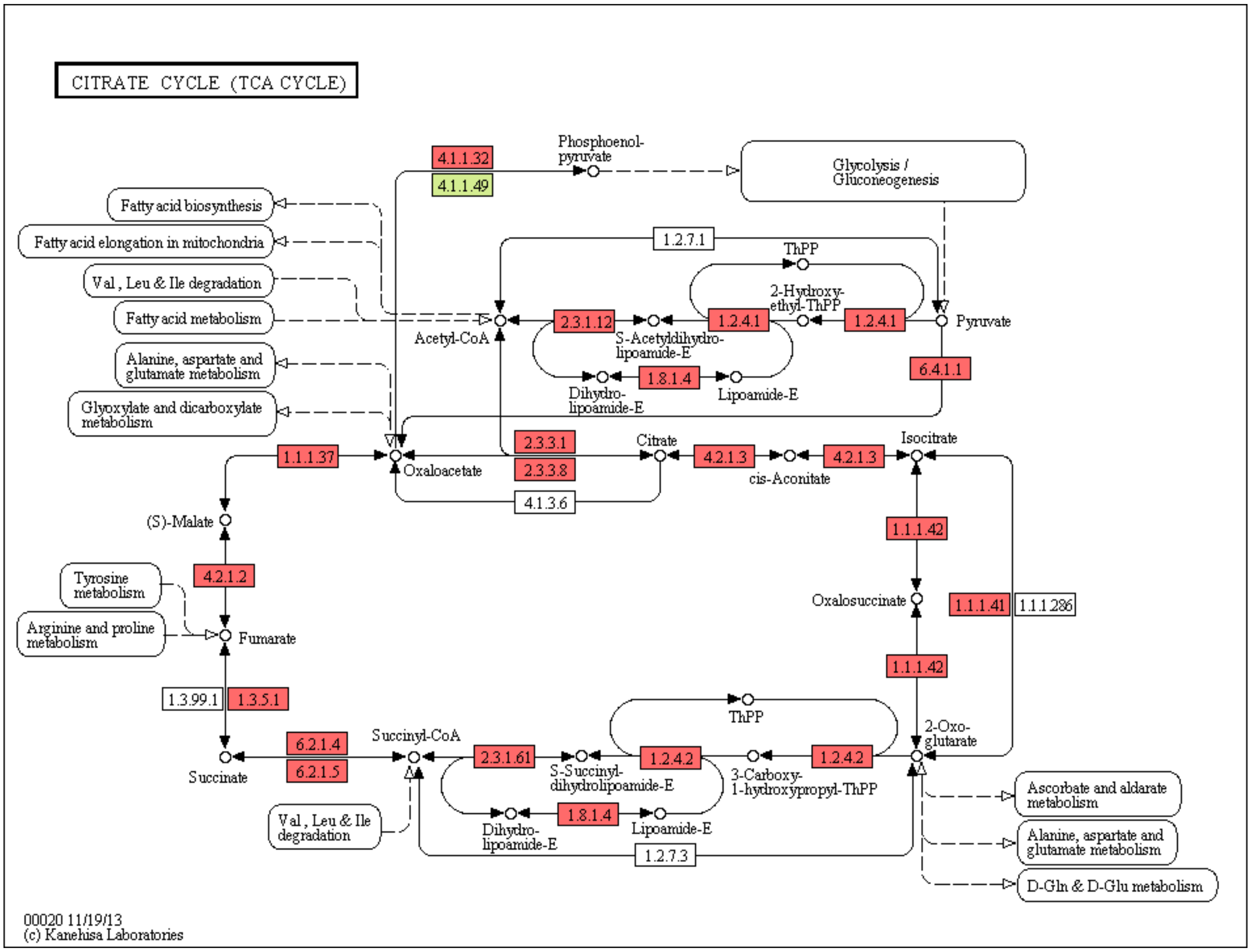

CITRATE CYCLE (TCA CYCLE)
4.1.1.32
4.1.1.49
Phosphoenol-
pyruvate
Glycolysis /
Gluconeogenesis
Fatty acid biosynthesis
Fatty acid elongation in mitochondria
Val, Leu & Ile degradation
Fatty acid metabolism
Alanine, aspartate and
glutamate metabolism
Glyoxylate and dicarboxylate
metabolism
1.2.7.1
ThPP
2-Hydroxy-
ethyl-ThPP
2.3.1.12
1.2.4.1
1.2.4.1
Pyruvate
Acetyl-CoA
S-Acetyldihydro-
lipoamide-E
Dihydro-
lipoamide-E
1.8.1.4
Lipoamide-E
6.4.1.1
1.1.1.37
Oxaloacetate
2.3.3.1
2.3.3.8
Citrate
4.2.1.3
cis-Aconitate
4.2.1.3
Isocitrate
4.1.3.6
(S)-Malate
4.2.1.2
Tyrosine
metabolism
Arginine and proline
metabolism
Fumarate
1.1.1.42
Oxalosuccinate
1.1.1.41
1.1.1.286
1.1.1.42
1.3.99.1
1.3.5.1
ThPP
2-Oxo-
glutarate
6.2.1.4
6.2.1.5
Succinyl-CoA
2.3.1.61
1.2.4.2
1.2.4.2
Succinate
S-Succinyl-
dihydrolipoamide-E
3-Carboxy-
1-hydroxypropyl-ThPP
Val, Leu & Ile
degradation
Dihydro-
lipoamide-E
1.8.1.4
Lipoamide-E
1.2.7.3
Ascorbate and aldarate
metabolism
Alanine, aspartate and
glutamate metabolism
D-Gln & D-Glu metabolism
00020 11/19/13
(c) Kanehisa Laboratories

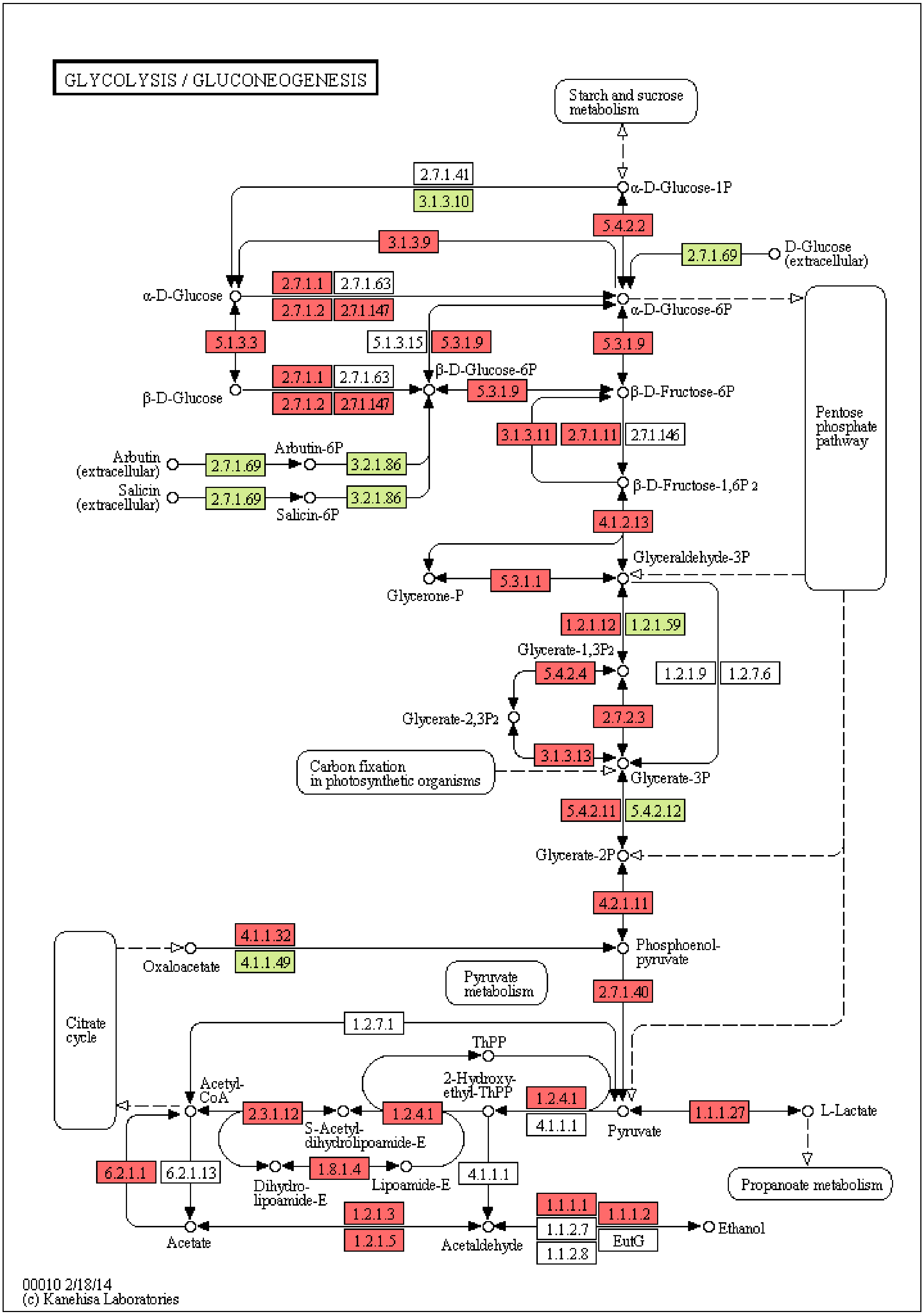

GLYCOLYSIS / GLUCONEOGENESIS
Starch and sucrose metabolism
2.7.1.41
3.1.3.10
α-D-Glucose-1P
5.4.2.2
3.1.3.9
2.7.1.69
D-Glucose (extracellular)
α-D-Glucose
2.7.1.1
2.7.1.63
2.7.1.2
2.7.1.147
α-D-Glucose-6P
5.1.3.3
5.1.3.15
5.3.1.9
β-D-Glucose
β-D-Glucose-6P
β-D-Fructose-6P
3.1.3.11
2.7.1.11
2.7.1.146
Pentose phosphate pathway
Arbutin (extracellular)
Arbutin-6P
3.2.1.86
Salicin (extracellular)
Salicin-6P
β-D-Fructose-1,6P2
4.1.2.13
Glyceraldehyde-3P
Glycerone-P
5.3.1.1
1.2.1.12
1.2.1.59
Glycerate-1,3P2
5.4.2.4
1.2.1.9
1.2.7.6
Glycerate-2,3P2
2.7.2.3
3.1.3.13
Carbon fixation in photosynthetic organisms
Glycerate-3P
5.4.2.11
5.4.2.12
Glycerate-2P
4.2.1.11
4.1.1.32
4.1.1.49
Citrate cycle
Oxaloacetate
Phosphoenol-pyruvate
Pyruvate metabolism
2.7.1.40
1.2.7.1
ThPP
2-Hydroxy-ethyl-ThPP
Acetyl-CoA
2.3.1.12
1.2.4.1
4.1.1.1
Pyruvate
1.1.1.27
L-Lactate
S-Acetyl-dihydrolipoamide-E
6.2.1.1
6.2.1.13
1.8.1.4
Dihydro-lipoamide-E
Lipoamide-E
Propanoate metabolism
1.2.1.3
1.2.1.5
1.1.1.1
1.1.1.2
1.1.2.7
1.1.2.8
EutG
Acetate
Acetaldehyde
Ethanol
00010 2/18/14
(c) Kanehisa Laboratories
