# Supplementary material for: Application of a hierarchical enzyme classification method reveals the role of gut microbiome in human metabolism
Source: BMC Genomics. 2015 Jun 11;16(Suppl 7):S16. doi: 10.1186/1471-2164-16-S7-S16 (PMC4474468; doi:10.1186/1471-2164-16-S7-S16)
Supplement: Additional file 9 — Figure S4. GMC pathways that reveal the newly discovered role of gut bacteria in human metabolism [file 1471-2164-16-S7-S16-S9.pdf]

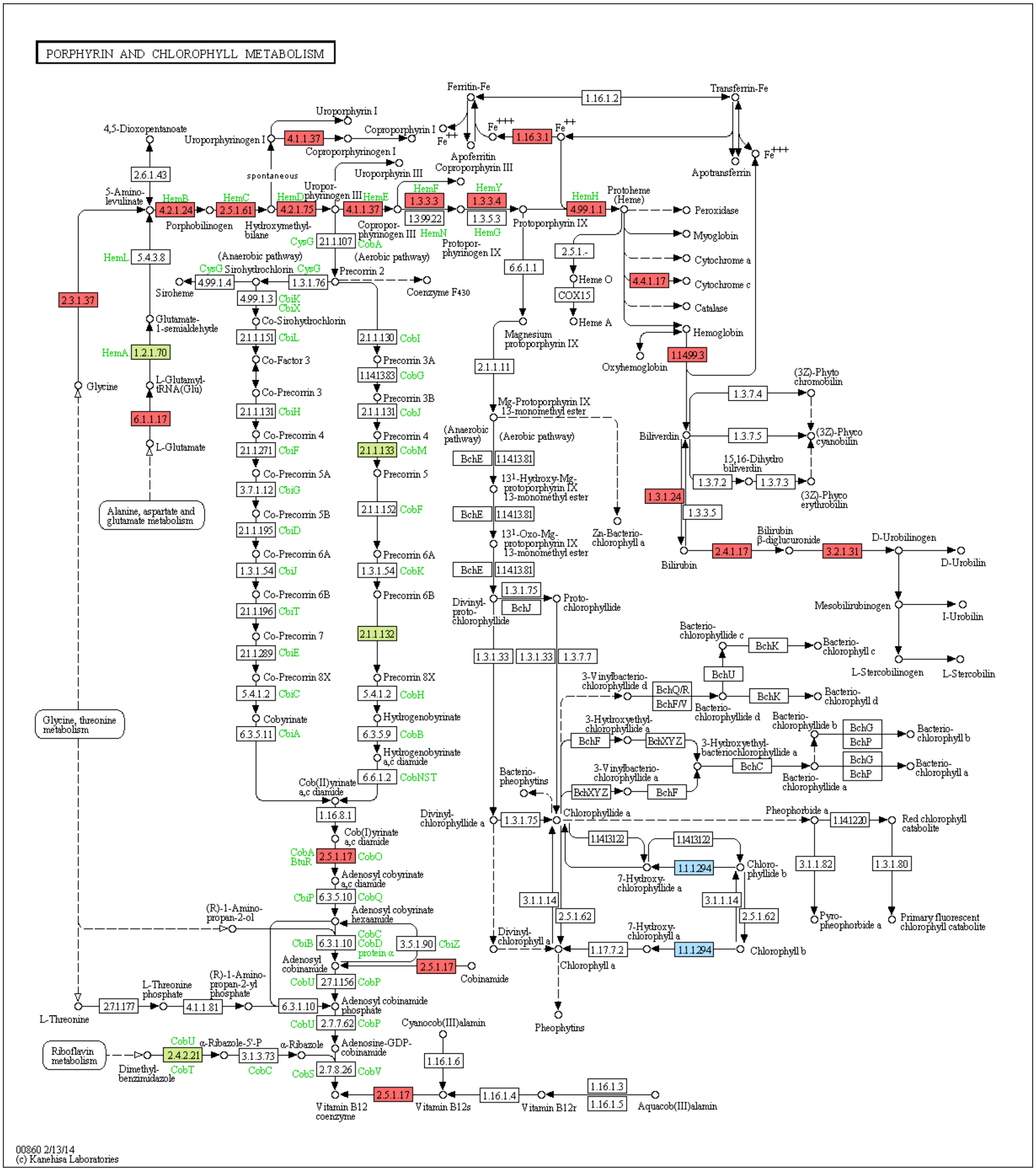
PORPHYRIN AND CHLOROPHYLL METABOLISM
Ferritin-Fe
1.16.1.2
Transferrin-Fe
Uroporphyrin I
4,5-Dioxopentanoate
Uroporphyrinogen I
4.1.1.37
Coproporphyrin I
Coproporphyrinogen I
1.16.3.1
Apoferritin
Coproporphyrin III
Apotransferrin
2.6.1.43
spontaneous
Uroporphyrin III
5-Amino-levulinate
HemB
4.2.1.24
HemC
2.5.1.61
HemD
4.2.1.75
Uropor-phyrinogen III
HemE
4.1.1.37
HemF
1.3.3.3
HemY
1.3.3.4
HemH
4.99.1.1
Protoheme (Heme)
Peroxidase
1.3.99.22
1.3.5.3
Porphobilinogen
Hydroxymethyl-bilane
Copropor-phyrinogen III
Protoporphyrin IX
Myoglobin
CysG
2.1.1.107
CobA
HemN
HemG
Protopor-phyrinogen IX
HemL
5.4.3.8
(Anaerobic pathway)
(Aerobic pathway)
2.5.1.-
Cytochrome a
Sirohydrochlorin
Precorrin 2
6.6.1.1
Siroheme
4.99.1.4
1.3.1.76
Coenzyme F430
Heme O
4.4.1.17
Cytochrome c
2.3.1.37
4.99.1.3
CbiK
CbiX
COX15
Catalase
Glutamate-1-semialdehyde
Co-Sirohydrochlorin
Heme A
Hemoglobin
HemA
1.2.1.70
2.1.1.151
CbiL
2.1.1.130
CobI
Magnesium protoporphyrin IX
Co-Factor 3
Precorrin 3A
Oxyhemoglobin
1.14.99.3
Glycine
L-Glutamyl-tRNA(Glu)
1.14.13.83
CobG
2.1.1.11
(3Z)-Phyto chromobilin
Co-Precorrin 3
Precorrin 3B
1.3.7.4
6.1.1.17
2.1.1.131
CbiH
2.1.1.131
CobJ
Mg-Protoporphyrin IX 13-monomethyl ester
Co-Precorrin 4
Precorrin 4
(Anaerobic pathway)
(Aerobic pathway)
Biliverdin
1.3.7.5
(3Z)-Phyco cyanobilin
L-Glutamate
2.1.1.271
CbiF
2.1.1.133
CobM
BchE
1.14.13.81
15,16-Dihydro biliverdin
Co-Precorrin 5A
Precorrin 5
1.3.7.2
1.3.7.3
3.7.1.12
CbiG
13¹-Hydroxy-Mg-protoporphyrin IX 13-monomethyl ester
(3Z)-Phyco erythrobilin
1.3.1.24
Alanine, aspartate and glutamate metabolism
Co-Precorrin 5B
2.1.1.152
CobF
BchE
1.14.13.81
1.3.3.5
2.1.1.195
CbiD
13¹-Oxo-Mg-protoporphyrin IX 13-monomethyl ester
Zn-Bacterio-chlorophyll a
Bilirubin β-diglucuronide
D-Urobilinogen
Co-Precorrin 6A
Precorrin 6A
2.4.1.17
3.2.1.31
1.3.1.54
CbiJ
1.3.1.54
CobK
BchE
1.14.13.81
Bilirubin
D-Urobilin
Co-Precorrin 6B
Precorrin 6B
1.3.1.75
Divinyl-proto-chlorophyllide
Proto-chlorophyllide
2.1.1.196
CbiT
BchJ
Mesobilirubinogen
I-Urobilin
Co-Precorrin 7
2.1.1.132
Bacterio-chlorophyllide c
BchK
Bacterio-chlorophyll c
2.1.1.289
CbiE
1.3.1.33
1.3.1.33
1.3.7.7
L-Stercobilinogen
L-Stercobilin
Co-Precorrin 8X
Precorrin 8X
BchU
3-Vinylbacterio-chlorophyllide d
BchQ/R
BchF/V
BchK
Bacterio-chlorophyll d
5.4.1.2
CbiC
5.4.1.2
CobH
Bacterio-chlorophyllide d
Glycine, threonine metabolism
Cobyrinate
Hydrogenobyrinate
3-Hydroxyethyl-chlorophyllide a
Bacterio-chlorophyllide b
BchG
BchP
Bacterio-chlorophyll b
6.3.5.11
CbiA
6.3.5.9
CobB
BchF
BchXYZ
3-Hydroxyethyl-bacteriochlorophyllide a
Hydrogenobyrinate a,c diamide
BchG
BchP
3-Vinylbacterio-chlorophyllide a
BchC
Bacterio-chlorophyll a
Cob(II)yrinate a,c diamide
6.6.1.2
CobNST
Bacterio-pheophytins
Bacterio-chlorophyllide a
BchXYZ
BchF
1.16.8.1
Divinyl-chlorophyllide a
1.3.1.75
Chlorophyllide a
Pheophorbide a
1.14.12.20
Red chlorophyll catabolite
Cob(I)yrinate a,c diamide
1.14.13.122
1.14.13.122
CobA
BtuR
2.5.1.17
CobO
1.1.1.294
Chloro-phyllide b
Adenosyl cobyrinate a,c diamide
7-Hydroxy-chlorophyllide a
3.1.1.82
1.3.1.80
CbiP
6.3.5.10
CobQ
3.1.1.14
3.1.1.14
(R)-1-Amino-propan-2-ol
Adenosyl cobyrinate hexaamide
2.5.1.62
2.5.1.62
Pyro-pheophorbide a
Primary fluorescent chlorophyll catabolite
CbiB
6.3.1.10
CobC
CobD
protein α
3.5.1.90
CbiZ
Divinyl-chlorophyll a
7-Hydroxy-chlorophyll a
1.17.7.2
1.1.1.294
Chlorophyll b
Adenosyl cobinamide
2.5.1.17
Cobinamide
Chlorophyll a
L-Threonine phosphate
(R)-1-Amino-propan-2-yl phosphate
CobU
2.7.1.156
CobP
2.7.1.177
4.1.1.81
6.3.1.10
Adenosyl cobinamide phosphate
L-Threonine
CobU
2.7.7.62
CobP
Cyanocob(III)alamin
Pheophytins
Riboflavin metabolism
CobU
2.4.2.21
α-Ribazole-5'-P
3.1.3.73
α-Ribazole
Adenosine-GDP-cobinamide
1.16.1.6
Dimethyl-benzimidazole
CobT
CobC
CobS
2.7.8.26
CobV
1.16.1.3
2.5.1.17
1.16.1.4
1.16.1.5
Vitamin B12 coenzyme
Vitamin B12s
Vitamin B12r
Aquacob(III)alamin
00860 2/13/14
(c) Kanehisa Laboratories

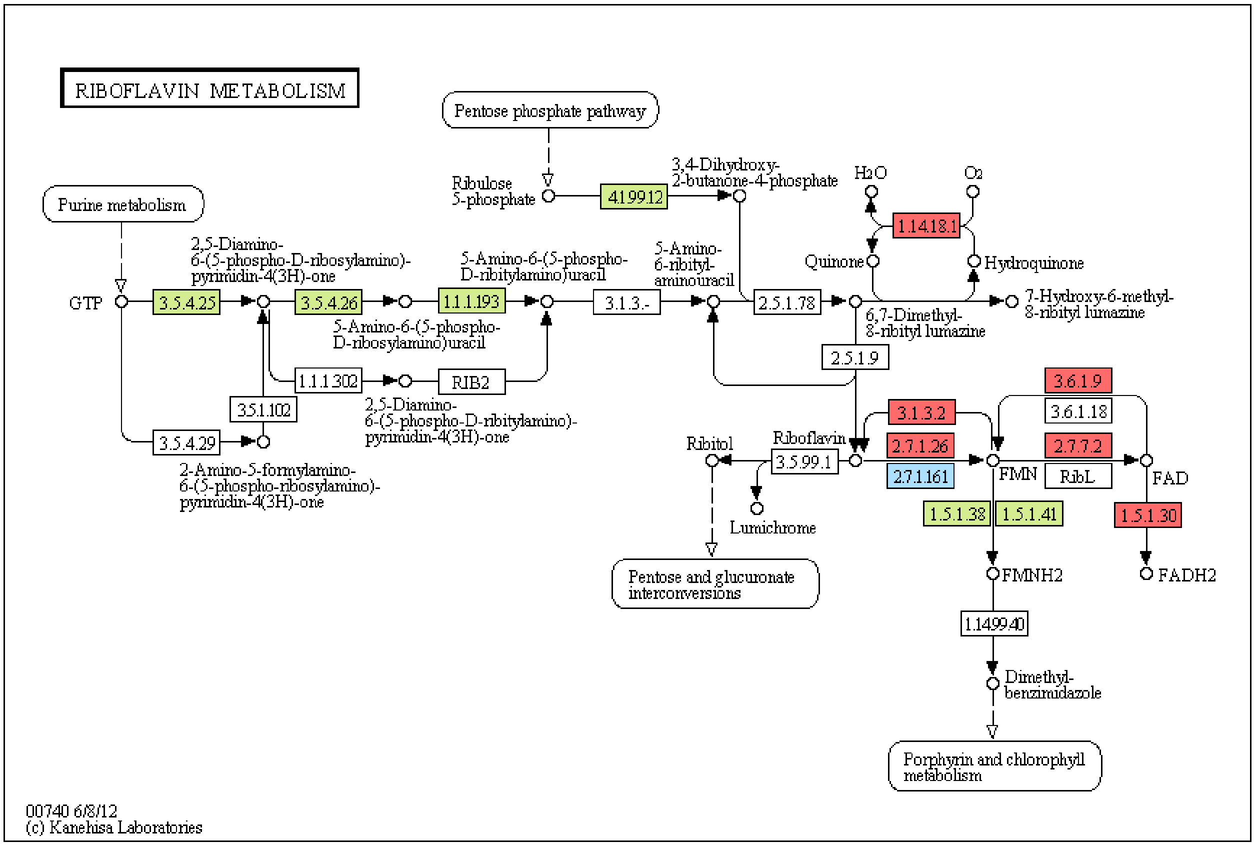
RIBOFLAVIN METABOLISM
Pentose phosphate pathway
Purine metabolism
Ribulose 5-phosphate
4.1.99.12
3,4-Dihydroxy-2-butanone-4-phosphate
H2O
O2
1.14.18.1
Quinone
Hydroquinone
2,5-Diamino-6-(5-phospho-D-ribosylamino)-pyrimidin-4(3H)-one
5-Amino-6-(5-phospho-D-ribitylamino)uracil
5-Amino-6-ribityl-aminouracil
GTP
3.5.4.25
3.5.4.26
1.1.1.193
3.1.3.-
2.5.1.78
7-Hydroxy-6-methyl-8-ribityl lumazine
6,7-Dimethyl-8-ribityl lumazine
5-Amino-6-(5-phospho-D-ribosylamino)uracil
2.5.1.9
1.1.1.302
RIB2
3.5.1.102
2,5-Diamino-6-(5-phospho-D-ribitylamino)-pyrimidin-4(3H)-one
3.5.4.29
2-Amino-5-formylamino-6-(5-phospho-ribosylamino)-pyrimidin-4(3H)-one
3.6.1.9
3.6.1.18
3.1.3.2
Ribitol
Riboflavin
2.7.1.26
2.7.7.2
3.5.99.1
FMN
2.7.1.161
RibL
FAD
1.5.1.38
1.5.1.41
1.5.1.30
Lumichrome
Pentose and glucuronate interconversions
FMNH2
FADH2
1.14.99.40
Dimethyl-benzimidazole
Porphyrin and chlorophyll metabolism
00740 6/8/12
(c) Kanehisa Laboratories

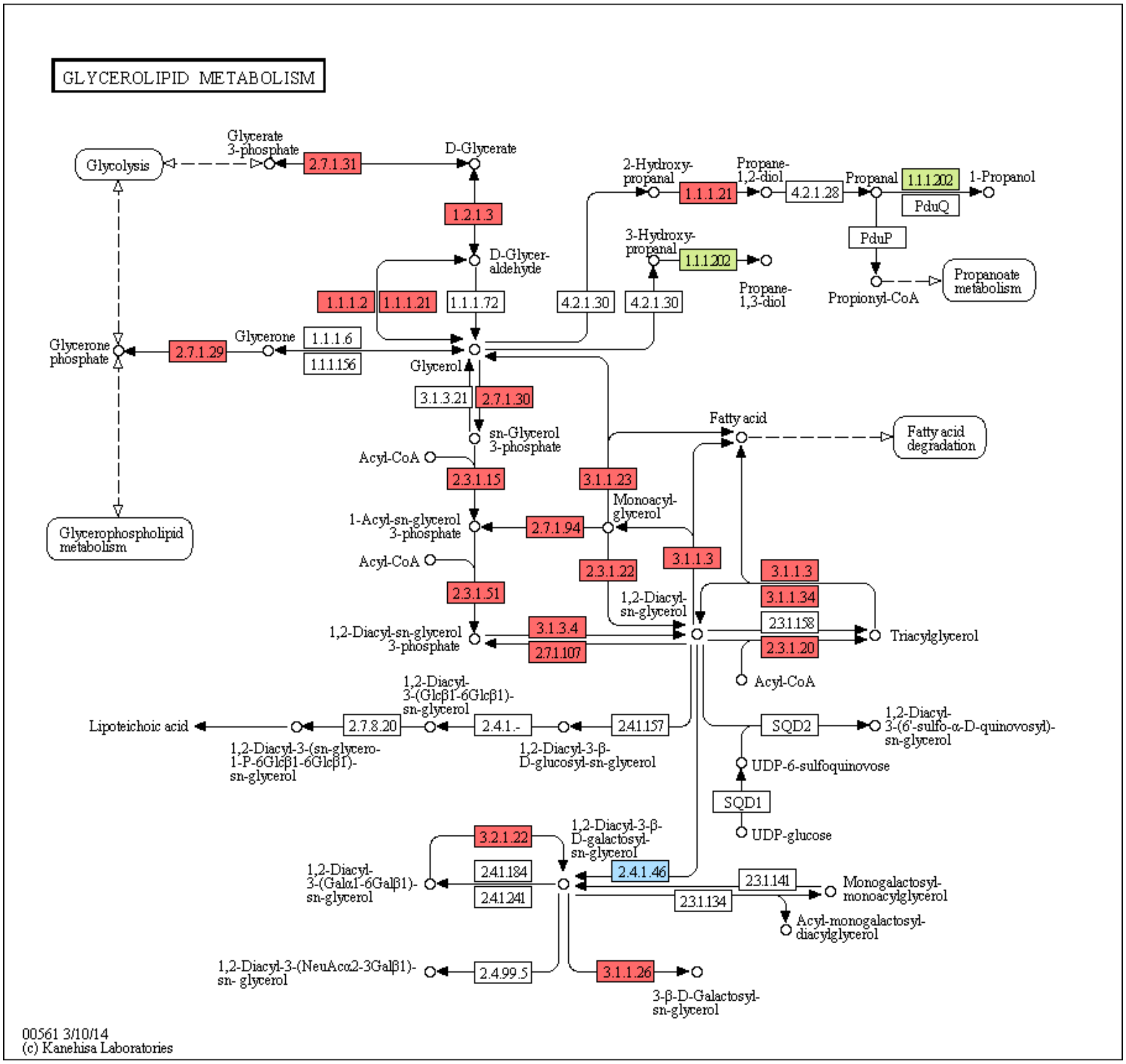
GLYCEROLIPID METABOLISM
Glycolysis
Glycerate 3-phosphate
2.7.1.31
D-Glycerate
1.2.1.3
D-Glyceraldehyde
2-Hydroxy-propanal
1.1.1.21
Propane-1,2-diol
4.2.1.28
Propanal
1.1.1.202
1-Propanol
PduQ
PduP
Propionyl-CoA
Propanoate metabolism
3-Hydroxy-propanal
1.1.1.202
Propane-1,3-diol
1.1.1.2
1.1.1.21
1.1.1.72
4.2.1.30
4.2.1.30
Glycerone phosphate
2.7.1.29
Glycerone
1.1.1.6
1.1.1.156
Glycerol
3.1.3.21
2.7.1.30
sn-Glycerol 3-phosphate
Acyl-CoA
2.3.1.15
Fatty acid
Fatty acid degradation
2.3.1.23
Monoacyl-glycerol
1-Acyl-sn-glycerol 3-phosphate
2.7.1.94
Glycerophospholipid metabolism
3.1.1.3
Acyl-CoA
2.3.1.22
3.1.1.3
3.1.1.34
2.3.1.51
1,2-Diacyl-sn-glycerol
1,2-Diacyl-sn-glycerol 3-phosphate
3.1.3.4
2.7.1.107
2.3.1.158
2.3.1.20
Triacylglycerol
Acyl-CoA
1,2-Diacyl-3-(Glcβ1-6Glcβ1)-sn-glycerol
Lipoteichoic acid
2.7.8.20
2.4.1.-
2.4.1.157
SQD2
1,2-Diacyl-3-(6'-sulfo-α-D-quinovosyl)-sn-glycerol
1,2-Diacyl-3-(sn-glycero-1-P-6Glcβ1-6Glcβ1)-sn-glycerol
1,2-Diacyl-3-β-D-glucosyl-sn-glycerol
UDP-6-sulfoquinovose
SQD1
UDP-glucose
3.2.1.22
1,2-Diacyl-3-β-D-galactosyl-sn-glycerol
2.4.1.46
1,2-Diacyl-3-(Galα1-6Galβ1)-sn-glycerol
2.4.1.184
2.4.1.241
2.3.1.141
Monogalactosyl-monoacylglycerol
2.3.1.134
Acyl-monogalactosyl-diacylglycerol
1,2-Diacyl-3-(NeuAcα2-3Galβ1)-sn- glycerol
2.4.99.5
3.1.1.26
3-β-D-Galactosyl-sn-glycerol
00561 3/10/14
(c) Kanehisa Laboratories

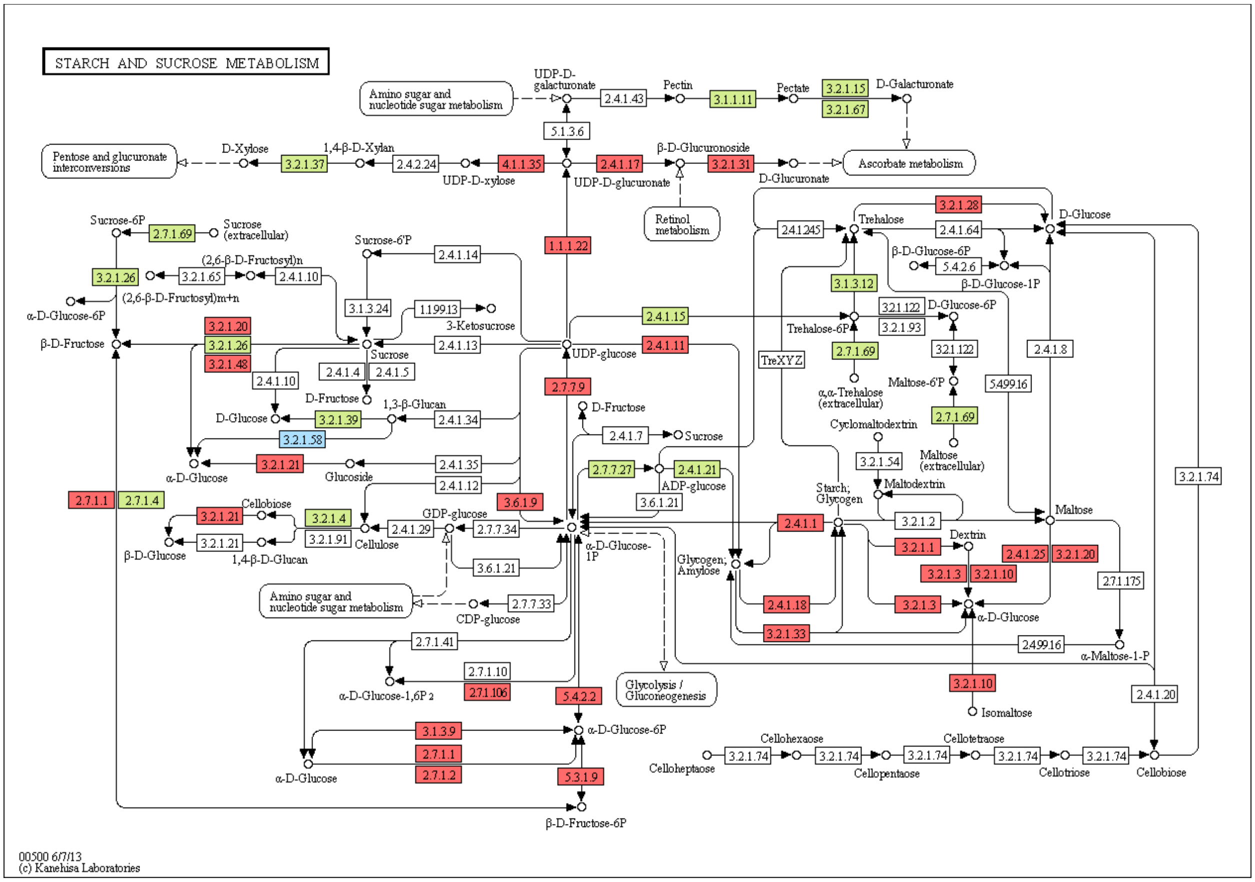
STARCH AND SUCROSE METABOLISM
Amino sugar and nucleotide sugar metabolism
UDP-D-galacturonate
2.4.1.43
Pectin
3.1.1.11
Pectate
3.2.1.15
3.2.1.67
D-Galacturonate
5.1.3.6
Pentose and glucuronate interconversions
D-Xylose
3.2.1.37
1,4-β-D-Xylan
2.4.2.24
4.1.1.35
UDP-D-xylose
2.4.1.17
UDP-D-glucuronate
β-D-Glucuronoside
3.2.1.31
D-Glucuronate
Ascorbate metabolism
Retinol metabolism
Sucrose-6P
2.7.1.69
Sucrose (extracellular)
3.2.1.26
(2,6-β-D-Fructosyl)n
3.2.1.65
2.4.1.10
(2,6-β-D-Fructosyl)m+n
α-D-Glucose-6P
Sucrose-6'P
2.4.1.14
1.1.1.22
3.1.3.24
1.1.99.13
3-Ketosucrose
β-D-Fructose
3.2.1.20
3.2.1.26
3.2.1.48
Sucrose
2.4.1.13
UDP-glucose
2.4.1.11
2.4.1.4
2.4.1.5
2.4.1.10
D-Fructose
1,3-β-Glucan
D-Glucose
3.2.1.39
2.4.1.34
3.2.1.58
2.7.7.9
D-Fructose
2.4.1.7
Sucrose
α-D-Glucose
3.2.1.21
Glucoside
2.4.1.35
2.4.1.12
2.7.7.27
ADP-glucose
2.4.1.21
3.6.1.21
2.7.1.1
2.7.1.4
Cellobiose
3.2.1.21
3.2.1.4
GDP-glucose
3.6.1.9
β-D-Glucose
3.2.1.21
1,4-β-D-Glucan
3.2.1.91
Cellulose
2.4.1.29
2.7.7.34
α-D-Glucose-1P
3.6.1.21
Amino sugar and nucleotide sugar metabolism
CDP-glucose
2.7.7.33
2.7.1.41
α-D-Glucose-1,6P2
2.7.1.10
2.7.1.106
5.4.2.2
Glycolysis / Gluconeogenesis
3.1.3.9
α-D-Glucose-6P
2.7.1.1
α-D-Glucose
2.7.1.2
5.3.1.9
β-D-Fructose-6P
3.2.1.28
D-Glucose
2.4.1.245
Trehalose
2.4.1.64
β-D-Glucose-6P
5.4.2.6
β-D-Glucose-1P
3.1.3.12
2.4.1.15
3.2.1.122
D-Glucose-6P
Trehalose-6P
3.2.1.93
TreXYZ
2.7.1.69
3.2.1.122
2.4.1.8
α,α-Trehalose (extracellular)
Maltose-6'P
5.4.99.16
2.7.1.69
Cyclomaltodextrin
Maltose (extracellular)
3.2.1.54
Maltodextrin
Starch; Glycogen
3.2.1.2
Maltose
3.2.1.74
2.4.1.1
Dextrin
Glycogen; Amylose
3.2.1.1
2.4.1.25
3.2.1.20
3.2.1.3
3.2.1.10
2.7.1.175
2.4.1.18
3.2.1.3
α-D-Glucose
3.2.1.33
2.4.99.16
α-Maltose-1-P
3.2.1.10
2.4.1.20
Isomaltose
Cellohexaose
Cellotetraose
Celloheptaose
3.2.1.74
3.2.1.74
3.2.1.74
3.2.1.74
3.2.1.74
Cellopentaose
Cellotriose
Cellobiose
00500 6/7/13
(c) Kanehisa Laboratories

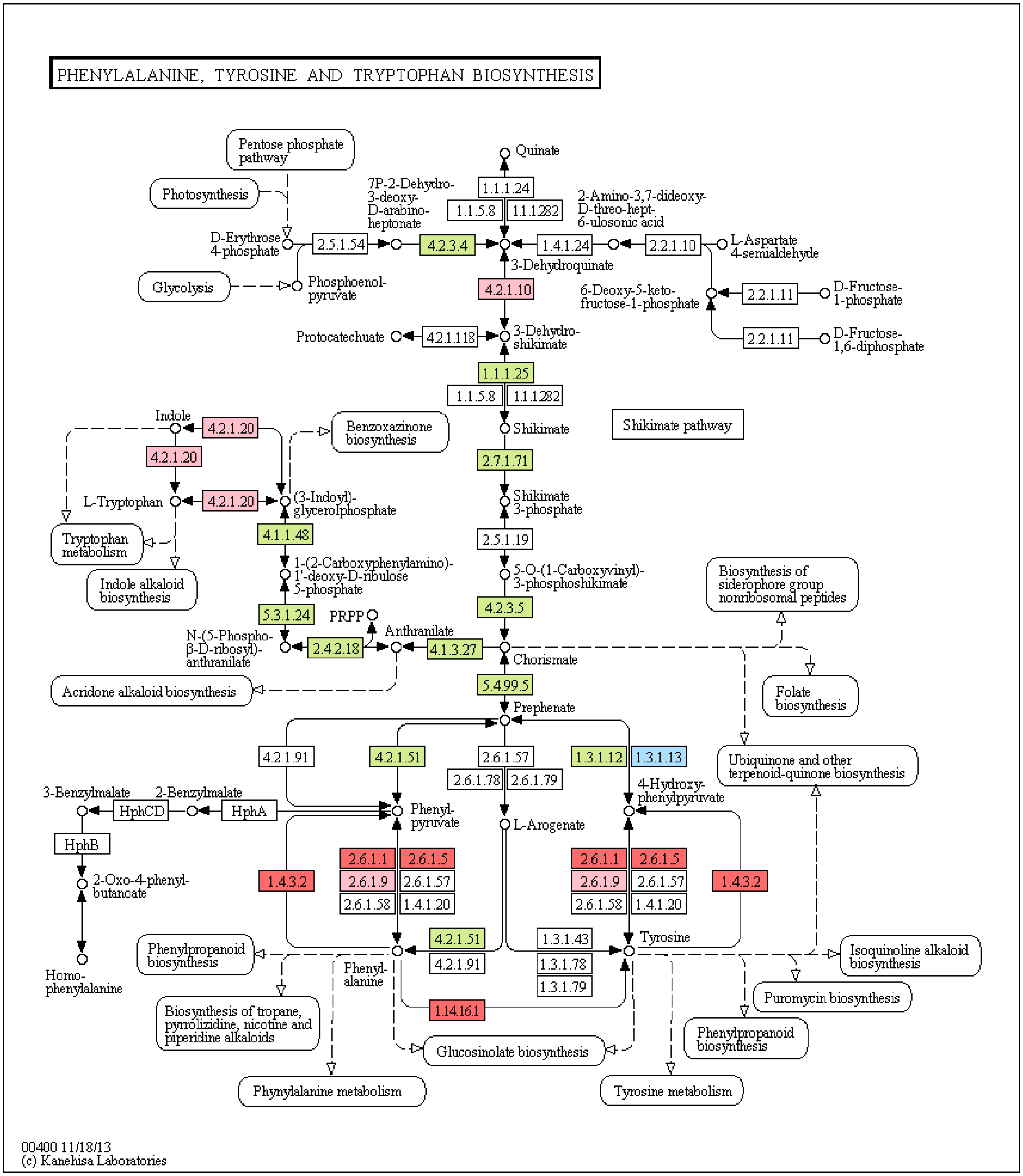
PHENYLALANINE, TYROSINE AND TRYPTOPHAN BIOSYNTHESIS
Pentose phosphate pathway
Photosynthesis
Glycolysis
D-Erythrose 4-phosphate
Phosphoenol-pyruvate
2.5.1.54
7P-2-Dehydro-3-deoxy-D-arabino-heptonate
4.2.3.4
Quinate
1.1.1.24
1.1.5.8
1.1.1.282
2-Amino-3,7-dideoxy-D-threo-hept-6-ulosonic acid
1.4.1.24
2.2.1.10
L-Aspartate 4-semialdehyde
3-Dehydroquinate
4.2.1.10
6-Deoxy-5-keto-fructose-1-phosphate
2.2.1.11
D-Fructose-1-phosphate
2.2.1.11
D-Fructose-1,6-diphosphate
Protocatechuate
4.2.1.118
3-Dehydro-shikimate
1.1.1.25
1.1.5.8
1.1.1.282
Shikimate
Shikimate pathway
2.7.1.71
Shikimate 3-phosphate
2.5.1.19
5-O-(1-Carboxyvinyl)-3-phosphoshikimate
4.2.3.5
Chorismate
Biosynthesis of siderophore group nonribosomal peptides
Folate biosynthesis
Indole
4.2.1.20
4.2.1.20
Benzoxazinone biosynthesis
L-Tryptophan
4.2.1.20
(3-Indoyl)-glycerolphosphate
4.1.1.48
Tryptophan metabolism
Indole alkaloid biosynthesis
1-(2-Carboxyphenylamino)-1'-deoxy-D-ribulose 5-phosphate
5.3.1.24
PRPP
N-(5-Phospho-β-D-ribosyl)-anthranilate
2.4.2.18
Anthranilate
4.1.3.27
Acridone alkaloid biosynthesis
5.4.99.5
Prephenate
4.2.1.91
4.2.1.51
2.6.1.57
2.6.1.78
2.6.1.79
1.3.1.12
1.3.1.13
Ubiquinone and other terpenoid-quinone biosynthesis
3-Benzylmalate
2-Benzylmalate
HphCD
HphA
HphB
2-Oxo-4-phenyl-butanoate
Homo-phenylalanine
Phenyl-pyruvate
L-Arogenate
4-Hydroxy-phenylpyruvate
1.4.3.2
2.6.1.1
2.6.1.5
2.6.1.9
2.6.1.57
2.6.1.58
1.4.1.20
2.6.1.1
2.6.1.5
2.6.1.9
2.6.1.57
2.6.1.58
1.4.1.20
1.4.3.2
Phenylpropanoid biosynthesis
4.2.1.51
4.2.1.91
Phenyl-alanine
1.3.1.43
1.3.1.78
1.3.1.79
Tyrosine
Isoquinoline alkaloid biosynthesis
Puromycin biosynthesis
Phenylpropanoid biosynthesis
1.14.16.1
Biosynthesis of tropane, pyrrolizidine, nicotine and piperidine alkaloids
Glucosinolate biosynthesis
Phynylalanine metabolism
Tyrosine metabolism
00400 11/18/13
(c) Kanehisa Laboratories

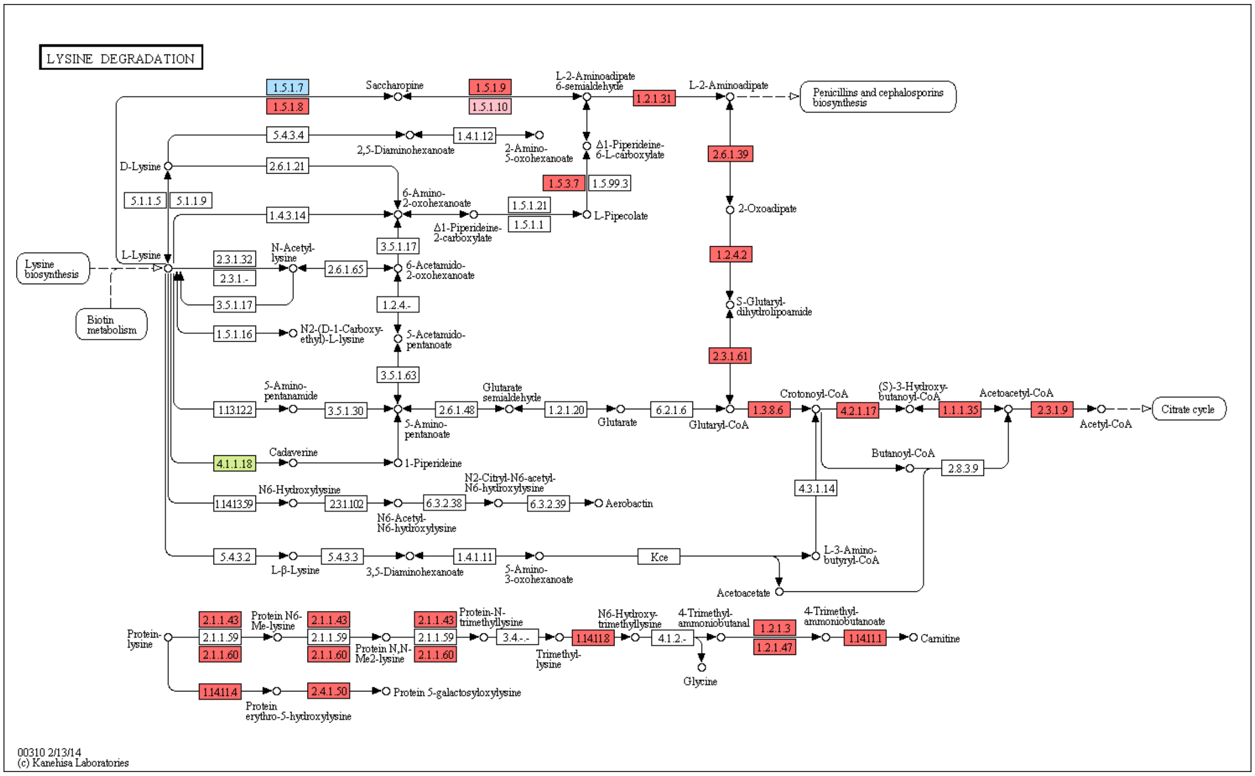

LYSINE DEGRADATION
1.5.1.7
1.5.1.8
Saccharopine
1.5.1.9
1.5.1.10
L-2-Aminoadipate 6-semialdehyde
1.2.1.31
L-2-Aminoadipate
Penicillins and cephalosporins biosynthesis
5.4.3.4
1.4.1.12
2,5-Diaminohexanoate
2-Amino-5-oxohexanoate
Δ1-Piperideine-6-L-carboxylate
2.6.1.39
D-Lysine
2.6.1.21
1.5.3.7
1.5.99.3
5.1.1.5
5.1.1.9
6-Amino-2-oxohexanoate
1.5.1.21
1.5.1.1
L-Pipecolate
2-Oxoadipate
1.4.3.14
Δ1-Piperideine-2-carboxylate
3.5.1.17
L-Lysine
N-Acetyl-lysine
2.3.1.32
2.3.1.-
2.6.1.65
6-Acetamido-2-oxohexanoate
1.2.4.2
Lysine biosynthesis
3.5.1.17
1.2.4.-
S-Glutaryl-dihydrolipoamide
Biotin metabolism
1.5.1.16
N2-(D-1-Carboxyethyl)-L-lysine
5-Acetamido-pentanoate
2.3.1.61
3.5.1.63
5-Amino-pentanamide
Glutarate semialdehyde
Crotonoyl-CoA
(S)-3-Hydroxy-butanoyl-CoA
Acetoacetyl-CoA
1.13.12
3.5.1.30
2.6.1.48
1.2.1.20
6.2.1.6
1.3.8.6
4.2.1.17
1.1.1.35
2.3.1.9
Citrate cycle
5-Amino-pentanoate
Glutarate
Glutaryl-CoA
Acetyl-CoA
Cadaverine
Butanoyl-CoA
4.1.1.18
1-Piperideine
2.8.3.9
N6-Hydroxylysine
N2-Citryl-N6-acetyl-N6-hydroxylysine
4.3.1.14
1.14.13.59
2.3.1.102
6.3.2.38
6.3.2.39
Aerobactin
N6-Acetyl-N6-hydroxylysine
5.4.3.2
5.4.3.3
1.4.1.11
Kce
L-3-Amino-butyryl-CoA
L-β-Lysine
3,5-Diaminohexanoate
5-Amino-3-oxohexanoate
Acetoacetate
Protein-lysine
2.1.1.43
2.1.1.59
2.1.1.60
Protein N6-Me-lysine
Protein N,N-Me2-lysine
Protein-N-trimethyllysine
3.4.-.-
Trimethyl-lysine
1.14.11.8
N6-Hydroxy-trimethyllysine
4.1.2.-
4-Trimethyl-ammoniobutanal
Glycine
1.2.1.3
1.2.1.47
4-Trimethyl-ammoniobutanoate
1.14.11.1
Carnitine
1.14.11.4
2.4.1.50
Protein 5-galactosyloxylysine
Protein erythro-5-hydroxylysine
00310 2/13/14
(c) Kanehisa Laboratories

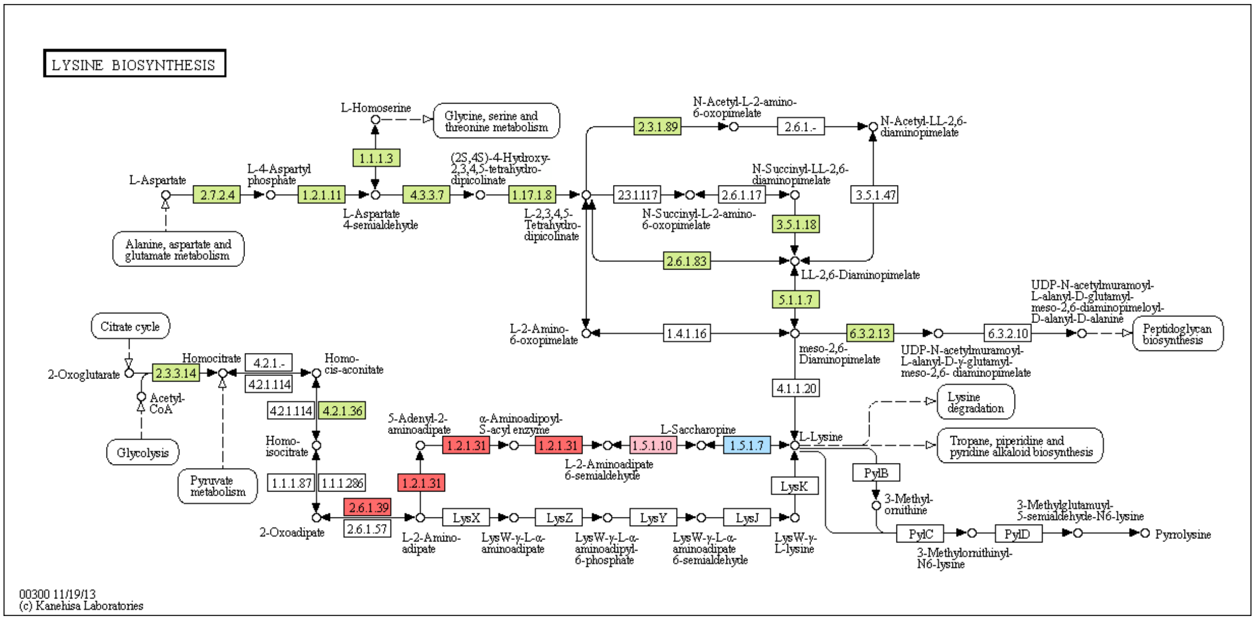
LYSINE BIOSYNTHESIS
L-Aspartate
L-4-Aspartyl phosphate
L-Homoserine
Glycine, serine and threonine metabolism
L-Aspartate 4-semialdehyde
(2S,4S)-4-Hydroxy-2,3,4,5-tetrahydro-dipicolinate
L-2,3,4,5-Tetrahydro-dipicolinate
N-Acetyl-L-2-amino-6-oxopimelate
N-Acetyl-LL-2,6-diaminopimelate
N-Succinyl-LL-2,6-diaminopimelate
N-Succinyl-L-2-amino-6-oxopimelate
LL-2,6-Diaminopimelate
Alanine, aspartate and glutamate metabolism
Citrate cycle
2-Oxoglutarate
Homocitrate
Homo-cis-aconitate
Acetyl-CoA
Glycolysis
Pyruvate metabolism
Homo-isocitrate
2-Oxoadipate
L-2-Amino-6-oxopimelate
meso-2,6-Diaminopimelate
UDP-N-acetylmuramoyl-L-alanyl-D-γ-glutamyl-meso-2,6-diaminopimelate
UDP-N-acetylmuramoyl-L-alanyl-D-glutamyl-meso-2,6-diaminopimeloyl-D-alanyl-D-alanine
Peptidoglycan biosynthesis
5-Adenyl-2-aminoadipate
α-Aminoadipoyl-S-acyl enzyme
L-Saccharopine
L-2-Aminoadipate 6-semialdehyde
L-Lysine
Lysine degradation
Tropane, piperidine and pyridine alkaloid biosynthesis
3-Methyl-ornithine
3-Methylornithinyl-N6-lysine
3-Methylglutamuyl-5-semialdehyde-N6-lysine
Pyrrolysine
L-2-Amino-adipate
LysW-γ-L-α-aminoadipate
LysW-γ-L-α-aminoadipyl-6-phosphate
LysW-γ-L-α-aminoadipate 6-semialdehyde
LysW-γ-L-lysine
2.7.2.4
1.2.1.11
1.1.1.3
4.3.3.7
1.17.1.8
2.3.1.89
2.6.1.-
2.3.1.117
2.6.1.17
3.5.1.18
3.5.1.47
2.6.1.83
5.1.1.7
1.4.1.16
6.3.2.13
6.3.2.10
4.1.1.20
2.3.3.14
4.2.1.-
4.2.1.114
4.2.1.36
1.1.1.87
1.1.1.286
2.6.1.39
2.6.1.57
1.2.1.31
1.5.1.10
1.5.1.7
LysX
LysZ
LysY
LysJ
LysK
PylB
PylC
PylD
00300 11/19/13
(c) Kanehisa Laboratories

OXIDATIVE PHOSPHORYLATION

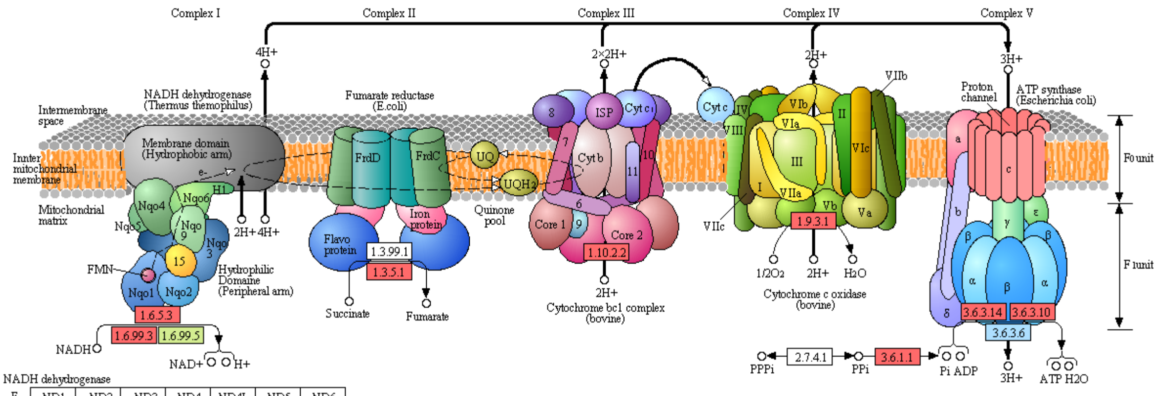

NADH dehydrogenase

E	ND1	ND2	ND3	ND4	ND4L	ND5	ND6										
E	Ndufs1	Ndufs2	Ndufs3	Ndufs4	Ndufs5	Ndufs6	Ndufs7	Ndufs8	Ndufv1	Ndufv2	Ndufv3						
B/A	NuoA	NuoB	NuoC	NuoD	NuoE	NuoF	NuoG	NuoH	NuoI	NuoJ	NuoK	NuoL	NuoM	NuoN			
B/A	NdhC	NdhK	NdhJ	NdhH	NdhA	NdhI	NdhG	NdhE	NdhF	NdhD	NdhB	NdhL	NdhM	NdhN	HoxE	HoxF	HoxU
E	Ndufa1	Ndufa2	Ndufa3	Ndufa4	Ndufa5	Ndufa6	Ndufa7	Ndufa8	Ndufa9	Ndufa10	Ndufab1	Ndufa11	Ndufa12	Ndufa13			
E	Ndufb1	Ndufb2	Ndufb3	Ndufb4	Ndufb5	Ndufb6	Ndufb7	Ndufb8	Ndufb9	Ndufb10	Ndufb11	Ndufc1	Ndufc2				

F-type ATPase (Bacteria)

beta	alpha	gamma	delta	epsilon	c	a	b

F-type ATPase (Eukaryotes)

beta	alpha	gamma	OSCP	delta	epsilon	c	a
b	e	f6	f	8			
d	f	h	j	k	g		

V-type ATPase (Prokaryotes)

A	B	C	D	E	F	I	K

V-type ATPase (Eukaryotes)

A	B	C	D	E	F	G	H
I	AC39	54kD	S1	lipid			

Succinate dehydrogenase / Fumarate reductase

E	SDHC	SDHD	SDHA	SDHB		
B/A	SdhC	SdhD	SdhA	SdhB		
			FrdA	FrdB	FrdC	FrdD

Cytochrome c reductase

E/B/A	ISP	Cyt b	Cyt 1						
E				COR1	QCR2	QCR6	QCR7	QCR8	QCR9	QCR10

Cytochrome c oxidase

E	COX10		COX3	COX1	COX2	COX4	COX5A	COX5B	COX6A	COX6B	COX6C	COX7A	COX7B	COX7C	COX8	E/B/A: COX11, COX15	COX17
B/A	CyoE	CyoD	CyoC	CyoB	CyoA												
		CoxD	CoxC	CoxA	CoxB												
		QoxD	QoxC	QoxB	QoxA												

Cytochrome c oxidase , cbb3-type

B	I	II	IV	III

Cytochrome bd complex

B/A	CydA	CydB

00190 6/1/12

(c) Kanehisa Laboratories

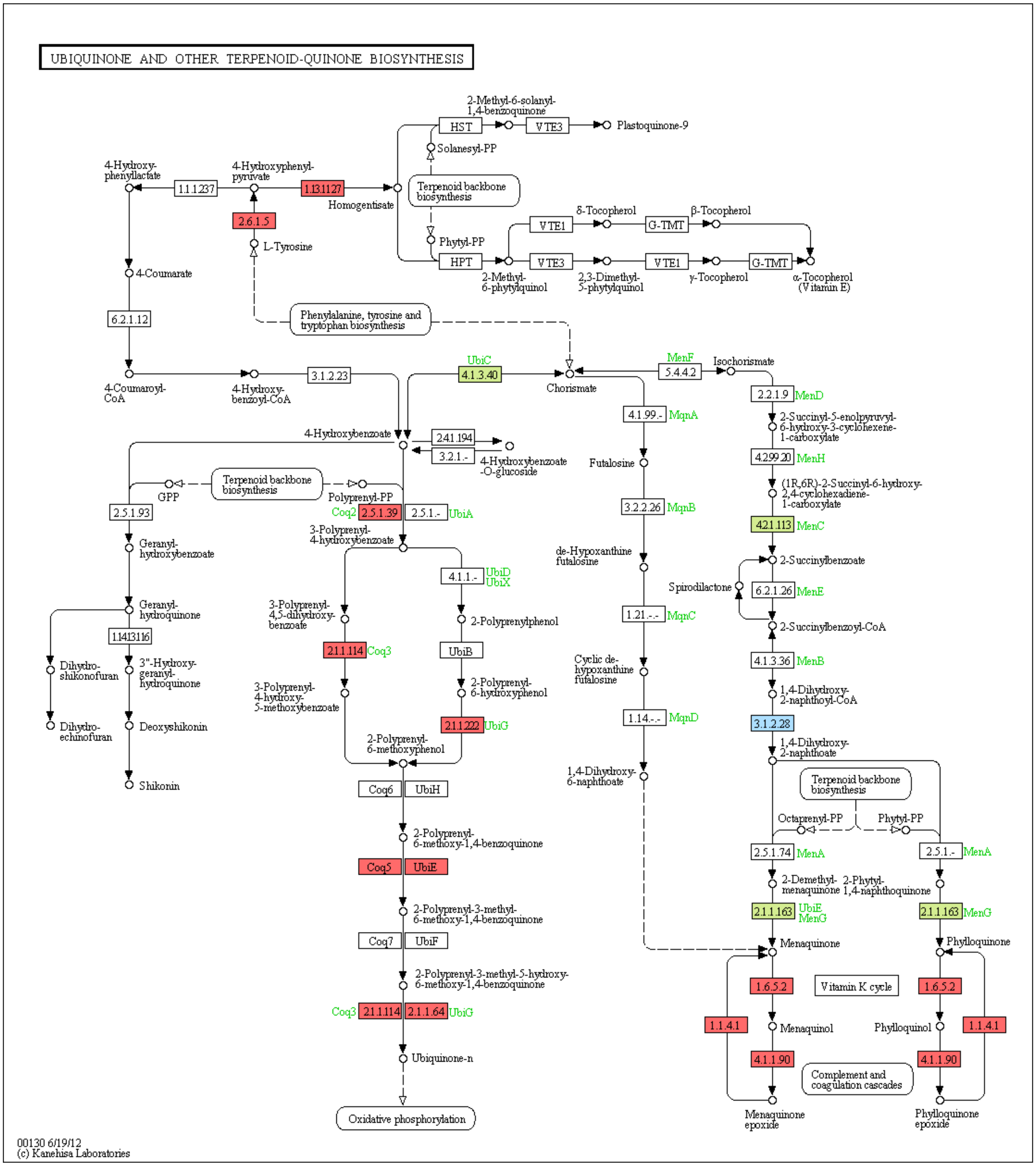
UBIQUINONE AND OTHER TERPENOID-QUINONE BIOSYNTHESIS
2-Methyl-6-solanyl-1,4-benzoquinone
HST
VTE3
Plastoquinone-9
Solanesyl-PP
4-Hydroxy-phenyllactate
1.1.1.237
4-Hydroxyphenyl-pyruvate
1.13.11.27
Homogentisate
Terpenoid backbone biosynthesis
2.6.1.5
L-Tyrosine
δ-Tocopherol
β-Tocopherol
VTE1
G-TMT
Phytyl-PP
HPT
VTE3
VTE1
G-TMT
2-Methyl-6-phytylquinol
2,3-Dimethyl-5-phytylquinol
γ-Tocopherol
α-Tocopherol (Vitamin E)
4-Coumarate
6.2.1.12
Phenylalanine, tyrosine and tryptophan biosynthesis
UbiC
4.1.3.40
MenF
5.4.4.2
Isochorismate
4-Coumaroyl-CoA
4-Hydroxy-benzoyl-CoA
3.1.2.23
Chorismate
2.2.1.9
MenD
4.1.99.-
MqnA
2-Succinyl-5-enolpyruvyl-6-hydroxy-3-cyclohexene-1-carboxylate
4-Hydroxybenzoate
2.4.1.194
3.2.1.-
4-Hydroxybenzoate-O-glucoside
Futalosine
4.2.99.20
MenH
(1R,6R)-2-Succinyl-6-hydroxy-2,4-cyclohexadiene-1-carboxylate
Terpenoid backbone biosynthesis
GPP
Polyprenyl-PP
2.5.1.93
Coq2
2.5.1.39
2.5.1.-
UbiA
3.2.2.26
MqnB
4.2.1.113
MenC
Geranyl-hydroxybenzoate
3-Polyprenyl-4-hydroxybenzoate
de-Hypoxanthine futalosine
2-Succinylbenzoate
4.1.1.-
UbiD
UbiX
Spirodilactone
6.2.1.26
MenE
Geranyl-hydroquinone
3-Polyprenyl-4,5-dihydroxy-benzoate
2-Polyprenylphenol
1.21.-.-
MqnC
2-Succinylbenzoyl-CoA
1.14.13.116
2.1.1.114
Coq3
UbiB
Dihydro-shikonofuran
3''-Hydroxy-geranyl-hydroquinone
Cyclic de-hypoxanthine futalosine
4.1.3.36
MenB
3-Polyprenyl-4-hydroxy-5-methoxybenzoate
2-Polyprenyl-6-hydroxyphenol
1,4-Dihydroxy-2-naphthoyl-CoA
1.14.-.-
MqnD
Dihydro-echinofuran
Deoxyshikonin
2.1.1.222
UbiG
3.1.2.28
2-Polyprenyl-6-methoxyphenol
1,4-Dihydroxy-2-naphthoate
1,4-Dihydroxy-6-naphthoate
Shikonin
Coq6
UbiH
Terpenoid backbone biosynthesis
Octaprenyl-PP
Phytyl-PP
2-Polyprenyl-6-methoxy-1,4-benzoquinone
2.5.1.74
MenA
2.5.1.-
MenA
Coq5
UbiE
2-Demethyl-menaquinone
2-Phytyl-1,4-naphthoquinone
2-Polyprenyl-3-methyl-6-methoxy-1,4-benzoquinone
2.1.1.163
UbiE
MenG
2.1.1.163
MenG
Coq7
UbiF
Menaquinone
Phylloquinone
2-Polyprenyl-3-methyl-5-hydroxy-6-methoxy-1,4-benzoquinone
1.6.5.2
Vitamin K cycle
1.6.5.2
Coq3
2.1.1.114
2.1.1.64
UbiG
1.1.4.1
Menaquinol
Phylloquinol
1.1.4.1
Ubiquinone-n
4.1.1.90
Complement and coagulation cascades
4.1.1.90
Menaquinone epoxide
Phylloquinone epoxide
Oxidative phosphorylation
00130 6/19/12
(c) Kanehisa Laboratories
